# PanGIA: A universal framework for identifying association between ncRNAs and diseases

**DOI:** 10.1093/gigascience/giaf123

**Published:** 2025-10-17

**Authors:** Xiaoyuan Liu, Xiye Lü, Qiuhao Chen, Jiqiu Sun, Tianyi Zhao, Yan Zhu

**Affiliations:** School of Medicine and Health, Harbin Institute of Technology, Harbin 150000, China; School of Medicine and Health, Harbin Institute of Technology, Harbin 150000, China; Zhengzhou Research Institute, Harbin Institute of Technology, Harbin 150000, China; Department of Otorhinolaryngology, Harbin Institute of Technology Hospital, Harbin 150038, China; School of Medicine and Health, Harbin Institute of Technology, Harbin 150000, China; Zhengzhou Research Institute, Harbin Institute of Technology, Harbin 150000, China; College of Veterinary Medicine, Northeast Agricultural University, Harbin 150038, China

**Keywords:** heterogeneous graph attention network, mixture of experts, cross-task attention mechanism, ncRNA–disease association

## Abstract

**Background:**

With the growing recognition of the important roles noncoding RNAs (ncRNAs) play in various biological functions, especially their potential involvement in many human diseases, predicting ncRNA–disease associations has become a key challenge in biomedical research.

**Results:**

Although many computational methods have been proposed to predict ncRNA–disease associations, most of these methods focus on a single type of ncRNA. However, the competitive and cooperative interactions among different types of ncRNAs are closely related to their functional roles in disease associations. To address this limitation, we propose a novel computational framework, **PanGIA** (Pan-ncRNA Graph-Interaction Attention network), designed to simultaneously predict potential associations between multiple types of noncoding RNAs, including microRNAs (miRNAs), long noncoding RNAs (lncRNAs), circular RNAs (circRNAs), and PIWI-interacting RNAs (piRNAs), and diseases. Experimental results show that PanGIA outperforms type-specific SOTA methods in both individual and comprehensive predictions. It remains robust even when nodes or ncRNA types are removed, and ablation studies confirm the benefits of cross-type information. PanGIA also outperforms several single-type state-of-the-art methods across multiple metrics.

**Conclusions:**

PanGIA demonstrates significant advantages in predicting disease associations for different types of ncRNAs, including miRNAs, lncRNAs, circRNAs, and piRNAs. Case studies further confirm the accuracy of the model’s predictions, as all high-confidence associations were supported by literature evidence. This demonstrates the model’s strong biological interpretability and promising potential for practical applications. The successful application of PanGIA provides a new paradigm for exploring disease-associated ncRNAs, highlighting their immense potential in the field of biomedical research.

## Introduction

Noncoding RNAs (ncRNAs) refer to a class of RNA molecules that do not encode proteins but play crucial roles in various biological processes, such as posttranscriptional regulation, epigenetic modification, and cellular signaling. In recent years, with the advancement of high-throughput sequencing technologies and functional genomics, an increasing number of ncRNAs have been identified as closely associated with a wide range of complex human diseases [1–4]. A growing body of experimental evidence has demonstrated that aberrant expression or dysfunction of ncRNAs is involved in the pathogenesis of major diseases, including cancer, neurodegenerative disorders, and cardiovascular diseases. Therefore, uncovering the potential associations between ncRNAs and diseases not only helps to elucidate the molecular mechanisms underlying complex diseases but also provides theoretical support for early diagnosis, biomarker discovery, and personalized treatment strategies. In particular, ncRNA regulatory mechanisms have emerged as a research hotspot in fields such as oncology, neurological disorders, and cardiovascular disease.

Among the various types of ncRNAs, small RNAs such as microRNAs (miRNAs) have been extensively studied and are well recognized for their posttranscriptional silencing functions through binding to target mRNAs. They have demonstrated significant potential as biomarkers in a wide range of diseases [5, 6].

Circular RNAs (circRNAs), owing to their covalently closed-loop structures that confer high stability, can function as competitive endogenous RNAs (ceRNAs) for microRNAs (miRNAs) or interact with RNA-binding proteins. Increasing evidence has demonstrated that circRNAs play critical regulatory roles and possess considerable potential for clinical applications across various disease contexts [7, 8].

Long ncRNAs (lncRNAs), which function by interacting with DNA, RNA, or proteins, are involved in processes such as chromatin modification and transcriptional regulation, and they have been found to play crucial roles in tumorigenesis, cell proliferation, and immune modulation [9, 10].

PIWI-interacting RNAs (piRNAs), initially thought to function predominantly in germ cells by suppressing transposable elements to maintain genome stability, have more recently been shown to exert regulatory functions in somatic cells as well. These piRNAs are increasingly associated with various cancers and metabolic disorders [11, 12].

Despite their critical roles in gene regulation and disease mechanisms, experimental identification of ncRNA–disease associations remains costly and time-consuming, limiting its scalability for large-scale studies. As a result, computational methods have gained increasing attention for their ability to efficiently and cost-effectively predict ncRNA–disease associations.

For miRNA, representative methods such as IMCMDA [13] leverage an integrated similarity network combined with a bilateral diffusion model to predict potential disease–miRNA associations. Regarding lncRNAs, LncDisAP [14] incorporates multiple similarity features and employs deep representation learning to effectively uncover latent associations. In the case of piRNAs, IPiDA-GBNN [15] enhances predictive accuracy by integrating graph neural networks with multifeature representations. For circRNAs, existing approaches include GCNCDA [16], which constructs a heterogeneous graph structure and applies graph convolutional networks to learn circRNA–disease relationships. Additionally, CRBPSA [17] exploits sequence- and structure-aware attention mechanisms to identify circRNA–RBP (RNA binding protein) interaction sites, thereby offering new insights into circRNA functionality. More recently, StackCirRNAPred [18] adopts a stacked ensemble learning strategy to achieve accurate classification of long circRNAs and other lncRNAs by integrating features from multiple sources.

Although these methods have achieved promising results within their respective ncRNA categories, they typically focus on a single type of ncRNA, ignoring the complex interplay and competition among different ncRNAs. For instance, miRNAs may interact with lncRNAs or circRNAs through the ceRNA mechanism, jointly regulating disease-related pathways. These interactions form a complex regulatory network, yet existing approaches lack comprehensive modeling of both cross-ncRNA relationships and multitype ncRNA–disease associations.

Therefore, there is a pressing need for novel computational frameworks that can jointly model the interrelations among various ncRNA types and their associations with diseases, enabling the discovery of previously unknown ncRNA–disease links through a more holistic understanding of their regulatory dynamics.

Despite the availability of several specialized repositories, such as miR2Disease, circR2Disease, LncRNADisease, and piRDisease, which systematically curate associations between ncRNAs and human diseases [19–22], these databases are inherently constrained by their reliance on experimentally derived evidence. The acquisition of such evidence is both resource-intensive and time-consuming, with its breadth inherently limited by laboratory conditions and prevailing research focuses. Moreover, most current studies are restricted to individual ncRNA classes, thereby neglecting potential crosstalk and cooperative interactions among distinct ncRNA species in disease pathogenesis. Consequently, existing resources remain insufficient to fully capture the complexity of ncRNA-mediated regulatory networks.

In conventional studies, most computational approaches focus on a single type of ncRNA, such as miRNA, circRNA, lncRNA, or piRNA, and are typically tailored to the specific features and data types associated with that category. Examples include IMCMDA [13], LncDisAP [14], IPiDA-GBNN [15], and GCNCDA [16], among others. These models are generally built upon sequence information, structural properties, or expression profiles unique to the targeted ncRNA type. However, such type-specific methods exhibit clear limitations in their generalizability, as they are often not applicable to other classes of ncRNAs.

This limitation arises from the substantial differences in structure, biological function, and disease-related mechanisms among various ncRNA types. For instance, miRNAs primarily function through posttranscriptional repression by targeting mRNAs, whereas lncRNAs are involved in gene regulation and chromatin remodeling. CircRNAs are known to act as “sponges” for miRNAs, and piRNAs are mainly implicated in posttranscriptional regulation and transposon silencing. Consequently, models focusing exclusively on one ncRNA type tend to ignore the potential interactions and synergies among different ncRNA categories, thereby restricting their applicability and limiting their potential to uncover cross-type regulatory mechanisms in disease contexts.

Furthermore, single-type RNA-based approaches are inadequate in capturing the complex biological interactions that may exist across different RNA types. For example, miRNAs may indirectly influence disease development by regulating lncRNA expression, circRNAs may impact disease progression through interactions with miRNAs, and piRNAs may engage with other RNA species in various biological processes. Since traditional methods are confined to individual RNA types, they are unable to fully elucidate the potential cross-talk and coregulatory mechanisms among diverse ncRNA classes.

Therefore, there is an urgent need to develop an efficient and scalable computational prediction model capable of systematically identifying potential associations between different types of ncRNAs and diseases. Such a model would not only compensate for the limitations of experimental data but also expand the knowledge graph of disease regulatory networks.

In this article, we propose **PanGIA** (Pan-ncRNA Graph-Interaction Attention network), a novel framework for comprehensive ncRNA–disease association prediction. To address the challenge of feature heterogeneity, PanGIA constructs a heterogeneous graph that integrates multisource data, including sequence information, functional similarity, and interaction networks of ncRNAs. To capture comprehensive and layered associations, it employs a cross-task attention mechanism combined with a mixture-of-experts architecture to dynamically learn the multilevel interactions between ncRNAs and diseases. Notably, PanGIA encompasses 4 representative classes of noncoding RNAs—miRNAs, lncRNAs, circRNAs, and piRNAs—which exhibit distinct characteristics in terms of length, structure, regulatory mechanisms, and functional roles. These classes, owing to their complementary and representative nature in current research, are collectively referred to as pan-ncRNAs [23]. By adopting a pan-ncRNA perspective, PanGIA not only overcomes the limitations of single-type ncRNA studies but also provides a more comprehensive understanding of the multilayered regulatory roles of ncRNAs in disease.

The main contributions of our work are summarized as follows:

- **Pan-ncRNA integration:** PanGIA jointly models 4 types of ncRNAs (miRNA, lncRNA, circRNA, and piRNA), overcoming the limitations of single-type approaches.
- **Heterogeneous graph fusion:** It constructs a heterogeneous graph to integrate sequence, semantic, and functional data, capturing complex ncRNA–disease relationships.
- **Cross-task attention:** A mixture-of-experts module with cross-task attention enhances feature sharing across ncRNA types and improves prediction accuracy.
- **Superior performance:** PanGIA achieves higher area under the curve (AUC), area under the precision-recall curve (AUPR), and rank metrics than baseline models, demonstrating strong generalization and reliability.

## Materials

Our study involves multiple classes of noncoding RNAs and requires the simultaneous acquisition of their sequence information and disease association data. The databases utilized in this study are listed as follows:

- **miRNA:** The associations between miRNAs and diseases were obtained from the HMDD v4.0 database [24], while the sequence information of miRNAs was retrieved from the miRBase database [25].
- **LncRNA/circRNA:** This study includes lncRNA and circRNA associations with diseases, with data obtained from LncRNADisease v3.0 [26]. The sequence information of circRNAs was retrieved from the circBase database [27]. In contrast, lncRNA sequences were collected from 2 sources: GENCODE [28] and NONCODE [29].
- **piRNA:** The associations between piRNAs and diseases were obtained from the piRDisease v1.0 [21] database, and the sequence information was retrieved from the piRBase [30] and piRNAdb [31] databases.
- **Disease:** This study utilizes Disease Ontology Identifiers (DOIDs) to construct the disease similarity matrix, with corresponding information obtained from the Disease Ontology database [32].

The construction of the ncRNA–disease association network was based on merging data entries from the aforementioned association databases.

## Methods

**Figure 1:**
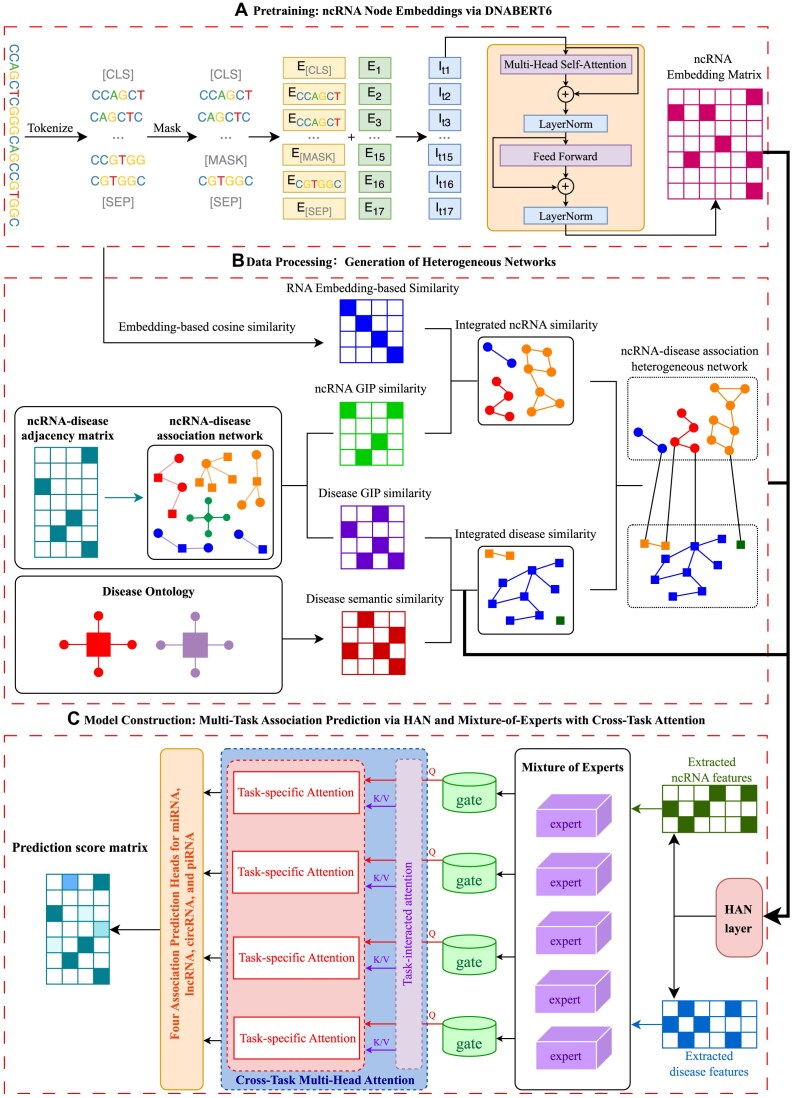
The structure of PanGIA.

We propose a novel model named **PanGIA**, which is built upon the heterogeneous graph attention network (HAN) and a mixture-of-experts (MoE) framework. The model is designed to predict associations between pan-ncRNAs and diseases. The overall workflow of **PanGIA** is illustrated in Fig. 1, and consists of 3 main steps:

- **Pretraining:** ncRNA node embeddings via DNABERT6
- **Data processing:** generation of heterogeneous networks
- **Model construction:** multitask association prediction via HAN and MoE with cross-task attention

### Pretraining: ncRNA node embeddings via DNABERT6

In this study, we leveraged the DNABERT6 [33] model to pretrain ncRNA sequences and obtain high-quality node embeddings. DNABERT represents a recent class of methods that adapt the BERT architecture, originally developed in natural language processing, to genomic sequence modeling. The central idea is to segment DNA sequences into fixed-length *k*-mers, thereby treating the genomic sequence as a special type of “language.” By pretraining on large-scale genomic corpora, DNABERT is able to capture contextual dependencies within sequences and has demonstrated superior performance compared to traditional feature engineering approaches in a variety of downstream tasks, such as promoter prediction and transcription factor binding site identification. Building on this concept, we employed the DNABERT6 variant (with *k* = 6) to model ncRNA sequences, thereby generating embeddings suitable for subsequent graph-based representation learning. The overall workflow is illustrated in Fig. 1A and can be summarized in the following steps:

#### Tokenization of ncRNA sequences

We first segmented the ncRNA sequences into fixed-length *k*-mers of size 6. Prior to tokenization, all uracil (U) bases in the RNA sequences were systematically replaced with thymine (T) to ensure compatibility with DNA-based models such as DNABERT. The fundamental rationale of *k*-mer tokenization is to conceptualize DNA/RNA sequences as a specialized form of “language,” in which each nucleotide fragment of length 6 serves as an independent lexical unit. Previous studies have demonstrated that setting *k* = 6 provides superior performance across a wide range of genomic modeling tasks, as it effectively preserves local sequence features while simultaneously capturing long-range dependencies [33–35]. This strategy establishes a solid foundation for subsequent deep representation learning.

#### Masked language model pretraining

After obtaining the 6-mer token sequences, we employed a masked language model (MLM) pretraining strategy, wherein a subset of tokens was randomly masked, and the model was required to recover the original tokens based on their surrounding context. Specifically, certain *k*-mer tokens in the input sequence were randomly replaced by a mask symbol, and the model was trained to reconstruct the original tokens conditioned on the unmasked context, thereby enabling the learning of contextual dependencies within the sequence. The corresponding objective function can be formally expressed as


(1) \begin{eqnarray*}
\mathcal {L}_{\text{MLM}} = - \sum _{i \in \mathcal {M}} \log P(x_i \mid x_{\setminus \mathcal {M}}; \theta ),
\end{eqnarray*}

where $\mathcal {M}$ denotes the set of masked positions, $x_i$ is the masked token, $x_{\setminus \mathcal {M}}$ represents the unmasked context tokens, and $\theta$ denotes the model parameters.

From a biological perspective, this mechanism facilitates the identification of potential functional motifs and enhances the ability to capture their regulatory roles under diverse contextual environments.

#### Embedding representation

At the input layer, each 6-mer token is mapped into a dense vector representation composed of 3 components:

- **Token embedding:** captures the semantic features of the current 6-mer
- **Positional embedding:** encodes the positional information of each token within the sequence, thereby enabling the model to preserve the linear order of nucleotides
- **Segment embedding:** used to differentiate between distinct segments in concatenated sequences

Formally, the overall embedding of a token can be expressed as


(2) \begin{eqnarray*}
\mathbf {e}_i = \mathbf {e}_i^{\text{token}} + \mathbf {e}_i^{\text{pos}} + \mathbf {e}_i^{\text{seg}},
\end{eqnarray*}

where $\mathbf {e}_i^{\text{token}}$, $\mathbf {e}_i^{\text{pos}}$, and $\mathbf {e}_i^{\text{seg}}$ denote the token, positional, and segment embeddings of the *i*th token, respectively.

This multilevel embedding strategy enables the model to simultaneously retain local nucleotide features while capturing the global topological structure of the sequence, thereby providing a comprehensive representation for downstream tasks.

#### Transformer encoding and ncRNA embedding matrix

After obtaining the token-level embeddings, the sequence representations are fed into a stack of Transformer encoders. The core component of the Transformer is the multihead self-attention mechanism, which enables the model to capture dependencies among sequence fragments in multiple subspaces. For ncRNA sequences, this mechanism is particularly important, as functional elements such as binding sites or seed regions may exhibit interactions spanning long distances along the sequence. By stacking multiple layers of self-attention and feed-forward networks, the model is able to generate increasingly rich contextual representations.

Ultimately, the model integrates the contextual information of each sequence into an embedding matrix that not only captures local nucleotide fragment features but also encodes long-range dependencies and global sequence semantics. Compared with traditional one-hot encoding or manually engineered sequence features, the embeddings generated by DNABERT are more comprehensive and robust. For RNA nodes, we employ DNABERT embeddings of 768 dimensions as the input features. This embedding matrix is further utilized as the node feature representation in graph-based learning, thereby providing a high-quality foundation for predicting ncRNA–disease associations.

### Data processing: generation of heterogeneous networks

#### Heterogeneous graph construction

In this study, we first preprocessed the raw ncRNA sequence data and their known associations with diseases in order to construct a cross-modal heterogeneous network, which serves as the input foundation for the PanGIA model. Formally, the heterogeneous graph is defined as


(3) \begin{eqnarray*}
\mathcal {G} = (\mathcal {V}, \mathcal {E}),
\end{eqnarray*}

where the node set is given by


(4) \begin{eqnarray*}
\mathcal {V} = \mathcal {V}_{\text{RNA}} \cup \mathcal {V}_{\text{dis}},
\end{eqnarray*}

with $\mathcal {V}_{\text{RNA}}$ denoting the set of RNA nodes and $\mathcal {V}_{\text{dis}}$ denoting the set of disease nodes.

The edge set $\mathcal {E}$ is composed of multiple types of relationships, including the following:

- **RNA–disease associations:**
 
  (5) \begin{eqnarray*}
\mathcal {E}_{\text{RNA-dis}} = \lbrace (r_i, d_j) \mid A_{ij} = 1 \rbrace ,
\end{eqnarray*}
  where $A \in \lbrace 0,1\rbrace ^{|\mathcal {V}_{\text{RNA}}| \times |\mathcal {V}_{\text{dis}}|}$ represents the known RNA–disease association matrix. If $A_{ij}=1$, this indicates that RNA node $r_i$ is associated with disease node $d_j$.
- **RNA–RNA similarity edges:**
 
  (6) \begin{eqnarray*}
\mathcal {E}_{\text{RNA-RNA}} = \lbrace (r_i, r_j, w^{\text{RNA}}_{ij}) \mid S^{\text{RNA}}_{ij} > 0 \rbrace ,
\end{eqnarray*}
  where $S^{\text{RNA}} \in \mathbb {R}^{|\mathcal {V}_{\text{RNA}}| \times |\mathcal {V}_{\text{RNA}}|}$ denotes the RNA similarity matrix. A weighted edge is established between 2 RNA nodes if their similarity score is greater than zero, with the edge weight denoted as $w^{\text{RNA}}_{ij}$.
- **Disease–disease similarity edges:**
 
  (7) \begin{eqnarray*}
\mathcal {E}_{\text{dis-dis}} = \lbrace (d_i, d_j, w^{\text{dis}}_{ij}) \mid S^{\text{dis}}_{ij} > 0 \rbrace ,
\end{eqnarray*}
  where $S^{\text{dis}} \in \mathbb {R}^{|\mathcal {V}_{\text{dis}}| \times |\mathcal {V}_{\text{dis}}|}$ denotes the disease similarity matrix. Similarly, if the similarity score between 2 diseases is greater than zero, a weighted edge is constructed with weight $w^{\text{dis}}_{ij}$.

#### Construction of ncRNA similarity matrices

To model intraclass similarities among 4 major categories of ncRNAs—miRNA, lncRNA, circRNA, and piRNA—we constructed similarity matrices based on embedding representations rather than conventional sequence alignment. Specifically, each ncRNA sequence was encoded into a dense embedding vector $\mathbf {h}_i \in \mathbb {R}^d$ using a pretrained model. The similarity between any 2 sequences $x_i$ and $x_j$ of the same category was then quantified via cosine similarity:


(8) \begin{eqnarray*}
\text{sim}(x_i, x_j) = \frac{\mathbf {h}_i \cdot \mathbf {h}_j}{\Vert \mathbf {h}_i\Vert \, \Vert \mathbf {h}_j\Vert },
\end{eqnarray*}

where $\mathbf {h}_i$ and $\mathbf {h}_j$ denote the embedding vectors of sequences $x_i$ and $x_j$, respectively. After normalization, the similarity values were constrained within the interval [0,1], thereby ensuring consistency across different ncRNA types.

Finally, the similarity matrices for all ncRNAs were organized into a block-diagonal structure:


(9) \begin{eqnarray*}
\mathbf {S}^{\text{seq}}_{\text{RNA}} = {\begin{bmatrix}S^{\text{seq}}_{\text{mi}} & 0 & 0 & 0 \\
0 & S^{\text{seq}}_{\text{circ}} & 0 & 0 \\
0 & 0 & S^{\text{seq}}_{\text{lnc}} & 0 \\
0 & 0 & 0 & S^{\text{seq}}_{\text{pi}} \end{bmatrix}},
\end{eqnarray*}

where $S^{\text{seq}}_{\text{mi}}, S^{\text{seq}}_{\text{circ}}, S^{\text{seq}}_{\text{lnc}},$ and $S^{\text{seq}}_{\text{pi}}$ correspond to the cosine similarity matrices of miRNA, circRNA, lncRNA, and piRNA, respectively. This block-diagonal representation provides a structured foundation for integrating multiclass ncRNA similarities into downstream graph-based learning.

Based on the constructed ncRNA–disease association network, we next compute the functional similarity of noncoding RNAs using the Gaussian Interaction Profile (GIP) kernel function. The corresponding formula is given as follows:


(10) \begin{eqnarray*}
\mathbf {S}_{\text{RNA}}^{\text{GIP}}(r_i, r_j) = \exp \left( -\lambda _{\text{RNA}} \left\Vert \mathbf {A}(r_i, :) - \mathbf {A}(r_j, :) \right\Vert ^2 \right)
\end{eqnarray*}

In this formulation, $\mathbf {A}(r_i, :)$ and $\mathbf {A}(r_j, :)$ represent the vectors corresponding to the *i*th and *j*th rows of the adjacency matrix $\mathbf {A}$, respectively. The parameter $\lambda _{\text{RNA}}$ denotes the bandwidth coefficient of the kernel function, which is defined as follows:


(11) \begin{eqnarray*}
\lambda _{\text{RNA}} = \frac{1}{\frac{1}{N_{\text{r}}} \sum _{k=1}^{N_{\text{r}}} \left\Vert \mathbf {A}(r_k, :) \right\Vert ^2}
\end{eqnarray*}

$N_r$ denotes the total number of ncRNAs, and $\mathbf {A}(r_k, :)$ represents the vector corresponding to the *k*th row of the adjacency matrix $\mathbf {A}$. Subsequently, we integrate the sequence similarity and GIP-based functional similarity to obtain the final ncRNA similarity matrix:


(12) \begin{eqnarray*}
\mathbf {S}^{\text{RNA}} = \frac{\mathbf {S}^{\text{seq}}_{\text{RNA}} + \mathbf {S}^{\text{GIP}}_{\text{RNA}}}{2}
\end{eqnarray*}

#### Construction of the disease similarity matrix

On the disease side, we constructed 2 types of disease similarity networks based on different approaches: (i) a semantic similarity matrix calculated using Disease Ontology and (ii) a GIP-based similarity matrix generated from disease interaction profiles. By integrating these 2 sources of similarity information, we obtained a comprehensive disease similarity network to enhance the accuracy of disease representation [36–38].

Disease Ontology is a structured ontology that organizes various diseases and their hierarchical relationships. Each disease node in the ontology is assigned a unique identifier and may be associated with descriptive attributes, such as symptoms and causes. The hierarchical structure of the ontology typically resembles a tree, where parent nodes represent broader disease categories and child nodes correspond to more specific diseases. In this study, we employ the Jaccard similarity coefficient to compute the semantic similarity matrix between diseases, defined as follows:


(13) \begin{eqnarray*}
\mathbf {S}_{\text{dis}}^{\text{sem}} = \frac{|\text{Ancestors}(d_1) \cap \text{Ancestors}(d_2)|}{|\text{Ancestors}(d_1) \cup \text{Ancestors}(d_2)|}
\end{eqnarray*}

In this formulation, $\text{Ancestors}(d)$ denotes the set of ancestor nodes of the disease node *d*, and $|A|$ represents the cardinality (i.e., the number of elements) of the set *A*.

The functional similarity of diseases based on the GIP kernel is calculated as follows:


(14) \begin{eqnarray*}
\mathbf {S}_{\text{dis}}^{\text{GIP}}(d_i, d_j) = \exp \left( -\lambda _{\text{dis}} \left\Vert \mathbf {A}(:, d_i) - \mathbf {A}(:, d_j) \right\Vert ^2 \right)
\end{eqnarray*}

In this formulation, $\mathbf {A}(:, d_i)$ and $\mathbf {A}(:, d_j)$ represent the vectors corresponding to the *i*th and *j*th columns of the adjacency matrix $\mathbf {A}$, respectively. The parameter $\lambda _{\text{dis}}$ denotes the bandwidth coefficient of the kernel function, which is defined as follows:


(15) \begin{eqnarray*}
\lambda _{\text{dis}} = \frac{1}{\frac{1}{N_{\text{d}}} \sum _{k=1}^{N_{\text{d}}} \left\Vert \mathbf {A}(:,d_k) \right\Vert ^2}
\end{eqnarray*}

$N_d$ denotes the total number of diseases. Subsequently, we integrate the semantic similarity and GIP-based functional similarity to obtain the final integrated disease similarity matrix:


(16) \begin{eqnarray*}
\mathbf {S}^{\text{dis}} = \frac{\mathbf {S}^{\text{sem}}_{\text{dis}} + \mathbf {S}^{\text{GIP}}_{\text{dis}}}{2}
\end{eqnarray*}

We integrate the constructed ncRNA similarity network, the disease similarity network, and the known ncRNA–disease associations to form a unified ncRNA–disease heterogeneous graph, denoted as $\mathcal {G}$.

### Model construction: multitask association prediction via HAN and MoE with cross-task attention

We propose a novel multitask relational prediction framework that integrates HAN with a MoE mechanism. Through a cross-task attention mechanism, the framework enables collaborative modeling across tasks, enhancing both task generalization and interaction expression capabilities. The overall structure of the model is depicted in Fig. 1C, which consists of the following 5 main components:

#### Feature representation and heterogeneous graph modeling

The model input consists of the ncRNA embedding matrix $\mathbf {X}^{\text{rna}} \in \mathbb {R}^{N_r \times d_r}$ and the disease embedding matrix $\mathbf {X}^{\text{dis}} \in \mathbb {R}^{N_d \times d_d}$, where $N_r$ and $N_d$ represent the number of ncRNAs and diseases, respectively, and $d_r$ and $d_d$ correspond to their embedding dimensions. Since the original feature dimensions of ncRNAs and diseases may differ, we first map the disease embeddings into the same space as the ncRNA embeddings:


(17) \begin{eqnarray*}
\mathbf {X}^{\text{dis}}_{\text{proj}} = \mathbf {X}^{\text{dis}} \mathbf {W}_d \in \mathbb {R}^{N_d \times d_r}
\end{eqnarray*}

where $\mathbf {W}_d \in \mathbb {R}^{d_d \times d_r}$ is a learnable linear transformation matrix.

Next, the heterogeneous network $\mathcal {N}_H$, based on ncRNA–disease associations, along with the ncRNA embedding matrix $\mathbf {X}^{\text{rna}}$ and disease embedding matrix $\mathbf {X}^{\text{dis}}$, is fed into the HAN model. A multihead attention mechanism, guided by meta-paths, is employed to extract higher-order semantic features from the graph structure. The output of the HAN encoder is


(18) \begin{eqnarray*}
\mathbf {H}^{\text{rna}}, \mathbf {H}^{\text{dis}} = \text{HANEncoder}(\mathbf {X}^{\text{rna}}, \mathbf {X}^{\text{dis}}_{\text{proj}}, \mathcal {N}_H)
\end{eqnarray*}

where $\mathbf {H}^{\text{rna}}, \mathbf {H}^{\text{dis}} \in \mathbb {R}^{N \times d_h}$ represent the hidden representations of ncRNAs and diseases extracted by the HAN layer, and $d_h$ denotes the intermediate hidden dimension.

#### Expert pool and global disease information fusion

To integrate global disease semantics, we average the representations of all disease nodes to obtain the global disease feature:


(19) \begin{eqnarray*}
\bar{\mathbf {H}}^{\text{dis}} = \frac{1}{N_d} \sum _{i=1}^{N_d} \mathbf {H}^{\text{dis}}_i \in \mathbb {R}^{d_h}
\end{eqnarray*}

We concatenate this with each ncRNA representation to form the fused representation:


(20) \begin{eqnarray*}
\mathbf {F} = \left[ \mathbf {H}^{\text{rna}} \, \Vert \, \bar{\mathbf {H}}^{\text{dis}} \right] \in \mathbb {R}^{N_r \times 2d_h}
\end{eqnarray*}

Subsequently, the fused representation is input into an expert pool consisting of $K$ experts, where each expert is a nonlinear transformation module:


(21) \begin{eqnarray*}
\mathcal {E}_k(\mathbf {F}) = \text{ReLU}(\mathbf {F} \mathbf {W}_k + \mathbf {b}_k), \quad k=1,\dots ,K
\end{eqnarray*}

The outputs of all experts are then stacked:


(22) \begin{eqnarray*}
\mathbf {E} = \text{stack}(\mathcal {E}_1(\mathbf {F}), \dots , \mathcal {E}_K(\mathbf {F})) \in \mathbb {R}^{N_r \times K \times d_e}
\end{eqnarray*}

where $d_e$ denotes the output dimension of each expert.

#### Multitask gating mechanism

For each specific task $t \in \lbrace 1, \dots , T\rbrace $, the corresponding ncRNA subset is $\mathcal {I}_t \subset \lbrace 1, \dots , N_r\rbrace $. We learn a gating network for each task to perform attention-based selection of experts in the expert pool:

- First, the task-related input representation $\mathbf {H}_t = \mathbf {H}^{\text{rna}}[\mathcal {I}_t]$ is mapped to a query vector $\mathbf {Q}_t$.
- The expert representations are mapped to keys $\mathbf {K}_t$ and values $\mathbf {V}_t$.
- A multihead attention mechanism is then used to compute the attention-weighted expert representation:

(23) \begin{eqnarray*}
\mathbf {A}_t = \text{softmax}(\text{MultiHeadAttn}(\mathbf {Q}_t, \mathbf {K}_t, \mathbf {V}_t)) \in \mathbb {R}^{n_t \times K}
\end{eqnarray*}

The final aggregation of the expert outputs results in the task feature representation:


(24) \begin{eqnarray*}
\mathbf {u}_t = \sum _{k=1}^K \mathbf {A}_{t, :, k} \cdot \mathbf {E}_{\mathcal {I}_t, k} \in \mathbb {R}^{n_t \times d_e}
\end{eqnarray*}

#### Cross-task attention interaction

To model the potential correlations between tasks, we input the aggregated representations of all tasks (after average pooling) into a cross-task multihead attention module:


(25) \begin{eqnarray*}
\mathbf {U} = [\bar{\mathbf {u}}_1, \dots , \bar{\mathbf {u}}_T] \in \mathbb {R}^{T \times d_e}, \quad \bar{\mathbf {u}}_t = \frac{1}{n_t} \sum _{i=1}^{n_t} \mathbf {u}_{t,i}
\end{eqnarray*}

(26) \begin{eqnarray*}
\mathbf {U}^{\prime } = \text{MultiHeadAttn}(\mathbf {U}, \mathbf {U}, \mathbf {U}) \in \mathbb {R}^{T \times d_e}
\end{eqnarray*}

The cross-task global representation $\mathbf {U}^{\prime }_t$ is then concatenated with the original task representation $\mathbf {u}_t$:


(27) \begin{eqnarray*}
\mathbf {z}_t = \text{ReLU}([\mathbf {u}_t \, \Vert \, \mathbf {U}^{\prime }_t]) \in \mathbb {R}^{n_t \times d_e}
\end{eqnarray*}

#### Relational prediction and output layer

The representations of all diseases are projected to the expert dimension through a linear transformation:


(28) \begin{eqnarray*}
\tilde{\mathbf {H}}^{\text{dis}} = \mathbf {H}^{\text{dis}} \mathbf {W}_{\text{proj}} \in \mathbb {R}^{N_d \times d_e}
\end{eqnarray*}

Finally, the association score between each ncRNA and all diseases for each task is calculated through the dot product, followed by normalization using the sigmoid function:


(29) \begin{eqnarray*}
\hat{\mathbf {Y}}_t = \sigma (\mathbf {z}_t \cdot \tilde{\mathbf {H}}^{\text{dis}^\top }) \in \mathbb {R}^{n_t \times N_d}
\end{eqnarray*}

In this framework, the association prediction task is formulated as a binary classification problem. For each task *t*, the task-specific MLP transforms the RNA representations into $\mathbf {z}_t$, which captures task-refined structural and semantic features. Each disease node representation is projected into the same latent space, yielding $\tilde{\mathbf {H}}^{\text{dis}}$. The association score between an RNA node *i* and a disease node *j* is computed as the dot product:


(30) \begin{eqnarray*}
s_{t,ij} = \mathbf {z}_{t,i} \cdot \tilde{\mathbf {h}}^{\text{dis}}_j.
\end{eqnarray*}

This score is then passed through a sigmoid function to produce the following probability:


(31) \begin{eqnarray*}
\hat{y}_{t,ij} = \sigma (s_{t,ij}) \in [0,1],
\end{eqnarray*}

which indicates the likelihood that RNA *i* is associated with disease *j*. Hence, the MLP does not serve as the final classifier but rather as a task-dependent feature extractor, while the prediction itself is achieved through the interaction between RNA and disease embeddings.

## Results

### Benchmark on various ncRNAs

In this study, we evaluated the performance of different models using 5-fold cross-validation and employed Rank Index, AUC, and AUPR as evaluation metrics.

Under the evaluation of 5-fold cross-validation, the performance of various models on the pan-ncRNA–disease association prediction task is summarized in Table 1. Compared with the baseline methods, PanGIA consistently achieved the best performance across multiple evaluation metrics.

**Table 1.** Performance comparison of PanGIA and baseline models on ncRNA–disease association prediction tasks

Model	RNA category	AUC	Rank Index	AUPR
NIMGSA [39]	miRNA	0.947 $\pm$ 0.003	0.318 $\pm$ 0.006	0.682 $\pm$ 0.002
MINIMDA [40]	miRNA	0.918 $\pm$ 0.001	0.324 $\pm$ 0.003	0.904 $\pm$ 0.004
PanGIA	miRNA	0.926 $\pm$ 0.003	0.304 $\pm$ 0.003	0.914 $\pm$ 0.002
gGATLDA [41]	lncRNA	0.931 $\pm$ 0.001	0.283 $\pm$ 0.002	0.923 $\pm$ 0.005
LDGRNMF [42]	lncRNA	0.892 $\pm$ 0.005	0.328 $\pm$ 0.003	0.849 $\pm$ 0.004
PanGIA	lncRNA	0.933 $\pm$ 0.006	0.298 $\pm$ 0.001	0.927 $\pm$ 0.003
iPiDi-PUL [43]	piRNA	0.569 $\pm$ 0.026	0.444 $\pm$ 0.021	0.117 $\pm$ 0.008
PUTransGCN [44]	piRNA	0.930 $\pm$ 0.007	0.103 $\pm$ 0.006	0.598 $\pm$ 0.032
PanGIA	piRNA	0.934 $\pm$ 0.001	0.291 $\pm$ 0.007	0.929 $\pm$ 0.003
IGNSCDA [45]	circRNA	0.812 $\pm$ 0.003	0.331 $\pm$ 0.004	0.694 $\pm$ 0.006
GATCL2CD [46]	circRNA	0.931 $\pm$ 0.004	0.282 $\pm$ 0.007	0.879 $\pm$ 0.008
PanGIA	circRNA	0.927 $\pm$ 0.005	0.306 $\pm$ 0.003	0.914 $\pm$ 0.009
PanGIA	miRNA, lncRNA, piRNA, circRNA	0.988 $\pm$ 0.002	0.256 $\pm$ 0.001	0.985 $\pm$ 0.004

To comprehensively evaluate the performance of the PanGIA model in multitype ncRNA–disease association prediction, this study conducted comparative experiments using various existing mainstream methods across 4 types of ncRNAs—miRNA, lncRNA, piRNA, and circRNA. The models’ performance was assessed using AUC, AUPR, and Rank Index metrics. As shown in the table, PanGIA outperformed all other methods across all RNA types. Furthermore, when all ncRNA types were integrated, the model’s performance was further enhanced, achieving the highest AUC and AUPR and the lowest Rank Index. These results strongly demonstrate PanGIA’s exceptional generalization ability and predictive accuracy in multitype ncRNA–disease association prediction.

### Multitask synchronous prediction can provide key information

To validate the advantages of our proposed framework in leveraging the full-spectrum heterogeneous association network and the neural network architecture design, we not only examined the performance after ablating critical network modules but also progressively reduced the scale of full-spectrum data to evaluate the unique contribution of pan-ncRNA–disease association information.

#### Stepwise reduction of heterogeneity in the network

To systematically evaluate the impact of reduced training data on model performance, we designed 2 downsampling strategies to progressively decrease the amount of information available to the PanGIA model: (i) a random uniform subsampling strategy and (ii) an RNA-type-based node selection strategy.

In the random uniform subsampling strategy, we adopted a straightforward uniform sampling method. Specifically, a certain proportion of ncRNA and disease nodes were randomly and uniformly removed from the heterogeneous graph, along with their associated edges. This ensured that both types of nodes (ncRNAs and diseases) were reduced at the same rate, preserving the relative balance between modalities in the network while decreasing the overall graph size. By gradually scaling down the input network, we simulated scenarios with limited data availability to examine how PanGIA performs under constrained information settings.

**Figure 2:**
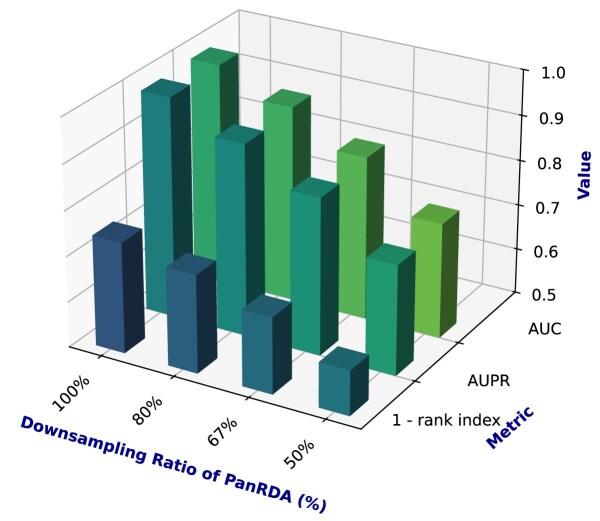
Performance comparison of PanGIA under different subsampling ratios.

Based on this strategy, we conducted a systematic performance evaluation of the PanGIA model using 100%, 80%, 67%, and 50% of the original dataset for training. As shown in Fig. 2, the corresponding evaluation metrics—AUC, AUPR, and Rank Index—consistently declined with reduced data scale. These results indicate that PanGIA is sensitive to the quantity of training data and that its predictive capability is notably affected under data-sparse conditions.

In summary, this experiment underscores the critical importance of data completeness in achieving optimal predictive performance with PanGIA, highlighting the key role of full-spectrum biological data in robust association prediction tasks.

**Figure 3:**
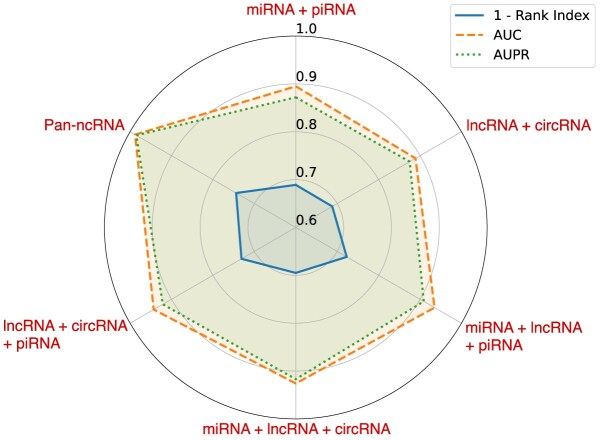
Performance of PanGIA with different ncRNA combinations.

To further evaluate the overall contribution of different ncRNA types to the predictive performance of the model, we conducted a stepwise ablation study by progressively removing specific categories of ncRNAs from the full pan-ncRNA set. As shown in Fig. 3, we assessed model performance under various ncRNA combinations using AUC, AUPR, and Rank Index as evaluation metrics. The results demonstrate that the inclusion of all 4 ncRNA types (miRNA, lncRNA, circRNA, and piRNA) yields the best overall performance. In contrast, removing any single or multiple ncRNA types leads to a noticeable decline in 1 or more metrics, highlighting the complementary contributions of each ncRNA class to the overall prediction capability of the PanGIA framework.

### Robustness of PanGIA

**Figure 4:**
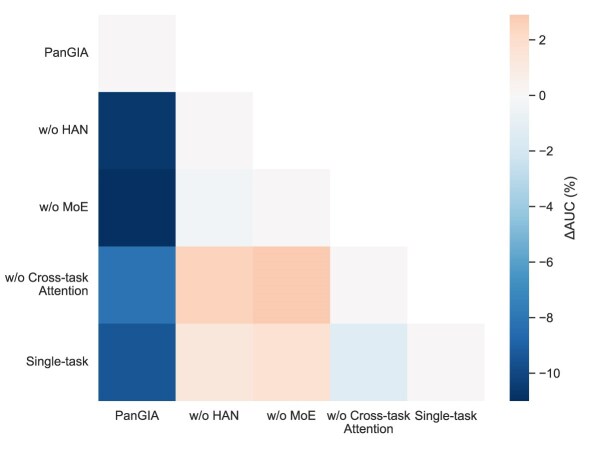
Performance comparison of PanGIA ablation variants on AUC.

**Figure 5:**
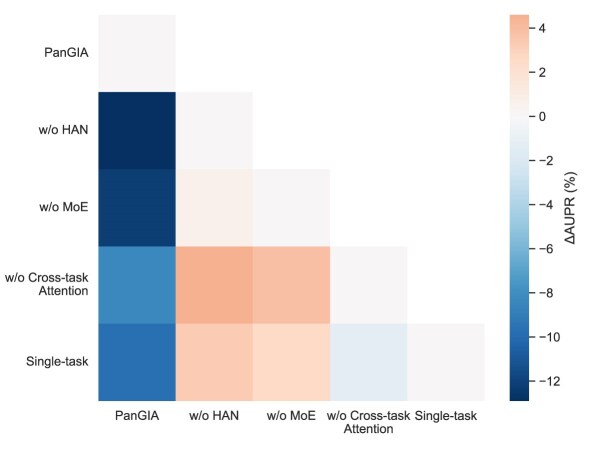
Performance comparison of PanGIA ablation variants on AUPR.

**Figure 6:**
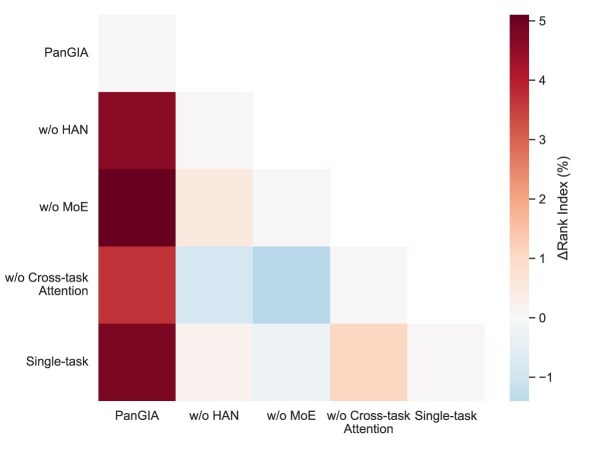
Performance comparison of PanGIA ablation variants on Rank Index.

To validate the effectiveness of key components in the PanGIA framework, we performed a series of ablation experiments by systematically removing core modules, including HAN, MoE, and the cross-task attention mechanism. Additionally, a single-task learning variant was tested to contrast against the full multitask framework. As illustrated in Fig. 4, Fig. 5 and Fig. 6, performance metrics including AUC, AUPR, and Rank Index were measured for each ablation variant.

The results demonstrate that removing any of the core components leads to a noticeable performance decline across all metrics. Specifically, the removal of HAN or MoE resulted in significant drops in both AUC and AUPR, indicating the importance of structural and expert-based representation learning. Furthermore, disabling the cross-task attention mechanism impaired the model’s ability to integrate information across tasks, reducing prediction accuracy. The single-task baseline also underperformed compared to the full model, highlighting the advantage of PanGIA’s multitask learning design. Overall, these findings confirm that each module contributes uniquely and substantially to the overall predictive power of PanGIA.

### PanGIA reveals novel ncRNA–disease associations

In the case study, we selected high-confidence associations between various types of ncRNAs (miRNA, circRNA, lncRNA, and piRNA) and representative diseases as predicted by the PanGIA model. These predicted associations were organized and presented in Table 2, respectively. Through literature review, we confirmed that all the associations listed in the table have been experimentally validated, with supporting evidence provided by the corresponding references (PMIDs).

**Table 2.** Experimentally validated ncRNA–disease associations used in the case study

RNA type	RNA symbol	Disease name	PMID
miRNA	miR-944	Glioblastoma	34233294
miRNA	miR-936	Glioblastoma	29218238
miRNA	miR-378	Osteoarthritis	35474736
miRNA	miR-139	Osteoarthritis	32185303
circRNA	CSPP1	Glioblastoma	32495924
circRNA	SCN3B	Glioblastoma	39289188
circRNA	ROCK1	Coronary artery disease	34236817
circRNA	WNK1	Coronary artery disease	31821324
lncRNA	LINC00324	Stomach carcinoma	32855634
lncRNA	LINC00691	Stomach carcinoma	32330554
lncRNA	RPSAP52	Stomach carcinoma	35322746
lncRNA	AFAP1-AS1	Cholangiocarcinoma	28938565
piRNA	DQ570326	Parkinson’s disease	29986767
piRNA	DQ592957	Parkinson’s disease	29986767
piRNA	DQ596377	Alzheimer’s disease	28127595
piRNA	DQ597397	Renal cell carcinoma	25998508

All associations were experimentally validated and supported by the referenced PubMed IDs (PMIDs).

In this case study, we focused on high-confidence miRNA–disease associations predicted by our model. Literature searches confirmed that these miRNAs are supported by clear biological mechanisms. For example, miR-944 derived from glioma stem cell exosomes directly downregulates VEGFC expression, further suppressing AKT/ERK signaling activity, thereby significantly reducing glioblastoma growth and angiogenesis [47]. Similarly, miR-936 is markedly downregulated in glioblastoma tissues, with its expression negatively correlated with tumor grade. Re-expression of miR-936 can target the CKS1 gene and inhibit the downstream AKT/ERK pathway, effectively blocking the cell cycle and suppressing tumor growth [48]. In osteoarthritis (OA), overexpression of miR-378 aggravates cartilage degeneration by suppressing autophagy in chondrocytes and inhibiting chondrogenic differentiation of bone marrow mesenchymal stem cells. Its targets, Atg2a and Sox6, are well characterized; conversely, the application of anti–miR-378 alleviates OA progression and promotes joint regeneration, highlighting its therapeutic potential [49]. In addition, miR-139 is significantly upregulated in OA-damaged cartilage and can be activated by IL-1$\beta$. By directly targeting MCPIP1, it relieves translational repression of IL-6, leading to elevated IL-6 and degradative enzymes such as MMP-13 and ADAMTS4, thereby promoting chondrocyte apoptosis and matrix degradation [50].

Regarding circRNAs, multiple experimental findings also support the model predictions. CSPP1 is markedly upregulated in glioblastoma tissues, closely associated with abnormal mitosis and the proliferation of tumor cells [51]. Similarly, SCN3B has been identified as a key molecule in glioblastoma, with aberrant expression linked to enhanced tumor cell migration and invasion [52]. In cardiovascular disease, ROCK1-related circRNA plays an important role in coronary artery disease by regulating vascular smooth muscle cell contraction and apoptosis, thereby promoting disease progression [53]. Meanwhile, WNK1-derived circRNA is significantly upregulated in patients with coronary artery disease, affecting endothelial function and ion channel homeostasis, thus accelerating atherosclerosis development [54]. These results further demonstrate the molecular significance of circRNAs in diverse diseases, validating the reliability and biological value of our model predictions.

For lncRNAs, several experimentally validated findings support the predicted associations. LINC00324 is significantly downregulated in stomach carcinoma, where it interacts with miR-3200-5p to regulate downstream BCAT1 expression, thereby inhibiting tumorigenesis [55]. Similarly, LINC00691 is upregulated in gastric cancer tissues and promotes proliferation and invasion by modulating the miR-9-5p/FGFR1 axis, suggesting its oncogenic role [56]. Furthermore, RPSAP52 enhances proliferation and inhibits apoptosis in gastric cancer by regulating the miR-665/STAT3 pathway, thus promoting tumor progression [57]. In cholangiocarcinoma, AFAP1-AS1 is markedly upregulated and promotes migration and invasion through transcriptional regulation of EMT (epithelial-mesenchymal transition)-related genes, underscoring its pivotal role in tumor progression [58].

For piRNAs, most predicted associations have been experimentally verified to show differential expression and participation in pathological processes. For instance, DQ597397 is significantly upregulated in renal cell carcinoma cells compared with normal renal cells, suggesting its role in promoting tumor progression [59]. Conversely, DQ570326 and DQ592957 are downregulated in neurons derived from patients with Parkinson’s disease, potentially contributing to neurodegenerative mechanisms [60]. Moreover, DQ596377 is markedly upregulated in neurons from patients with Alzheimer’s disease (AD), with expression levels 11.38-fold higher than those in normal brain cells, indicating its involvement in AD-specific neuropathological processes [61]. Collectively, these findings demonstrate that piRNAs play crucial molecular roles in the pathogenesis of multiple major diseases and further substantiate the reliability of our model predictions.

This result indicates that PanGIA performs excellently in the aforementioned case studies, demonstrating its capability to identify high-confidence associations between miRNAs, circRNAs, lncRNAs, and piRNAs with diseases. The unconfirmed associations predicted by PanGIA may serve as candidate targets for subsequent biological experiments and lay a solid foundation for the potential application of related ncRNAs in disease diagnosis and therapy.

## Conclusions

In this study, we proposed **PanGIA**, a novel model for ncRNA–disease association prediction that integrates HAN with a MoE architecture. Comprehensive experiments demonstrate that PanGIA achieves consistently superior performance across various RNA types, including miRNA, lncRNA, circRNA, and piRNA, validating the effectiveness of our multisource feature extraction and multitask modeling strategy.

PanGIA not only outperforms state-of-the-art methods on multiple evaluation metrics but also maintains robust and stable performance across different ncRNA categories. This indicates strong generalization and robustness of the model in handling heterogeneous RNA data and structures.

Through further case study analyses, we validated that several high-confidence predictions have been experimentally confirmed in the literature, highlighting the significant advantages of this method in terms of biological interpretability and result reliability. In particular, the ncRNA–disease associations predicted by PanGIA show substantial research and application value in fields such as neurological disorders, metabolic diseases, and cancer.

Overall, PanGIA demonstrates strong potential as a unified framework for pan-ncRNA–disease association prediction. It excels in both macro-level performance benchmarks and micro-level case reliability, suggesting excellent cross-task generalizability and interpretability. In future work, incorporating additional omics data and optimizing the network architecture may further enhance its predictive power, contributing to ncRNA functional studies, disease mechanism exploration, and the advancement of precision medicine and biomarker discovery.

## Supplementary Material

giaf123_Authors_Response_To_Reviewer_Comments_Original_Submission

giaf123_GIGA-D-25-00208_Original_Submission

giaf123_GIGA-D-25-00208_Revision_1

giaf123_Reviewer_1_Report_Original_Submission Veronica Buttaro -- 7/9/2025

giaf123_Reviewer_1_Report_Revision_1 Veronica Buttaro -- 9/1/2025

giaf123_Reviewer_2_Report_Original_Submission Wei Lan -- 7/14/2025

giaf123_Reviewer_2_Report_Revision_1 Wei Lan -- 8/27/2025

## Data Availability

The supporting data underlying this study are available in the *GigaScience* Database, GigaDB [62].

## Availability of Source Code and Requirements

- Project name: PanGIA
- Project homepage: https://github.com/qiankunzizairen/PanGIA
- License: MIT license
- Operating system: Linux
- Programming language: Python
- Package management: pip
- Hardware requirements: Standard workstation with at least 20 GB memory; GPU recommended for training with more than 20 GB memory.
- SciCrunch RRID: SCR_027069
- bio.tools ID: rna-disease-predictor
- DOME-DL: https://registry.dome-ml.org/review/yi6cmalcqe

## Abbreviations

AD: Alzheimer’s disease; AUC: area under the curve; AUPR: area under the precision-recall curve; ceRNA: competitive endogenous RNA; circRNA: circular RNA; DOID: Disease Ontology Identifiers; GIP: Gaussian Interaction Profile; HAN: heterogeneous graph attention network; lncRNA: long noncoding RNA; miRNA: microRNA; MLM: masked language model; MoE: mixture-of-experts; ncRNA: noncoding RNA; OA: osteoarthritis; PanGIA: Pan-ncRNA Graph-Interaction Attention network; piRNA: PIWI-interacting RNA.
