# Supplementary material for: PanGIA: A universal framework for identifying association between ncRNAs and diseases
Source: Gigascience. 2025 Oct 17;14:giaf123. doi: 10.1093/gigascience/giaf123 (PMC12532321; doi:10.1093/gigascience/giaf123)
Supplement: giaf123_GIGA-D-25-00208_Original_Submission [file giaf123_giga-d-25-00208_original_submission.pdf]

GigaScience
PanGIA: A universal framework for identifying association between ncRNAs and diseases
--Manuscript Draft--

**Manuscript Number:**	GIGA-D-25-00208
**Full Title:**	PanGIA: A universal framework for identifying association between ncRNAs and diseases
**Article Type:**	Research
**Funding Information:**	Heilongjiang Province Basic Research Support Program (YQJH2023195) — Mr. Xiaoyuan Liu
**Abstract:**	With the increasing recognition of the crucial roles that non-coding RNAs (ncRNAs) play in various biological processes—particularly their potential involvement in numerous human diseases—the effective prediction of ncRNA–disease associations has become a critical issue in biomedical research. Although numerous computational methods have been proposed to predict associations between non-coding RNAs (ncRNAs) and diseases, most of these approaches focus exclusively on a single class of ncRNAs. However, competitive and cooperative interactions among different types of ncRNAs are closely related to their functional roles in disease associations. To address this limitation, we propose a novel computational framework, PanGIA (Pan-ncRNA Graph-Interaction Attention network), which is designed to simultaneously predict potential associations between multiple types of non-coding RNAs—including miRNA, lncRNA, circRNA, and piRNA—and diseases. This method innovatively integrates the Heterogeneous Graph Attention Network (HAN) with the Mixture of Experts (MoE) framework. By constructing a unified cross-modal heterogeneous graph, it effectively incorporates diverse data sources, including ncRNA sequence features, functional annotations, and interaction networks between ncRNAs and diseases. Experiments show that PanGIA achieves higher accuracy than typespecific models in both individual and comprehensive predictions. It remains robust under node or ncRNA-type removal, and ablation studies confirm the benefit of cross-type information. PanGIA improves AUC and AUPR by up to 7.1% and 6.5%, respectively, over single-type sota methods. Case studies on top-ranked predictions further validate the model's biological relevance with prior experimental evidence. In particular, it shows significant advantages in predicting disease associations for different types of ncRNA, including miRNA, lncRNA, circRNA, and piRNA. The case studies further validated the accuracy of the model's predictions, as all high-confidence associations were supported by literature evidence. This demonstrates the model's strong biological interpretability and promising potential for practical applications. The successful application of PanGIA offers a new paradigm for exploring disease-associated ncRNAs, highlighting their great potential in the field of biomedical research.
**Corresponding Author:**	Tianyi Zhao<br>Harbin Institute of Technology<br>harbin, CHINA
**Corresponding Author Secondary Information:**	
**Corresponding Author's Institution:**	Harbin Institute of Technology
**Corresponding Author's Secondary Institution:**	
**First Author:**	Xiaoyuan Liu
**First Author Secondary Information:**	
**Order of Authors:**	Xiaoyuan Liu
	Xiye Lü
	Qiuhao Chen

Powered by Editorial Manager® and ProduXion Manager® from Aries Systems Corporation

	Tianyi Zhao
	Yan Zhu
**Order of Authors Secondary Information:**	
**Additional Information:**	
**Question**	**Response**
Are you submitting this manuscript to a special series or article collection?	No
**Experimental design and statistics**<br><br>Full details of the experimental design and statistical methods used should be given in the Methods section, as detailed in our Minimum Standards Reporting Checklist. Information essential to interpreting the data presented should be made available in the figure legends.<br><br>Have you included all the information requested in your manuscript?	Yes
**Resources**<br><br>A description of all resources used, including antibodies, cell lines, animals and software tools, with enough information to allow them to be uniquely identified, should be included in the Methods section. Authors are strongly encouraged to cite Research Resource Identifiers (RRIDs) for antibodies, model organisms and tools, where possible.<br><br>Have you included the information requested as detailed in our Minimum Standards Reporting Checklist?	Yes
**Availability of data and materials**<br><br>All datasets and code on which the conclusions of the paper rely must be either included in your submission or deposited in publicly available repositories (where available and ethically appropriate), referencing such data using	Yes

a unique identifier in the references and in the “Availability of Data and Materials” section of your manuscript.<br><br>Have you have met the above requirement as detailed in our Minimum Standards Reporting Checklist?	
GigaScience has policies and guidelines in place for the use of generative AI-writing tools such as ChatGPT. If you have used such writing tools to assist with writing the manuscript this must be declared and cited in the text. Authors should not list AI-writing tools and other AI-assisted technologies as an author or co-author and should acknowledge that they are fully responsible for text generated or refined by AI-writing tools.<p><br><br>A summary of use (particularly in the introduction or among methods) needs to be included at the end of the paper, and the outputs should also be included as a supplementary file hosted in GigaDB or other open repositories. Please read our guidelines for more information. <p><br><br>By submitting to GigaScience, you are aware of the journal's AI-writing tools policy, and if you have declared use of such tools below, you have acknowledged this where appropriate in your manuscript and have made a summary of use and outputs available. <p><br><b>AI-assisted writing tools have been used in the preparation of this manuscript?	Yes

Powered by Editorial Manager® and ProduXion Manager® from Aries Systems Corporation

Manuscript Click here to access/download;Manuscript;PanGIA2.pdf

Journal Title Here, 2022, pp. 1–12

doi: DOI HERE
Advance Access Publication Date: Day Month Year
Paper

PAPER

PanGIA: A universal framework for identifying association between ncRNAs and diseases

Xiaoyuan Liu,[1,†] Xiye Lü,[1,†] Qiuhao Chen,[2] Tianyi Zhao[1,2,*] and Yan Zhu[3,*]

[1]School of Medicine and Health, Harbin Institute of Technology, Xidazhi Street No.90, Nangang District, 150000, Harbin, China ,
[2]Zhengzhou Research Institute, Harbin Institute of Technology, Xidazhi Street No.90, Nangang District, 150000, Harbin, Heilongjiang, China and [3]College of Veterinary Medicine, Northeast Agricultural University, 150038, Harbin, Heilongjiang, China
[*]Corresponding authors. Tianyi Zhao. E-mail: zty2009@hit.edu.cn; Yan Zhu. E-mail: zhuyan8285@sina.com
FOR PUBLISHER ONLY Received on Date Month Year; revised on Date Month Year; accepted on Date Month Year

Abstract

With the increasing recognition of the crucial roles that non-coding RNAs (ncRNAs) play in various biological processesparticularly their potential involvement in numerous human diseasesthe effective prediction of ncRNAdisease associations has become a critical issue in biomedical research. Although numerous computational methods have been proposed to predict associations between non-coding RNAs (ncRNAs) and diseases, most of these approaches focus exclusively on a single class of ncRNAs. However, competitive and cooperative interactions among different types of ncRNAs are closely related to their functional roles in disease associations. To address this limitation, we propose a novel computational framework, **PanGIA** (Pan-ncRNA Graph-Interaction Attention network), which is designed to simultaneously predict potential associations between multiple types of non-coding RNAsincluding miRNA, lncRNA, circRNA, and piRNAand diseases. This method innovatively integrates the Heterogeneous Graph Attention Network (HAN) with the Mixture of Experts (MoE) framework. By constructing a unified cross-modal heterogeneous graph, it effectively incorporates diverse data sources, including ncRNA sequence features, functional annotations, and interaction networks between ncRNAs and diseases. Experiments show that PanGIA achieves higher accuracy than type-specific models in both individual and comprehensive predictions. It remains robust under node or ncRNA-type removal, and ablation studies confirm the benefit of cross-type information. PanGIA improves AUC and AUPR by up to 7.1% and 6.5%, respectively, over single-type sota methods. Case studies on top-ranked predictions further validate the model's biological relevance with prior experimental evidence. In particular, it shows significant advantages in predicting disease associations for different types of ncRNA, including miRNA, lncRNA, circRNA, and piRNA. The case studies further validated the accuracy of the models predictions, as all high-confidence associations were supported by literature evidence. This demonstrates the models strong biological interpretability and promising potential for practical applications. The successful application of PanGIA offers a new paradigm for exploring disease-associated ncRNAs, highlighting their great potential in the field of biomedical research.

Key words: Heterogeneous Graph Attention Network, Mixture-of-Experts, Cross-task Attention Mechanism, ncRNADisease Association

Introduction

Non-coding RNAs (ncRNAs) refer to a class of RNA molecules that do not encode proteins but play crucial roles in various biological processes, such as post-transcriptional regulation, epigenetic modification, and cellular signaling. In recent years, with the advancement of high-throughput sequencing technologies and functional genomics, an increasing number of ncRNAs have been identified as closely associated with a wide range of complex human diseases [1, 2, 3, 4]. A growing body of experimental evidence has demonstrated that aberrant expression or dysfunction of ncRNAs is involved in the pathogenesis of major diseases, including cancer, neurodegenerative disorders, and cardiovascular diseases. Therefore, uncovering the potential associations between ncRNAs and diseases not only helps to elucidate the molecular mechanisms underlying complex diseases, but also provides theoretical support for early diagnosis, biomarker discovery, and personalized treatment strategies. In particular, ncRNA regulatory mechanisms have emerged as a research hotspot in fields such as oncology, neurological disorders, and cardiovascular disease.

Among the various types of ncRNAs, small RNAs such as microRNAs (miRNAs) have been extensively studied and are well-recognized for their post-transcriptional silencing functions through binding to target mRNAs. They have demonstrated

© The Author 2022. Published by Oxford University Press. All rights reserved. For permissions, please e-mail: journals.permissions@oup.com

significant potential as biomarkers in a wide range of diseases [5, 6].

Circular RNAs (circRNAs), owing to their covalently closed-loop structures that confer high stability, can function as competitive endogenous RNAs (ceRNAs) for microRNAs (miRNAs) or interact with RNA-binding proteins. Increasing evidence has demonstrated that circRNAs play critical regulatory roles and possess considerable potential for clinical applications across various disease contexts [7, 8].

Long ncRNAs (lncRNAs), which function by interacting with DNA, RNA, or proteins, are involved in processes such as chromatin modification and transcriptional regulation, and have been found to play crucial roles in tumorigenesis, cell proliferation, and immune modulation [9, 10].

PIWI-interacting RNAs (piRNAs), initially thought to function predominantly in germ cells by suppressing transposable elements to maintain genome stability, have more recently been shown to exert regulatory functions in somatic cells as well. These piRNAs are increasingly associated with various cancers and metabolic disorders [11, 12].

Despite their critical roles in gene regulation and disease mechanisms, experimental identification of non-coding RNA (ncRNA)disease associations remains costly and time-consuming, limiting its scalability for large-scale studies. As a result, computational methods have gained increasing attention for their ability to efficiently and cost-effectively predict ncRNAdisease associations.

For miRNAs, representative approaches such as IMCMDA [13] utilize integrated similarity networks and bi-directional diffusion models to infer diseasemiRNA associations. In the lncRNA domain, LncDisAP [14] combines multiple similarity measures with deep representation learning to uncover latent associations. For piRNAs, IPiDA-GBNN [15] leverages graph neural networks and multi-feature embeddings to improve prediction accuracy. In the case of circRNAs, GCNCDA [16] constructs a heterogeneous graph and applies graph convolutional networks to learn potential circRNAdisease interactions.

Although these methods have achieved promising results within their respective ncRNA categories, they typically focus on a single type of ncRNA, ignoring the complex interplay and competition among different ncRNAs. For instance, miRNAs may interact with lncRNAs or circRNAs through the competing endogenous RNA (ceRNA) mechanism, jointly regulating disease-related pathways. These interactions form a complex regulatory network, yet existing approaches lack comprehensive modeling of both cross-ncRNA relationships and multi-type ncRNAdisease associations.

Therefore, there is a pressing need for novel computational frameworks that can jointly model the interrelations among various ncRNA types and their associations with diseases, enabling the discovery of previously unknown ncRNAdisease links through a more holistic understanding of their regulatory dynamics.

Despite the availability of several specialized repositories, such as miR2Disease, circR2Disease, LncRNADisease, and piRDisease, which systematically curate associations between non-coding RNAs (ncRNAs) and human diseases [17, 18, 19, 20], these databases are inherently constrained by their reliance on experimentally derived evidence. The acquisition of such evidence is both resource-intensive and time-consuming, with its breadth inherently limited by laboratory conditions and prevailing research focuses. Moreover, most current studies are restricted to individual ncRNA classes, thereby neglecting potential crosstalk and cooperative interactions among distinct ncRNA species in disease pathogenesis. Consequently, existing resources remain insufficient to fully capture the complexity of ncRNA-mediated regulatory networks.

In conventional studies, most computational approaches focus on a single type of non-coding RNA (ncRNA), such as miRNA, circRNA, lncRNA, or piRNA, and are typically tailored to the specific features and data types associated with that category. Examples include IMCMDA [13], LncDisAP [14], IPiDA-GBNN [15], and GCNCDA [16], among others. These models are generally built upon sequence information, structural properties, or expression profiles unique to the targeted ncRNA type. However, such type-specific methods exhibit clear limitations in their generalizability, as they are often not applicable to other classes of ncRNAs.

This limitation arises from the substantial differences in structure, biological function, and disease-related mechanisms among various ncRNA types. For instance, miRNAs primarily function through post-transcriptional repression by targeting mRNAs, whereas lncRNAs are involved in gene regulation and chromatin remodeling. CircRNAs are known to act as ”sponges” for miRNAs, and piRNAs are mainly implicated in post-transcriptional regulation and transposon silencing. Consequently, models focusing exclusively on one ncRNA type tend to ignore the potential interactions and synergies among different ncRNA categories, thereby restricting their applicability and limiting their potential to uncover cross-type regulatory mechanisms in disease contexts.

Furthermore, single-type RNA-based approaches are inadequate in capturing the complex biological interactions that may exist across different RNA types. For example, miRNAs may indirectly influence disease development by regulating lncRNA expression; circRNAs may impact disease progression through interactions with miRNAs; and piRNAs may engage with other RNA species in various biological processes. Since traditional methods are confined to individual RNA types, they are unable to fully elucidate the potential cross-talk and co-regulatory mechanisms among diverse ncRNA classes.

Therefore, there is an urgent need to develop an efficient and scalable computational prediction model capable of systematically identifying potential associations between different types of ncRNAs and diseases. Such a model would not only compensate for the limitations of experimental data but also expand the knowledge graph of disease regulatory networks.

In this paper, we propose **PanGIA** (Pan-ncRNA Graph-Interaction Attention network), a novel framework for comprehensive ncRNAdisease association prediction. To address the challenge of feature heterogeneity, PanGIA constructs a heterogeneous graph that integrates multi-source data, including sequence information, functional similarity, and interaction networks of ncRNAs. To capture comprehensive and layered associations, it employs a cross-task attention mechanism combined with a Mixture-of-Experts architecture to dynamically learn the multi-level interactions between ncRNAs and diseases. Notably, PanGIA encompasses four representative classes of non-coding RNAsmiRNAs, lncRNAs, circRNAs, and piRNAswhich exhibit distinct characteristics in terms of length, structure, regulatory mechanisms, and functional roles. These classes, owing to their complementary and representative nature in current research, are collectively referred to as pan-ncRNAs [21]. By adopting a pan-ncRNA perspective, PanGIA not only overcomes the limitations of single-type ncRNA studies

but also provides a more comprehensive understanding of the multilayered regulatory roles of ncRNAs in disease.

The main contributions of our work are summarized as follows:

- **Pan-ncRNA integration:** PanGIA jointly models four types of ncRNAs (miRNA, lncRNA, circRNA, and piRNA), overcoming the limitations of single-type approaches.
- **Heterogeneous graph fusion:** It constructs a heterogeneous graph to integrate sequence, semantic, and functional data, capturing complex ncRNAdisease relationships.
- **Cross-task attention:** A Mixture-of-Experts module with cross-task attention enhances feature sharing across ncRNA types and improves prediction accuracy.
- **Superior performance:** PanGIA achieves higher AUC, AUPR, and rank metrics than baseline models, demonstrating strong generalization and reliability.

Materials

Our study involves multiple classes of non-coding RNAs and requires the simultaneous acquisition of their sequence information and disease association data. The databases utilized in this study are listed as follows:

- **miRNA**:The associations between miRNAs and diseases were obtained from the HMDD v4.0 database [22], while the sequence information of miRNAs was retrieved from the miRBase database [23].
- **LncRNA/circRNA:** This study includes lncRNA and circRNA associations with diseases, with data obtained from LncRNADisease v3.0 [24]. The sequence information of circRNAs was retrieved from the circBase database [25]. In contrast, lncRNA sequences were collected from two sources: GENCODE [26] and NONCODE [27].
- **piRNA**:The associations between piRNAs and diseases were obtained from the piRDisease v1.0 [19] database, and the sequence information was retrieved from the piRBase [28] and piRNAdb [29] databases.
- **Disease:** This study utilizes Disease Ontology Identifiers (DOIDs) to construct the disease similarity matrix, with corresponding information obtained from the Disease Ontology database [30].

The construction of the ncRNAdisease association network was based on merging data entries from the aforementioned association databases.

Methods

We propose a novel model named **PanGIA** (Pan-ncRNA Graph-Interaction Attention network), which is built upon the Heterogeneous Graph Attention Network (HAN) and a Mixture of Experts (MoE) framework. The model is designed to predict associations between pan-ncRNAs and diseases. The overall workflow of **PanGIA** is illustrated in Figure 1, and consists of three main steps:

1. **Pretraining:** Embedding Representation of ncRNA Nodes.
2. **Data Processing:** Generation of Heterogeneous Networks
3. **Model Construction:** Multi-Task Association Prediction via HAN and Mixture-of-Experts with Cross-Task Attention

Pretraining: Embedding Representation of ncRNA Nodes

3-mer Tokenization and Embedding Representation

We begin by sliding a fixed-length window to generate 3-mer subsequences from the RNA sequences. Notably, the 3-mer count distributions differ significantly across the four ncRNA types: miRNA and piRNA exhibit low, concentrated counts, while circRNA and lncRNA display much higher and more dispersed distributions. In particular, circRNA shows extremely high mean and standard deviation values, indicating a heavy-tailed distribution. This long-tail characteristic poses challenges for encoding and may introduce bias in model training.

Given these heterogeneous distributions, using the global maximum count as a uniform encoding boundary is suboptimal, leading to substantial space inefficiency and degraded model performance. Although truncation is a common strategy for handling long-tail sequence data, it is unsuitable in this context. Numerous studies have highlighted the importance of long-read sequencing in capturing ncRNAdisease associations, making preservation of full-length sequences crucial.

To address the challenges posed by long-read sequencing data, we introduce a sliding random sampling strategy. Specifically, the 95th percentile of the 3-mer counts across all pan-ncRNAs is selected as the threshold. Based on this threshold, the ncRNA embedding encoding is divided into two cases:

For ncRNAs whose 3-mer counts do not exceed the specified threshold, the sequence S of length n is segmented into 3-mers using the following procedure:

$$K(S,k) = \{S[i:i+k] \mid i \in \{0,1,\ldots,n-k\}\} \tag{1}$$

$S[i:i+k]$ denotes the continuous substring of length k starting from the i-th position of the sequence S. The segmentation results in a set containing all possible k-mers generated from the sequence.

For ncRNA sequences with 3-mer counts exceeding the predefined threshold, a sliding random sampling strategy is applied using a window size w and stride s. The sampling result is formally defined as:

$$R(S,w,s) = \{S[i:i+w] \mid i \in \{0,s,2s,\ldots,n-w\}\} \tag{2}$$

If the window cannot completely cover the end of the sequence, an additional window is appended to ensure full coverage:

$$R(S,w,s) = R(S,w,s) \cup \{S[n-w:n]\} \tag{3}$$

In this study, we employ a Word2Vec-based embedding approach to characterize four types of non-coding RNAs (miRNA, circRNA, lncRNA, and piRNA). By segmenting each RNA sequence into fixed-length k-mers and constructing the corresponding training corpus, the Word2Vec model captures the contextual relationships among k-mers and generates high-quality continuous vector embeddings [31, 32]. This method not only effectively compresses the high-dimensional sequence information, but also preserves the biological semantics of the original sequences. As a result, it provides a unified and biologically interpretable feature representation for subsequent machine learning models.

Through the above process, ncRNAs with 3-mer counts below the threshold are represented as sequence-level information, whereas long-read ncRNAs are encoded as segment-level information [33].

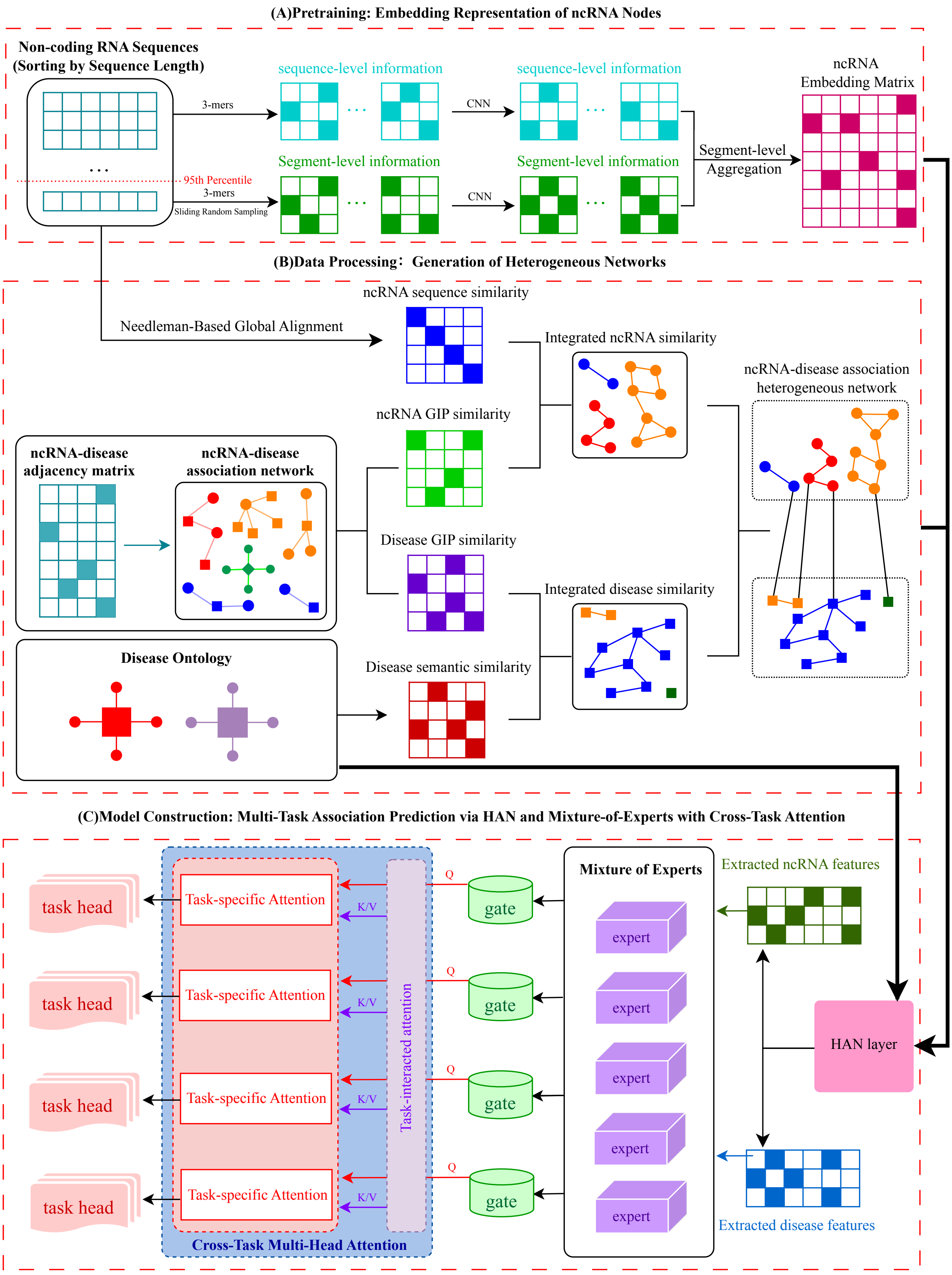

Fig. 1. The structure of PanGIA.

Feature Extraction and Aggregation

To effectively extract high-level features from RNA sequences, we design and implement a deep learning-based feature extraction module. This model takes the pretrained embedding vectors as input and applies multi-scale convolutional operations to capture contextual information across different receptive fields. An adaptive pooling strategy is incorporated to normalize sequence features and generate fixed-dimensional representations. Through this approach, the model not only captures local patterns within the sequences, but also produces high-quality, uniform feature vectors that serve as robust inputs for downstream tasks.

Subsequently, a segment-level feature aggregation strategy is employed. Specifically, for each RNA sequence, the feature vectors of all its corresponding segments are accumulated according to their sequence indices. The average of the accumulated feature vectors is then computed to generate the global feature representation for each sequence. This aggregation strategy effectively preserves local information at the segment level, while the average pooling ensures global consistency of the feature representation. As a result, the variable-length segment features are transformed into standardized sequence-level features, providing a unified input format for subsequent analyses.

Data Processing:Generation of Heterogeneous Networks

In this study, we first processed the raw non-coding RNA (ncRNA) sequence data and their known associations with diseases to construct a cross-modal heterogeneous network, which serves as the input foundation for the PanGIA model. The constructed heterogeneous network is presented as follows:

$$\mathcal{N}_{\mathrm{H}} = \begin{bmatrix} \mathbf{S}_{\mathrm{RNA}} & \mathbf{A} \\ \mathbf{A}^{\top} & \mathbf{S}_{\mathrm{di}} \end{bmatrix} \tag{4}$$

$\mathcal{N}_{\mathrm{H}}$ denotes the adjacency matrix of the constructed heterogeneous network; $\mathbf{S}_{\mathrm{RNA}}$ represents the similarity matrix between ncRNA nodes; $\mathbf{S}_{\mathrm{di}}$ denotes the similarity matrix between disease nodes; and $\mathbf{A}$ refers to the known association adjacency matrix between ncRNAs and diseases.

Construction of Non-coding RNA Similarity Matrices

In this study, we first focused on four major classes of ncRNA sequencesmiRNA, lncRNA, circRNA, and piRNA. For each ncRNA class, we calculated the similarity between any two sequences using the global sequence alignment method based on the NeedlemanWunsch algorithm, thereby constructing the corresponding sequence similarity matrices. The formula for the NeedlemanWunsch alignment algorithm is as follows:

$$S_{ij}^{\mathrm{seq}} = \frac{\mathrm{score}(x_i, x_j)}{\max\left(\mathrm{score}(x_i, x_i), \mathrm{score}(x_j, x_j)\right)} \tag{5}$$

x_i and x_j denote any two sequences of the same type of ncRNA, and score represents the alignment scoring function. The resulting similarity matrix is normalized to ensure that all values fall within the range $[0, 1]$.

The resulting sequence similarity matrices for the non-coding RNAs are structured as follows:

$$\mathbf{S}_{\mathrm{RNA}}^{\mathrm{seq}} = \begin{bmatrix} \mathbf{S}_{\mathrm{mi}}^{\mathrm{seq}} & \mathbf{0} & \mathbf{0} & \mathbf{0} \\ \mathbf{0} & \mathbf{S}_{\mathrm{circ}}^{\mathrm{seq}} & \mathbf{0} & \mathbf{0} \\ \mathbf{0} & \mathbf{0} & \mathbf{S}_{\mathrm{lnc}}^{\mathrm{seq}} & \mathbf{0} \\ \mathbf{0} & \mathbf{0} & \mathbf{0} & \mathbf{S}_{\mathrm{pi}}^{\mathrm{seq}} \end{bmatrix} \tag{6}$$

$\mathbf{S}_{\mathrm{mi}}^{\mathrm{seq}}$, $\mathbf{S}_{\mathrm{circ}}^{\mathrm{seq}}$, $\mathbf{S}_{\mathrm{lnc}}^{\mathrm{seq}}$, and $\mathbf{S}_{\mathrm{pi}}^{\mathrm{seq}}$ represent the sequence similarity matrices of miRNA, circRNA, lncRNA, and piRNA, respectively.

Based on the constructed ncRNAdisease association network, we next compute the functional similarity of non-coding RNAs using the Gaussian Interaction Profile (GIP) kernel function. The corresponding formula is given as follows:

$$\mathbf{S}_{\mathrm{RNA}}^{\mathrm{GIP}}(r_i, r_j) = \exp\left(-\lambda_{\mathrm{RNA}} \left\|\mathbf{A}(r_i, :) - \mathbf{A}(r_j, :)\right\|^2\right) \tag{7}$$

In this formulation, $\mathbf{A}(r_i, :)$ and $\mathbf{A}(r_j, :)$ represent the vectors corresponding to the i-th and j-th rows of the adjacency matrix $\mathbf{A}$, respectively. The parameter λ_{RNA} denotes the bandwidth coefficient of the kernel function, which is defined as follows:

$$\lambda_{\mathrm{RNA}} = \frac{1}{\frac{1}{N_{\mathrm{r}}} \sum_{k=1}^{N_{\mathrm{r}}} \left\|\mathbf{A}(r_k, :)\right\|^2} \tag{8}$$

N_r denotes the total number of ncRNAs, and $\mathbf{A}(r_k, :)$ represents the vector corresponding to the k-th row of the adjacency matrix $\mathbf{A}$. Subsequently, we integrate the sequence similarity and GIP-based functional similarity to obtain the final ncRNA similarity matrix:

$$\mathbf{S}_{\mathrm{RNA}} = \frac{\mathbf{S}_{\mathrm{RNA}}^{\mathrm{seq}} + \mathbf{S}_{\mathrm{RNA}}^{\mathrm{GIP}}}{2} \tag{9}$$

Construction of the Disease Similarity Matrix

On the disease side, we constructed two types of disease similarity networks based on different approaches: (i) a semantic similarity matrix calculated using Disease Ontology, and (ii) a GIP-based similarity matrix generated from disease interaction profiles. By integrating these two sources of similarity information, we obtained a comprehensive disease similarity network to enhance the accuracy of disease representation [34, 35, 36].

Disease Ontology is a structured ontology that organizes various diseases and their hierarchical relationships. Each disease node in the ontology is assigned a unique identifier and may be associated with descriptive attributes, such as symptoms and causes. The hierarchical structure of the ontology typically resembles a tree, where parent nodes represent broader disease categories and child nodes correspond to more specific diseases. In this study, we employ the Jaccard similarity coefficient to compute the semantic similarity matrix between diseases, defined as follows:

$$\mathbf{S}_{\mathrm{di}}^{\mathrm{sem}} = \frac{|\mathrm{Ancestors}(d_1) \cap \mathrm{Ancestors}(d_2)|}{|\mathrm{Ancestors}(d_1) \cup \mathrm{Ancestors}(d_2)|} \tag{10}$$

In this formulation, $\mathrm{Ancestors}(d)$ denotes the set of ancestor nodes of the disease node d, and $|A|$ represents the cardinality (i.e., the number of elements) of the set A.

The functional similarity of diseases based on the Gaussian Interaction Profile (GIP) kernel is calculated as follows:

$$\mathbf{S}_{\mathrm{di}}^{\mathrm{GIP}}(d_i, d_j) = \exp\left(-\lambda_{\mathrm{di}} \left\|\mathbf{A}(:, d_i) - \mathbf{A}(:, d_j)\right\|^2\right) \tag{11}$$

In this formulation, $\mathbf{A}(:, d_i)$ and $\mathbf{A}(:, d_j)$ represent the vectors corresponding to the i-th and j-th columns of the adjacency matrix $\mathbf{A}$, respectively. The parameter λ_{di} denotes the bandwidth coefficient of the kernel function, which is defined as follows:

$$\lambda_{\mathrm{d}} = \frac{1}{\frac{1}{N_{\mathrm{d}}} \sum_{k=1}^{N_{\mathrm{d}}} \left\|\mathbf{A}(:, d_k)\right\|^2} \tag{12}$$

N_d denotes the total number of diseases. Subsequently, we integrate the semantic similarity and GIP-based functional

similarity to obtain the final integrated disease similarity matrix:

$$\mathbf{S}_{\text{di}} = \frac{\mathbf{S}_{\text{di}}^{\text{sem}} + \mathbf{S}_{\text{di}}^{\text{GIP}}}{2} \tag{13}$$

We integrate the constructed ncRNA similarity network, the disease similarity network, and the known ncRNAdisease associations to form a unified ncRNAdisease heterogeneous graph network, denoted as $\mathcal{N}_{\text{H}}$.

Model Construction: Multi-Task Association Prediction via HAN and Mixture-of-Experts with Cross-Task Attention

We propose a novel multi-task relational prediction framework that integrates a Heterogeneous Graph Attention Network (HAN) with a Mixture-of-Experts (MoE) mechanism. Through a Cross-Task Attention mechanism, the framework enables collaborative modeling across tasks, enhancing both task generalization and interaction expression capabilities. The overall structure of the model is depicted in Figure 1(C), which consists of the following five main components:

Feature Representation and Heterogeneous Graph Modeling

The model input consists of the ncRNA embedding matrix $\mathbf{X}^{\text{rna}} \in \mathbb{R}^{N_r \times d_r}$ and the disease embedding matrix $\mathbf{X}^{\text{dis}} \in \mathbb{R}^{N_d \times d_d}$, where N_r and N_d represent the number of ncRNAs and diseases, respectively, and d_r and d_d correspond to their embedding dimensions. Since the original feature dimensions of ncRNAs and diseases may differ, we first map the disease embeddings into the same space as the ncRNA embeddings:

$$\mathbf{X}_{\text{proj}}^{\text{dis}} = \mathbf{X}^{\text{dis}}\mathbf{W}_d \in \mathbb{R}^{N_d \times d_r} \tag{14}$$

Where $\mathbf{W}_d \in \mathbb{R}^{d_d \times d_r}$ is a learnable linear transformation matrix.

Next, the heterogeneous network $\mathcal{N}_H$, based on ncRNA-disease associations, along with the ncRNA embedding matrix $\mathbf{X}^{\text{rna}}$ and disease embedding matrix $\mathbf{X}^{\text{dis}}$, is fed into the HAN model. A multi-head attention mechanism, guided by meta-paths, is employed to extract higher-order semantic features from the graph structure. The output of the HAN encoder is:

$$\mathbf{H}^{\text{rna}}, \mathbf{H}^{\text{dis}} = \text{HANEncoder}(\mathbf{X}^{\text{rna}}, \mathbf{X}_{\text{proj}}^{\text{dis}}, \mathcal{N}_H) \tag{15}$$

Where $\mathbf{H}^{\text{rna}}, \mathbf{H}^{\text{dis}} \in \mathbb{R}^{N \times d_h}$ represent the hidden representations of ncRNAs and diseases extracted by the HAN layer, and d_h denotes the intermediate hidden dimension.

Expert Pool and Global Disease Information Fusion

To integrate global disease semantics, we average the representations of all disease nodes to obtain the global disease feature:

$$\bar{\mathbf{H}}^{\text{dis}} = \frac{1}{N_d}\sum_{i=1}^{N_d} \mathbf{H}_i^{\text{dis}} \in \mathbb{R}^{d_h} \tag{16}$$

We concatenate this with each ncRNA representation to form the fused representation:

$$\mathbf{F} = \left[\mathbf{H}^{\text{rna}} \,\|\, \bar{\mathbf{H}}^{\text{dis}}\right] \in \mathbb{R}^{N_r \times 2d_h} \tag{17}$$

Subsequently, the fused representation is input into an expert pool consisting of K experts, where each expert is a nonlinear transformation module:

$$\mathcal{E}_k(\mathbf{F}) = \text{ReLU}(\mathbf{F}\mathbf{W}_k + \mathbf{b}_k), \quad k = 1, \ldots, K \tag{18}$$

The outputs of all experts are then stacked:

$$\mathbf{E} = \text{stack}(\mathcal{E}_1(\mathbf{F}), \ldots, \mathcal{E}_K(\mathbf{F})) \in \mathbb{R}^{N_r \times K \times d_e} \tag{19}$$

Where d_e denotes the output dimension of each expert.

Multi-Task Gating Mechanism

For each specific task $t \in \{1, \ldots, T\}$, the corresponding ncRNA subset is $\mathcal{I}_t \subset \{1, \ldots, N_r\}$. We learn a gating network for each task to perform attention-based selection of experts in the expert pool:

- First, the task-related input representation $\mathbf{H}_t = \mathbf{H}^{\text{rna}}[\mathcal{I}_t]$ is mapped to a query vector $\mathbf{Q}_t$;
- The expert representations are mapped to keys $\mathbf{K}_t$ and values $\mathbf{V}_t$;
- A multi-head attention mechanism is then used to compute the attention-weighted expert representation:

$$\mathbf{A}_t = \text{softmax}(\text{MultiHeadAttn}(\mathbf{Q}_t, \mathbf{K}_t, \mathbf{V}_t)) \in \mathbb{R}^{n_t \times K} \tag{20}$$

The final aggregation of the expert outputs results in the task feature representation:

$$\mathbf{u}_t = \sum_{k=1}^{K} \mathbf{A}_{t,:,k} \cdot \mathbf{E}_{\mathcal{I}_t,k} \in \mathbb{R}^{n_t \times d_e} \tag{21}$$

Cross-Task Attention Interaction

To model the potential correlations between tasks, we input the aggregated representations of all tasks (after average pooling) into a cross-task multi-head attention module:

$$\mathbf{U} = [\bar{\mathbf{u}}_1, \ldots, \bar{\mathbf{u}}_T] \in \mathbb{R}^{T \times d_e}, \quad \bar{\mathbf{u}}_t = \frac{1}{n_t}\sum_{i=1}^{n_t} \mathbf{u}_{t,i} \tag{22}$$

$$\mathbf{U}' = \text{MultiHeadAttn}(\mathbf{U}, \mathbf{U}, \mathbf{U}) \in \mathbb{R}^{T \times d_e} \tag{23}$$

The cross-task global representation $\mathbf{U}'_t$ is then concatenated with the original task representation $\mathbf{u}_t$:

$$\mathbf{z}_t = \text{ReLU}([\mathbf{u}_t \,\|\, \mathbf{U}'_t]) \in \mathbb{R}^{n_t \times d_e} \tag{24}$$

Relational Prediction and Output Layer

The representations of all diseases are projected to the expert dimension through a linear transformation:

$$\tilde{\mathbf{H}}^{\text{dis}} = \mathbf{H}^{\text{dis}}\mathbf{W}_{\text{proj}} \in \mathbb{R}^{N_d \times d_e} \tag{25}$$

Finally, the association score between each ncRNA and all diseases for each task is calculated through the dot product, followed by normalization using the sigmoid function:

$$\hat{\mathbf{Y}}_t = \sigma(\mathbf{z}_t \cdot \tilde{\mathbf{H}}^{\text{dis}^\top}) \in \mathbb{R}^{n_t \times N_d} \tag{26}$$

Results

benchmark on various ncRNAs

In this study, we evaluated the performance of different models using five-fold cross-validation and employed Rank Index, AUC

Table 1. Performance comparison of PanGIA and baseline models on ncRNAdisease association prediction tasks.

Model	RNA Category	AUC	Rank Index	AUPR
NIMGSA [37]	miRNA	0.544 0.006	0.281 0.007	0.093 0.007
MINIMDA [38]	miRNA	0.933 0.012	0.244 0.006	0.846 0.077
PanGIA	miRNA	0.923 0.009	0.206 0.005	0.847 0.007
gGATLDA [39]	lncRNA	0.921 0.021	0.303 0.003	0.937 0.025
MHRWR [40]	lncRNA	0.826 0.035	0.382 0.001	0.739 0.049
PanGIA	lncRNA	0.939 0.087	0.199 0.011	0.781 0.021
iPiDi-PUL [41]	piRNA	0.569 0.026	0.444 0.021	0.117 0.008
PUTransGCN [42]	piRNA	0.930 0.007	0.103 0.006	0.598 0.032
PanGIA	piRNA	0.942 0.001	0.235 0.004	0.741 0.005
IGNSCDA [43]	circRNA	0.890 0.011	0.326 0.014	0.599 0.053
GATCL2CD [44]	circRNA	0.880 0.021	0.228 0.006	0.732 0.002
PanGIA	circRNA	0.932 0.050	0.257 0.024	0.770 0.027
PanGIA	miRNA, lncRNA, piRNA, circRNA	0.953 0.001	0.192 0.001	0.839 0.008

(Area Under the Curve), and AUPR (Area Under the Precision-Recall Curve) as evaluation metrics.

Under the evaluation of five-fold cross-validation, the performance of various models on the pan-ncRNAdisease association prediction task is summarized in Table 1. Compared with the baseline methods, PanGIA consistently achieved the best performance across multiple evaluation metrics.

To comprehensively evaluate the performance of the PanGIA model in multi-type ncRNA-disease association prediction, this study conducted comparative experiments using various existing mainstream methods across four types of ncRNAsmiRNA, lncRNA, piRNA, and circRNA. The models' performance was assessed using AUC, AUPR, and Rank Index metrics. As shown in the table, PanGIA outperformed all other methods across all RNA types. Furthermore, when all ncRNA types were integrated, the model's performance was further enhanced, achieving the highest AUC, AUPR, and the lowest Rank Index. These results strongly demonstrate PanGIA's exceptional generalization ability and predictive accuracy in multi-type ncRNA-disease association prediction.

Multi-task synchronous prediction can provide key information

To validate the advantages of our proposed framework in leveraging the full-spectrum heterogeneous association network and the neural network architecture design, we not only examined the performance after ablating critical network modules but also progressively reduced the scale of full-spectrum data to evaluate the unique contribution of pan-ncRNAdisease association information.

Stepwise Reduction of Heterogeneity in the Network

To systematically evaluate the impact of reduced training data on model performance, we designed two downsampling strategies to progressively decrease the amount of information available to the PanGIA model: (1) a random uniform subsampling strategy, and (2) an RNA-type-based node selection strategy.

In the random uniform subsampling strategy, we adopted a straightforward uniform sampling method. Specifically, a certain proportion of ncRNA and disease nodes were randomly and uniformly removed from the heterogeneous graph, along with their associated edges. This ensured that both types of nodes (ncRNAs and diseases) were reduced at the same rate, preserving the relative balance between modalities in the network while decreasing the overall graph size. By gradually scaling down the input network, we simulated scenarios with limited data availability to examine how PanGIA performs under constrained information settings.

Based on this strategy, we conducted a systematic performance evaluation of the PanGIA model using 100%, 80%, 67%, and 50% of the original dataset for training. As shown in Figure 2, the corresponding evaluation metricsAUC, AUPR, and Rank Indexconsistently declined with reduced data scale. These results indicate that PanGIA is sensitive to the quantity of training data and that its predictive capability is notably affected under data-sparse conditions.

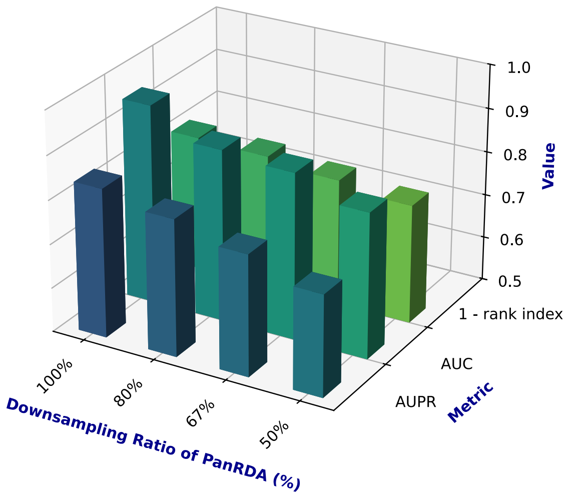

Fig. 2. Performance comparison of PanGIA under different subsampling ratios.

In summary, this experiment underscores the critical importance of data completeness in achieving optimal predictive performance with PanGIA, highlighting the key role

of full-spectrum biological data in robust association prediction tasks.

To further evaluate the overall contribution of different ncRNA types to the predictive performance of the model, we conducted a stepwise ablation study by progressively removing specific categories of ncRNAs from the full pan-ncRNA set. As shown in Figure 3, we assessed model performance under various ncRNA combinations using AUC, AUPR, and Rank Index as evaluation metrics. The results demonstrate that the inclusion of all four ncRNA types (miRNA, lncRNA, circRNA, and piRNA) yields the best overall performance. In contrast, removing any single or multiple ncRNA types leads to a noticeable decline in one or more metrics, highlighting the complementary contributions of each ncRNA class to the overall prediction capability of the PanGIA framework.

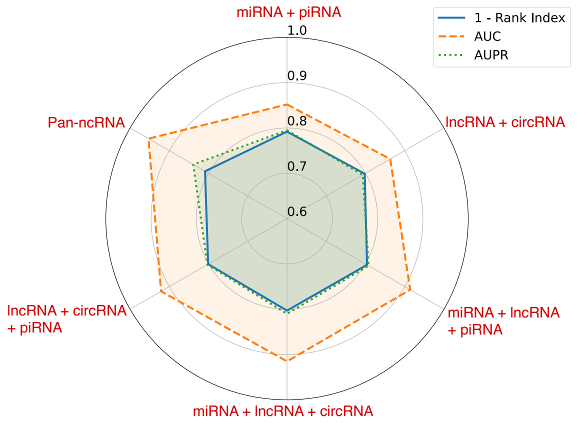

Fig. 3. Performance of PanGIA with different ncRNA combinations.

Robust of PanGIA

To validate the effectiveness of key components in the PanGIA framework, we performed a series of ablation experiments by systematically removing core modules, including the heterogeneous graph attention network (HAN), the Mixture-of-Experts (MoE), and the cross-task attention mechanism. Additionally, a single-task learning variant was tested to contrast against the full multi-task framework. As illustrated in Figure 4, Figure 5 and Figure 6, performance metrics including AUC, AUPR, and Rank Index were measured for each ablation variant.

The results demonstrate that removing any of the core components leads to a noticeable performance decline across all metrics. Specifically, the removal of HAN or MoE resulted in significant drops in both AUC and AUPR, indicating the importance of structural and expert-based representation learning. Furthermore, disabling the cross-task attention mechanism impaired the models ability to integrate information across tasks, reducing prediction accuracy. The single-task baseline also underperformed compared to the full model, highlighting the advantage of PanGIA's multi-task learning design. Overall, these findings confirm that each module contributes uniquely and substantially to the overall predictive power of PanGIA.

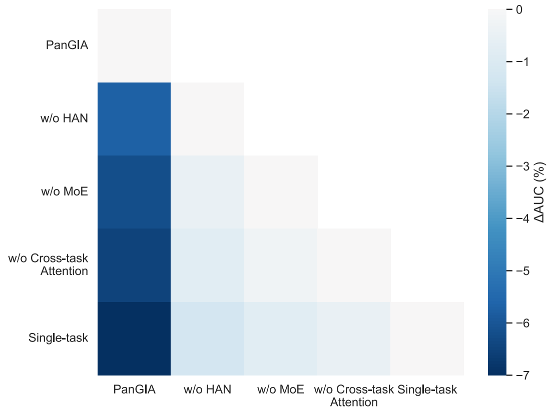

Fig. 4. Performance comparison of PanGIA ablation variants on AUC.

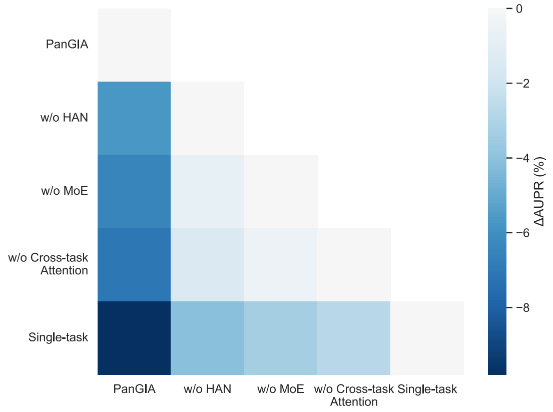

Fig. 5. Performance comparison of PanGIA ablation variants on AUPR.

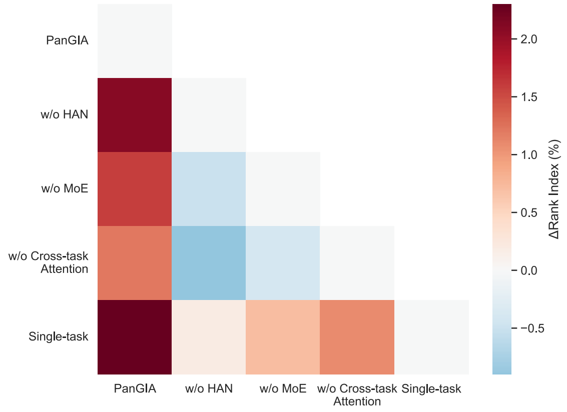

Fig. 6. Performance comparison of PanGIA ablation variants on Rank Index.

PanGIA reveals novel ncRNA-disease associations

In the case study, we selected high-confidence associations between various types of ncRNAs (miRNA, circRNA, lncRNA, and piRNA) and representative diseases as predicted by the PanGIA model. These predicted associations were organized and presented in Table 2, respectively. Through literature

Table 2. Experimentally validated ncRNAdisease associations used in the case study

RNA Symbol	RNA Category	Disease	PMID
miR-944	miRNA	Glioblastoma	34233294
miR-936	miRNA	Glioblastoma	29218238
miR-448	miRNA	Osteoarthritis	29483929
mir-495	miRNA	Idiopathic Pulmonary Fibrosis	32237054
CSPP1	circRNA	Glioblastoma	32495924
SCN3B	circRNA	Glioblastoma	39289188
WNK1	circRNA	Glioblastoma	24555568
YTHDF3	circRNA	Liver Cirrhosis	35779442
LINC00662	lncRNA	Gallbladder carcinoma	38928444
LINC00691	lncRNA	Stomach carcinoma	32330554
RHPN1-AS1	lncRNA	Stomach carcinoma	31982726
KCNQ1OT1	lncRNA	Glioblastoma	28381990
DQ570326	piRNA	Parkinson's disease	29986767
DQ592957	piRNA	Parkinson's disease	29986767
DQ596377	piRNA	Alzheimer's disease	28127595
DQ597397	piRNA	Renal cell carcinoma	25998508

Source: All associations were experimentally validated and supported by the referenced PubMed IDs (PMIDs).

review, we confirmed that all the associations listed in the tables have been experimentally validated, with supporting evidence provided by the corresponding references (PMIDs).

Among the miRNAdisease associations, miR-944 does not act as an oncogenic factor. Instead, it is derived from glioblastoma stem cell-derived exosomes and exerts anti-tumor effects by downregulating VEGFC expression, thereby inhibiting the AKT/ERK signaling pathway. This suppression results in impaired glioblastoma cell proliferation, migration, and angiogenesis [45]. miR-936 is downregulated in glioblastoma tissues. Its overexpression targets CKS1 and inhibits the Akt-ERK1/2 pathway, inducing G1 phase cell cycle arrest and significantly suppressing tumor cell proliferation and growth [46]. In contrast, miR-448 is upregulated in osteoarthritic cartilage and IL-1β-induced chondrocytes, where it promotes extracellular matrix degradation by downregulating matrilin-3; its inhibition is considered beneficial for cartilage matrix preservation [47].

Regarding circRNAs and lncRNAs, CSPP1, SCN3B, and WNK1 have been shown to act as sponges for specific miRNAs, thereby relieving miRNA-mediated suppression of oncogenes and activating downstream signaling pathways that promote glioblastoma progression [48, 49, 50]. YTHDF3 is upregulated in liver cirrhosis tissues and is presumed to participate in the fibrotic process by modulating the function of RNA methylation reader proteins [51]. Among lncRNAs, LINC00662 is overexpressed in gallbladder carcinoma and promotes tumor cell proliferation and migration through pathway activation [52]. LINC00691 and RHPN1-AS1 facilitate tumorigenesis in stomach carcinoma by regulating cell cycle- and apoptosis-related genes [53, 54]. KCNQ1OT1 functions as a sponge for miR-370-3p in glioblastoma, thereby lifting suppression on the STAT3 signaling pathway and enhancing cell proliferation and chemoresistance [55].

PiRNAs, which are widely present in somatic cells, are increasingly recognized for their importance in tumors and neurodegenerative diseases. DQ570326 and DQ592957 show potential associations with Parkinsons disease, suggesting their involvement in neurodegenerative processes [56]. Additionally, the expression changes of DQ596377 in Alzheimers disease and DQ597397 in renal cell carcinoma, with their significant links to tumor pathways, indicate that piRNAs, as novel biomarkers, could provide new directions for disease diagnosis and treatment [57, 58].

This result indicates that PanGIA performs excellently in the aforementioned case studies, demonstrating its capability to identify high-confidence associations between miRNAs, circRNAs, lncRNAs, and piRNAs with diseases. The unconfirmed associations predicted by PanGIA may serve as candidate targets for subsequent biological experiments and lay a solid foundation for the potential application of related ncRNAs in disease diagnosis and therapy.

Conclusion

In this study, we proposed **PanGIA**, a novel model for ncRNAdisease association prediction that integrates a Hierarchical Attention Network (HAN) with a Mixture-of-Experts architecture. Comprehensive experiments demonstrate that PanGIA achieves consistently superior performance across various RNA types, including miRNA, lncRNA, circRNA, and piRNA, validating the effectiveness of our multi-source feature extraction and multi-task modeling strategy.

PanGIA not only outperforms state-of-the-art methods on multiple evaluation metrics but also maintains robust and stable performance across different ncRNA categories. This indicates strong generalization and robustness of the model in handling heterogeneous RNA data and structures.

Through further case study analyses, we validated that several high-confidence predictions have been experimentally confirmed in the literature, highlighting the significant advantages of this method in terms of biological interpretability and result reliability. In particular, the ncRNAdisease associations predicted by PanGIA show substantial research and application value in fields such as neurological disorders, metabolic diseases, and cancer.

Overall, PanGIA demonstrates strong potential as a unified framework for pan-ncRNAdisease association prediction.

It excels in both macro-level performance benchmarks and micro-level case reliability, suggesting excellent cross-task generalizability and interpretability. In future work, incorporating additional omics data and optimizing the network architecture may further enhance its predictive power, contributing to ncRNA functional studies, disease mechanism exploration, and the advancement of precision medicine and biomarker discovery.

Competing interests

No competing interest is declared.

Funding

Heilongjiang Province Basic Research Support Program for Outstanding Young Teachers(YQJH2023195)

Code and data availability

All code and datasets supporting this study are openly accessible on GitHub (github.com/qiankunzizairen/PanGIA) to promote transparency and reproducibility.

References

1. Manel Esteller. Non-coding RNAs in human disease. *Nature Reviews Genetics*, 12(12):861–874, December 2011.
2. Tamizhini Loganathan and George Priya Doss C. Non-coding RNAs in human health and disease: potential function as biomarkers and therapeutic targets. *Functional & Integrative Genomics*, 23(1):33, March 2023.
3. LornaW. Harries. Long non-coding RNAs and human disease. *Biochemical Society Transactions*, 40(4):902–906, August 2012.
4. Chen Li, Yu-Qing Ni, Hui Xu, Qun-Yan Xiang, Yan Zhao, Jun-Kun Zhan, Jie-Yu He, Shuang Li, and You-Shuo Liu. Roles and mechanisms of exosomal non-coding RNAs in human health and diseases. *Signal Transduction and Targeted Therapy*, 6(1):383, November 2021.
5. Hongbo Shi, Juan Xu, Guangde Zhang, Liangde Xu, Chunquan Li, Li Wang, Zheng Zhao, Wei Jiang, Zheng Guo, and Xia Li. Walking the interactome to identify human miRNA-disease associations through the functional link between miRNA targets and disease genes. *BMC Systems Biology*, 7(1):101, December 2013.
6. Ming Lu, Qipeng Zhang, Min Deng, Jing Miao, Yanhong Guo, Wei Gao, and Qinghua Cui. An Analysis of Human MicroRNA and Disease Associations. *PLoS ONE*, 3(10):e3420, October 2008.
7. Jiankun Zang, Dan Lu, and Anding Xu. The interaction of circRNAs and RNA binding proteins: An important part of circRNA maintenance and function. *Journal of Neuroscience Research*, 98(1):87–97, January 2020.
8. Trine Line Hauge Okholm, Shashank Sathe, Samuel S. Park, Andreas Bjerregaard Kamstrup, Asta Mannstaedt Rasmussen, Archana Shankar, Zong Ming Chua, Niels Fristrup, Morten Muhlig Nielsen, Sren Vang, Lars Dyrskjt, Stefan Aigner, Christian Kroun Damgaard, Gene W. Yeo, and Jakob Skou Pedersen. Transcriptome-wide profiles of circular RNA and RNA-binding protein interactions reveal effects on circular RNA biogenesis and cancer pathway expression. *Genome Medicine*, 12(1):112, December 2020.
9. Jing Yan, Ruobing Wang, and Jianjun Tan. Recent advances in predicting lncRNAdisease associations based on computational methods. *Drug Discovery Today*, 28(2):103432, February 2023.
10. Xiaofei Yang, Lin Gao, Xingli Guo, Xinghua Shi, Hao Wu, Fei Song, and Bingbo Wang. A Network Based Method for Analysis of lncRNA-Disease Associations and Prediction of lncRNAs Implicated in Diseases. *PLOS ONE*, 9(1):e87797, January 2014.
11. Syed Danish Ali, Hilal Tayara, and Kil To Chong. Identification of piRNA disease associations using deep learning. *Computational and Structural Biotechnology Journal*, 20:1208–1217, January 2022.
12. Kayla J. Rayford, Ayorinde Cooley, Jelonia T. Rumph, Ashutosh Arun, Girish Rachakonda, Fernando Villalta, Maria F. Lima, Siddharth Pratap, Smita Misra, and Pius N. Nde. piRNAs as Modulators of Disease Pathogenesis. *International Journal of Molecular Sciences*, 22(5):2373, February 2021.
13. Zejun Li, Yuxiang Zhang, Yuting Bai, Xiaohui Xie, Lijun Zeng, Zejun Li, Yuxiang Zhang, Yuting Bai, Xiaohui Xie, and Lijun Zeng. IMC-MDA: Prediction of miRNA-disease association based on induction matrix completion. *Mathematical Biosciences and Engineering*, 20(6):10659–10674, 2023.
14. Yongtian Wang, Liran Juan, Jiajie Peng, Tianyi Zang, and Yadong Wang. LncDisAP: A computation model for LncRNA-disease association prediction based on multiple biological datasets. *BMC Bioinformatics*, 20:582, December 2019.
15. Yurong Qian, Qihua He, and Lei Deng. iPiDA-GBNN:Identification of Piwi-interacting RNA-disease associations based on gradient boosting neural network. In *2021 IEEE International Conference on Bioinformatics and Biomedicine (BIBM)*, pages 1045–1050, December 2021.
16. Lei Wang, Zhu-Hong You, Yang-Ming Li, Kai Zheng, and Yu-An Huang. GCNCDA: A new method for predicting circRNA-disease associations based on Graph Convolutional Network Algorithm. *PLoS computational biology*, 16(5):e1007568, May 2020.
17. Zhenyu Bao, Zhen Yang, Zhou Huang, Yiran Zhou, Qinghua Cui, and Dong Dong. LncRNADisease 2.0: an updated database of long non-coding RNA-associated diseases. *Nucleic Acids Research*, 47(D1):D1034–D1037, January 2019.
18. Chunyan Fan, Xiujuan Lei, Jiaojiao Tie, Yuchen Zhang, Fang-Xiang Wu, and Yi Pan. CircR2Disease v2.0: An Updated Web Server for Experimentally Validated circRNAdisease Associations and Its Application. *Genomics, Proteomics & Bioinformatics*, 20(3):435–445, June 2022.
19. Azhar Muhammad, Ramay Waheed, Nauman Ali Khan, Hong Jiang, and Xiaoyuan Song. piRDisease v1.0: a manually curated database for piRNA associated diseases. *Database: The Journal of Biological Databases and Curation*, 2019:baz052, January 2019.
20. Qinghua Jiang, Yadong Wang, Yangyang Hao, Liran Juan, Mingxiang Teng, Xinjun Zhang, Meimei Li, Guohua Wang, and Yunlong Liu. miR2Disease: a manually curated database for microRNA deregulation in human disease. *Nucleic Acids Research*, 37(Database issue):D98–104, January 2009.

21. Sonja Hombach and Markus Kretz. Non-coding RNAs: Classification, Biology and Functioning. *Advances in Experimental Medicine and Biology*, 937:3–17, 2016.
22. Chunmei Cui, Bitao Zhong, Rui Fan, and Qinghua Cui. HMDD v4.0: a database for experimentally supported human microRNA-disease associations. *Nucleic Acids Research*, 52(D1):D1327–D1332, January 2024.
23. Ana Kozomara, Maria Birgaoanu, and Sam Griffiths-Jones. miRBase: from microRNA sequences to function. *Nucleic Acids Research*, 47(D1):D155–D162, January 2019.
24. Xiao Lin, Yingyu Lu, Chenhao Zhang, Qinghua Cui, Yi-Da Tang, Xiangwen Ji, and Chunmei Cui. LncRNADisease v3.0: an updated database of long non-coding RNA-associated diseases. *Nucleic Acids Research*, 52(D1):D1365–D1369, January 2024.
25. Petar Glaar, Panagiotis Papavasileiou, and Nikolaus Rajewsky. circBase: a database for circular RNAs. *RNA (New York, N.Y.)*, 20(11):1666–1670, November 2014.
26. Adam Frankish, Slvia Carbonell-Sala, Mark Diekhans, Irwin Jungreis, Jane E. Loveland, Jonathan M. Mudge, Cristina Sisu, James C. Wright, Carme Arnan, If Barnes, Abhimanyu Banerjee, Ruth Bennett, Andrew Berry, Alexandra Bignell, Carles Boix, Ferriol Calvet, Daniel Cerdn-Vlez, Fiona Cunningham, Claire Davidson, Sarah Donaldson, Cagatay Dursun, Reham Fatima, Stefano Giorgetti, Carlos Garca Giron, Jose Manuel Gonzalez, Matthew Hardy, Peter W. Harrison, Thibaut Hourlier, Zoe Hollis, Toby Hunt, Benjamin James, Yunzhe Jiang, Rory Johnson, Mike Kay, Julien Lagarde, Fergal J. Martin, Laura Martnez Gmez, Surag Nair, Pengyu Ni, Fernando Pozo, Vivek Ramalingam, Magali Ruffier, Bianca M. Schmitt, Jacob M. Schreiber, Emily Steed, Marie-Marthe Suner, Dulika Sumathipala, Irina Sycheva, Barbara Uszczynska-Ratajczak, Elizabeth Wass, Yucheng T. Yang, Andrew Yates, Zahoor Zafrulla, Jyoti S. Choudhary, Mark Gerstein, Roderic Guigo, Tim J. P. Hubbard, Manolis Kellis, Anshul Kundaje, Benedict Paten, Michael L. Tress, and Paul Flicek. GENCODE: reference annotation for the human and mouse genomes in 2023. *Nucleic Acids Research*, 51(D1):D942–D949, January 2023.
27. Yi Zhao, Hui Li, Shuangsang Fang, Yue Kang, Wei Wu, Yajing Hao, Ziyang Li, Dechao Bu, Ninghui Sun, Michael Q. Zhang, and Runsheng Chen. NONCODE 2016: an informative and valuable data source of long non-coding RNAs. *Nucleic Acids Research*, 44(D1):D203–208, January 2016.
28. Jiajia Wang, Peng Zhang, Yiping Lu, Yanyan Li, Yu Zheng, Yunchao Kan, Runsheng Chen, and Shunmin He. piRBase: a comprehensive database of piRNA sequences. *Nucleic Acids Research*, 47(D1):D175–D180, January 2019.
29. Ricardo Piuco and Pedro A. F. Galante. piRNAdb: A piwi-interacting RNA database, September 2021.
30. Lynn M. Schriml, James B. Munro, Mike Schor, Dustin Olley, Carrie McCracken, Victor Felix, J. Allen Baron, Rebecca Jackson, Susan M. Bello, Cynthia Bearer, Richard Lichenstein, Katharine Bisordi, Nicole Campion Dialo, Michelle Giglio, and Carol Greene. The Human Disease Ontology 2022 update. *Nucleic Acids Research*, 50(D1):D1255–D1261, January 2022.
31. Yi-Chen Chen, Sung-Feng Huang, Hung-yi Lee, Yu-Hsuan Wang, and Chia-Hao Shen. Audio Word2vec: Sequence-to-Sequence Autoencoding for Unsupervised Learning of Audio Segmentation and Representation. *IEEE/ACM Transactions on Audio, Speech, and Language Processing*, 27(9):1481–1493, September 2019.
32. Yu-An Chung, Chao-Chung Wu, Chia-Hao Shen, Hung-Yi Lee, and Lin-Shan Lee. Audio Word2Vec: Unsupervised Learning of Audio Segment Representations using Sequence-to-sequence Autoencoder, June 2016. arXiv:1603.00982.
33. Julien Lagarde, Barbara Uszczynska-Ratajczak, Silvia Carbonell, Slvia Prez-Lluch, Amaya Abad, Carrie Davis, Thomas R. Gingeras, Adam Frankish, Jennifer Harrow, Roderic Guigo, and Rory Johnson. High-throughput annotation of full-length long noncoding RNAs with capture long-read sequencing. *Nature Genetics*, 49(12):1731–1740, December 2017.
34. Twan Van Laarhoven, Sander B. Nabuurs, and Elena Marchiori. Gaussian interaction profile kernels for predicting drugtarget interaction. *Bioinformatics*, 27(21):3036–3043, November 2011.
35. Sebastian Khler. Improved ontology-based similarity calculations using a study-wise annotation model. *Database*, 2018, January 2018.
36. Sachin Mathur and Deendayal Dinakarpandian. Finding disease similarity based on implicit semantic similarity. *Journal of Biomedical Informatics*, 45(2):363–371, April 2012.
37. Chen Jin, Zhuangwei Shi, Ken Lin, and Han Zhang. Predicting miRNA-Disease Association Based on Neural Inductive Matrix Completion with Graph Autoencoders and Self-Attention Mechanism. *Biomolecules*, 12(1):64, January 2022.
38. Zhengzheng Lou, Zhaoxu Cheng, Hui Li, Zhixia Teng, Yang Liu, and Zhen Tian. Predicting miRNAdisease associations via learning multimodal networks and fusing mixed neighborhood information. *Briefings in Bioinformatics*, 23(5):bbac159, September 2022.
39. Li Wang and Cheng Zhong. gGATLDA: lncRNA-disease association prediction based on graph-level graph attention network. *BMC Bioinformatics*, 23(1):11, January 2022.
40. Xiaowei Zhao, Yiqin Yang, and Minghao Yin. MHRWR: Prediction of lncRNA-Disease Associations Based on Multiple Heterogeneous Networks. *IEEE/ACM Transactions on Computational Biology and Bioinformatics*, 18(6):2577–2585, November 2021.
41. Hang Wei, Yong Xu, and Bin Liu. iPiDi-PUL: identifying Piwi-interacting RNA-disease associations based on positive unlabeled learning. *Briefings in Bioinformatics*, 22(3):bbaa058, May 2021.
42. Qiuhao Chen, Liyuan Zhang, Yaojia Liu, Zhonghao Qin, and Tianyi Zhao. PUTransGCN: identification of piRNAdisease associations based on attention encoding graph convolutional network and positive unlabelled learning. *Briefings in Bioinformatics*, 25(3):bbae144, March 2024.
43. Wei Lan, Yi Dong, Qingfeng Chen, Jin Liu, Jianxin Wang, Yi-Ping Phoebe Chen, and Shirui Pan. IGNSCDA: Predicting CircRNA-Disease Associations Based on Improved Graph Convolutional Network and Negative Sampling. *IEEE/ACM Transactions on Computational Biology and Bioinformatics*, 19(6):3530–3538, November 2022.
44. Li Peng, Cheng Yang, Yifan Chen, and Wei Liu. Predicting CircRNA-Disease Associations via Feature Convolution Learning With Heterogeneous Graph Attention Network. *IEEE Journal of Biomedical and Health Informatics*, 27(6):3072–3082, June 2023.

45. Jianxin Jiang, Jun Lu, Xiaolin Wang, Bing Sun, Xiaoxing Liu, Yasuo Ding, and Guangzhong Gao. Glioma stem cell-derived exosomal miR-944 reduces glioma growth and angiogenesis by inhibiting AKT/ERK signaling. *Aging*, 13(15):19243–19259, July 2021.
46. Dong Wang, Tongle Zhi, Xiupeng Xu, Zhongyuan Bao, Liang Fan, Zheng Li, Jing Ji, and Ning Liu. MicroRNA-936 induces cell cycle arrest and inhibits glioma cell proliferation by targeting CKS1. *American Journal of Cancer Research*, 7(11):2131–2143, November 2017.
47. Hao Yang, Di Wu, Hua Li, Nan Chen, and Yongjun Shang. Downregulation of microRNA-448 inhibits IL-1-induced cartilage degradation in human chondrocytes via upregulation of matrilin-3. *Cellular & Molecular Biology Letters*, 23:7, 2018.
48. Y.-F. Xue, M. Li, W. Li, Q. Lin, B.-X. Yu, Q.-B. Zhu, and H.-J. Chen. Roles of circ-CSPP1 on the proliferation and metastasis of glioma cancer. *European Review for Medical and Pharmacological Sciences*, 24(10):5519–5525, May 2020.
49. Hengrui Liu, Jieling Weng, Christopher L.-H. Huang, and Antony P. Jackson. Is the voltage-gated sodium channel 3 subunit (SCN3B) a biomarker for glioma? *Functional & Integrative Genomics*, 24(5):162, September 2024.
50. Wen Zhu, Gulnaz Begum, Kelli Pointer, Paul A. Clark, Sung-Sen Yang, Shih-Hua Lin, Kristopher T. Kahle, John S. Kuo, and Dandan Sun. WNK1-OSR1 kinase-mediated phospho-activation of Na+-K+-2Cl-cotransporter facilitates glioma migration. *Molecular Cancer*, 13:31, February 2014.
51. Ruimin Sun, Xinyao Tian, Yang Li, Yan Zhao, Zhecheng Wang, Yan Hu, Lijun Zhang, Yue Wang, Dongyan Gao, Shusen Zheng, and Jihong Yao. The m6A reader YTHDF3-mediated PRDX3 translation alleviates liver fibrosis. *Redox Biology*, 54:102378, August 2022.
52. Pablo Prez-Moreno, Ismael Riquelme, Carolina Bizama, Luis Vergara-Gmez, Julio C. Tapia, Priscilla Brebi, Patricia Garca, and Juan Carlos Roa. LINC00662 Promotes Aggressive Traits by Modulating OCT4 Expression through miR-335-5p in Gallbladder Cancer Cells. *International Journal of Molecular Sciences*, 25(12):6740, June 2024.
53. Wei Liang, Bin Xia, Chao He, Guanghua Zhai, Meifen Li, and Jundong Zhou. Overexpression of LINC00691 promotes the proliferation and invasion of gastric cancer cells via the Janus kinase/signal transducer and activator of transcription signalling pathway. *The International Journal of Biochemistry & Cell Biology*, 123:105751, June 2020.
54. Lei Ding, Ling Wang, Zhiqi Li, Xuefeng Jiang, Yangchun Xu, and Ning Han. The positive feedback loop of RHPN1-AS1/miR-1299/ETS1 accelerates the deterioration of gastric cancer. *Biomedicine & Pharmacotherapy = Biomedecine & Pharmacotherapie*, 124:109848, April 2020.
55. Wei Gong, Jian Zheng, Xiaobai Liu, Yunhui Liu, Junqing Guo, Yana Gao, Wei Tao, Jiajia Chen, Zhiqing Li, Jun Ma, and Yixue Xue. Knockdown of Long Non-Coding RNA KCNQ1OT1 Restrained Glioma Cells' Malignancy by Activating miR-370/CCNE2 Axis. *Frontiers in Cellular Neuroscience*, 11:84, 2017.
56. Markus Schulze, Annika Sommer, Sonja Pltz, Michaela Farrell, Beate Winner, Janina Grosch, Jrgen Winkler, and Markus J. Riemenschneider. Sporadic Parkinson's disease derived neuronal cells show disease-specific mRNA and small RNA signatures with abundant deregulation of piRNAs. *Acta Neuropathologica Communications*, 6(1):58, July 2018.
57. Jyoti Roy, Arijita Sarkar, Sibun Parida, Zhumur Ghosh, and Bibekanand Mallick. Small RNA sequencing revealed dysregulated piRNAs in Alzheimer's disease and their probable role in pathogenesis. *Molecular BioSystems*, 13(3):565–576, 2017.
58. Yuping Li, Xiwei Wu, Hanlin Gao, Jennifer M. Jin, Arthur X. Li, Young S. Kim, Sumanta K. Pal, Rebecca A. Nelson, Clayton M. Lau, Chao Guo, Bing Mu, Jinhui Wang, Frances Wang, Jessica Wang, Yuanyin Zhao, Wengang Chen, John J. Rossi, Lawrence M. Weiss, and Huiqing Wu. Piwi-Interacting RNAs (piRNAs) Are Dysregulated in Renal Cell Carcinoma and Associated with Tumor Metastasis and Cancer-Specific Survival. *Molecular Medicine*, 21(1):381–388, January 2015.
