# Supplementary material for: PanGIA: A universal framework for identifying association between ncRNAs and diseases
Source: Gigascience. 2025 Oct 17;14:giaf123. doi: 10.1093/gigascience/giaf123 (PMC12532321; doi:10.1093/gigascience/giaf123)
Supplement: giaf123_GIGA-D-25-00208_Revision_1 [file giaf123_giga-d-25-00208_revision_1.pdf]

GigaScience

PanGIA: A universal framework for identifying association between ncRNAs and diseases

--Manuscript Draft--

Manuscript Number:	GIGA-D-25-00208R1
Full Title:	PanGIA: A universal framework for identifying association between ncRNAs and diseases
Article Type:	Research
Funding Information:	Heilongjiang Province Basic Research Support Program (YQJH2023195) — Mr. Xiaoyuan Liu
Abstract:	With the increasing recognition of the crucial roles that non-coding RNAs (ncRNAs) play in various biological processes—particularly their potential involvement in numerous human diseases—the effective prediction of ncRNA–disease associations has become a critical issue in biomedical research. Although numerous computational methods have been proposed to predict associations between non-coding RNAs (ncRNAs) and diseases, most of these approaches focus exclusively on a single class of ncRNAs. However, competitive and cooperative interactions among different types of ncRNAs are closely related to their functional roles in disease associations. To address this limitation, we propose a novel computational framework, PanGIA (Pan-ncRNA Graph-Interaction Attention network), which is designed to simultaneously predict potential associations between multiple types of non-coding RNAs—including miRNA, lncRNA, circRNA, and piRNA—and diseases. This method innovatively integrates the Heterogeneous Graph Attention Network (HAN) with the Mixture of Experts (MoE) framework. By constructing a unified cross-modal heterogeneous graph, it effectively incorporates diverse data sources, including ncRNA sequence features, functional annotations, and interaction networks between ncRNAs and diseases. Experiments show that PanGIA achieves higher accuracy than typespecific models in both individual and comprehensive predictions. It remains robust under node or ncRNA-type removal, and ablation studies confirm the benefit of cross-type information. PanGIA improves AUC and AUPR by up to 7.1% and 6.5%, respectively, over single-type sota methods. Case studies on top-ranked predictions further validate the model's biological relevance with prior experimental evidence. In particular, it shows significant advantages in predicting disease associations for different types of ncRNA, including miRNA, lncRNA, circRNA, and piRNA. The case studies further validated the accuracy of the model's predictions, as all high-confidence associations were supported by literature evidence. This demonstrates the model's strong biological interpretability and promising potential for practical applications. The successful application of PanGIA offers a new paradigm for exploring disease-associated ncRNAs, highlighting their great potential in the field of biomedical research.
Corresponding Author:	Tianyi Zhao<br>Harbin Institute of Technology<br>harbin, CHINA
Corresponding Author Secondary Information:	
Corresponding Author's Institution:	Harbin Institute of Technology
Corresponding Author's Secondary Institution:	
First Author:	Xiaoyuan Liu
First Author Secondary Information:	
Order of Authors:	Xiaoyuan Liu
	Xiye Lü
	Qiuhao Chen

Powered by Editorial Manager® and ProduXion Manager® from Aries Systems Corporation

	Jiqiu Sun
	Tianyi Zhao
	Yan Zhu
**Order of Authors Secondary Information:**	
**Response to Reviewers:**	We provide a full point-by-point response in the attached file “Review_Response_Letter.pdf”.
**Additional Information:**	
**Question**	**Response**
Are you submitting this manuscript to a special series or article collection?	No
**Experimental design and statistics**<br><br>Full details of the experimental design and statistical methods used should be given in the Methods section, as detailed in our Minimum Standards Reporting Checklist. Information essential to interpreting the data presented should be made available in the figure legends.<br><br>Have you included all the information requested in your manuscript?	Yes
**Resources**<br><br>A description of all resources used, including antibodies, cell lines, animals and software tools, with enough information to allow them to be uniquely identified, should be included in the Methods section. Authors are strongly encouraged to cite Research Resource Identifiers (RRIDs) for antibodies, model organisms and tools, where possible.<br><br>Have you included the information requested as detailed in our Minimum Standards Reporting Checklist?	Yes
**Availability of data and materials**<br><br>All datasets and code on which the conclusions of the paper rely must be either included in your submission or	Yes

deposited in publicly available repositories (where available and ethically appropriate), referencing such data using a unique identifier in the references and in the "Availability of Data and Materials" section of your manuscript.<br><br>Have you have met the above requirement as detailed in our Minimum Standards Reporting Checklist?	
GigaScience has policies and guidelines in place for the use of generative AI-writing tools such as ChatGPT. If you have used such writing tools to assist with writing the manuscript this must be declared and cited in the text. Authors should not list AI-writing tools and other AI-assisted technologies as an author or co-author and should acknowledge that they are fully responsible for text generated or refined by AI-writing tools.<p><br><br>A summary of use (particularly in the introduction or among methods) needs to be included at the end of the paper, and the outputs should also be included as a supplementary file hosted in GigaDB or other open repositories. Please read our guidelines for more information. <p><br><br>By submitting to GigaScience, you are aware of the journal's AI-writing tools policy, and if you have declared use of such tools below, you have acknowledged this where appropriate in your manuscript and have made a summary of use and outputs available. <p><br><b>AI-assisted writing tools have been used in the preparation of this manuscript?	Yes

Powered by Editorial Manager® and ProduXion Manager® from Aries Systems Corporation

LaTeX - Main Document (TeX file) Click here to access/download;LaTeX - Main Document (TeX file);main.tex

```
This is pdfTeX, Version 3.141592653-2.6-1.40.26 (TeX Live 2024)
(preloaded format=pdflatex 2024.8.2)  28 SEP 2025 23:26
entering extended mode
 restricted \write18 enabled.
 %&-line parsing enabled.
**main.tex
(./main.tex
LaTeX2e <2024-06-01> patch level 2
L3 programming layer <2024-05-27>
(./oup-contemporary.cls
Document Class: oup-contemporary 2023/06/12, v1.2
(c:/texlive/2024/texmf-dist/tex/latex/base/article.cls
Document Class: article 2024/02/08 v1.4n Standard LaTeX document class
(c:/texlive/2024/texmf-dist/tex/latex/base/size10.clo
File: size10.clo 2024/02/08 v1.4n Standard LaTeX file (size option)
)
\c@part=\count194
\c@section=\count195
\c@subsection=\count196
\c@subsubsection=\count197
\c@paragraph=\count198
\c@subparagraph=\count199
\c@figure=\count266
\c@table=\count267
\abovecaptionskip=\skip49
\belowcaptionskip=\skip50
\bibindent=\dimen141
) (c:/texlive/2024/texmf-dist/tex/latex/base/inputenc.sty
Package: inputenc 2024/02/08 v1.3d Input encoding file
\inpenc@prehook=\toks17
\inpenc@posthook=\toks18
) (c:/texlive/2024/texmf-dist/tex/latex/base/fontenc.sty
Package: fontenc 2021/04/29 v2.0v Standard LaTeX package
) (c:/texlive/2024/texmf-dist/tex/generic/iftex/ifpdf.sty
Package: ifpdf 2019/10/25 v3.4 ifpdf legacy package. Use iftex instead.
(c:/texlive/2024/texmf-dist/tex/generic/iftex/iftex.sty
Package: iftex 2022/02/03 v1.0f TeX engine tests
)) (c:/texlive/2024/texmf-dist/tex/latex/microtype/microtype.sty
Package: microtype 2024/03/29 v3.1b Micro-typographical refinements (RS)
(c:/texlive/2024/texmf-dist/tex/latex/graphics/keyval.sty
Package: keyval 2022/05/29 v1.15 key=value parser (DPC)
\KV@toks@=\toks19
) (c:/texlive/2024/texmf-dist/tex/latex/etoolbox/etoolbox.sty
Package: etoolbox 2020/10/05 v2.5k e-TeX tools for LaTeX (JAW)
\etb@tempcnta=\count268
)
\MT@toks=\toks20
\MT@tempbox=\box52
\MT@count=\count269
LaTeX Info: Redefining \noprotrusionifhmode on input line 1061.
LaTeX Info: Redefining \leftprotrusion on input line 1062.
\MT@prot@toks=\toks21
LaTeX Info: Redefining \rightprotrusion on input line 1081.
LaTeX Info: Redefining \textls on input line 1392.
```

```
\MT@outer@kern=\dimen142
LaTeX Info: Redefining \textmicrotypecontext on input line 2013.
\MT@listname@count=\count270
(c:/texlive/2024/texmf-dist/tex/latex/microtype/microtype-pdftex.def
File: microtype-pdftex.def 2024/03/29 v3.1b Definitions specific to
pdftex (RS)

LaTeX Info: Redefining \lsstyle on input line 902.
LaTeX Info: Redefining \lslig on input line 902.
\MT@outer@space=\skip51
)
Package microtype Info: Loading configuration file microtype.cfg.
(c:/texlive/2024/texmf-dist/tex/latex/microtype/microtype.cfg
File: microtype.cfg 2024/03/29 v3.1b microtype main configuration file
(RS)
)) (c:/texlive/2024/texmf-dist/tex/latex/euler/euler.sty
Package: euler 1995/03/05 v2.5
Package: `euler' v2.5 <1995/03/05> (FJ and FMi)
LaTeX Font Info:    Redeclaring symbol font `letters' on input line 35.
LaTeX Font Info:    Encoding `OML' has changed to `U' for symbol font
(Font)              `letters' in the math version `normal' on input line
35.
LaTeX Font Info:    Overwriting symbol font `letters' in version `normal'
(Font)                  OML/cmm/m/it --> U/eur/m/n on input line 35.
LaTeX Font Info:    Encoding `OML' has changed to `U' for symbol font
(Font)              `letters' in the math version `bold' on input line
35.
LaTeX Font Info:    Overwriting symbol font `letters' in version `bold'
(Font)                  OML/cmm/b/it --> U/eur/m/n on input line 35.
LaTeX Font Info:    Overwriting symbol font `letters' in version `bold'
(Font)                  U/eur/m/n --> U/eur/b/n on input line 36.
LaTeX Font Info:    Redeclaring math symbol \Gamma on input line 47.
LaTeX Font Info:    Redeclaring math symbol \Delta on input line 48.
LaTeX Font Info:    Redeclaring math symbol \Theta on input line 49.
LaTeX Font Info:    Redeclaring math symbol \Lambda on input line 50.
LaTeX Font Info:    Redeclaring math symbol \Xi on input line 51.
LaTeX Font Info:    Redeclaring math symbol \Pi on input line 52.
LaTeX Font Info:    Redeclaring math symbol \Sigma on input line 53.
LaTeX Font Info:    Redeclaring math symbol \Upsilon on input line 54.
LaTeX Font Info:    Redeclaring math symbol \Phi on input line 55.
LaTeX Font Info:    Redeclaring math symbol \Psi on input line 56.
LaTeX Font Info:    Redeclaring math symbol \Omega on input line 57.
\symEulerFraktur=\mathgroup4
LaTeX Font Info:    Overwriting symbol font `EulerFraktur' in version
`bold'
(Font)                  U/euf/m/n --> U/euf/b/n on input line 63.
LaTeX Info: Redefining \oldstylenums on input line 85.
\symEulerScript=\mathgroup5
LaTeX Font Info:    Overwriting symbol font `EulerScript' in version
`bold'
(Font)                  U/eus/m/n --> U/eus/b/n on input line 93.
LaTeX Font Info:    Redeclaring math symbol \aleph on input line 97.
LaTeX Font Info:    Redeclaring math symbol \Re on input line 98.
LaTeX Font Info:    Redeclaring math symbol \Im on input line 99.
```

```
LaTeX Font Info:    Redeclaring math delimiter \vert on input line 101.
LaTeX Font Info:    Redeclaring math delimiter \backslash on input line
103.
LaTeX Font Info:    Redeclaring math symbol \neg on input line 106.
LaTeX Font Info:    Redeclaring math symbol \wedge on input line 108.
LaTeX Font Info:    Redeclaring math symbol \vee on input line 110.
LaTeX Font Info:    Redeclaring math symbol \setminus on input line 112.
LaTeX Font Info:    Redeclaring math symbol \sim on input line 113.
LaTeX Font Info:    Redeclaring math symbol \mid on input line 114.
LaTeX Font Info:    Redeclaring math delimiter \arrowvert on input line
116.
LaTeX Font Info:    Redeclaring math symbol \mathsection on input line
117.
\symEulerExtension=\mathgroup6
LaTeX Font Info:    Redeclaring math symbol \coprod on input line 125.
LaTeX Font Info:    Redeclaring math symbol \prod on input line 125.
LaTeX Font Info:    Redeclaring math symbol \sum on input line 125.
LaTeX Font Info:    Redeclaring math symbol \intop on input line 130.
LaTeX Font Info:    Redeclaring math symbol \ointop on input line 131.
LaTeX Font Info:    Redeclaring math symbol \braceld on input line 132.
LaTeX Font Info:    Redeclaring math symbol \bracerd on input line 133.
LaTeX Font Info:    Redeclaring math symbol \bracelu on input line 134.
LaTeX Font Info:    Redeclaring math symbol \braceru on input line 135.
LaTeX Font Info:    Redeclaring math symbol \infty on input line 136.
LaTeX Font Info:    Redeclaring math symbol \nearrow on input line 153.
LaTeX Font Info:    Redeclaring math symbol \searrow on input line 154.
LaTeX Font Info:    Redeclaring math symbol \nwarrow on input line 155.
LaTeX Font Info:    Redeclaring math symbol \swarrow on input line 156.
LaTeX Font Info:    Redeclaring math symbol \Leftrightarrow on input line
157.
LaTeX Font Info:    Redeclaring math symbol \Leftarrow on input line 158.
LaTeX Font Info:    Redeclaring math symbol \Rightarrow on input line
159.
LaTeX Font Info:    Redeclaring math symbol \leftrightarrow on input line
160.
LaTeX Font Info:    Redeclaring math symbol \leftarrow on input line 161.
LaTeX Font Info:    Redeclaring math symbol \rightarrow on input line
163.
LaTeX Font Info:    Redeclaring math delimiter \uparrow on input line
166.
LaTeX Font Info:    Redeclaring math delimiter \downarrow on input line
168.
LaTeX Font Info:    Redeclaring math delimiter \updownarrow on input line
170.
LaTeX Font Info:    Redeclaring math delimiter \Uparrow on input line
172.
LaTeX Font Info:    Redeclaring math delimiter \Downarrow on input line
174.
LaTeX Font Info:    Redeclaring math delimiter \Updownarrow on input line
176.
LaTeX Font Info:    Redeclaring math symbol \leftharpoonup on input line
177.
LaTeX Font Info:    Redeclaring math symbol \leftharpoondown on input
line 178.
```

```
LaTeX Font Info:    Redeclaring math symbol \rightharpoonup on input line
179.
LaTeX Font Info:    Redeclaring math symbol \rightharpoondown on input
line 180
.
LaTeX Font Info:    Redeclaring math delimiter \lbrace on input line 182.
LaTeX Font Info:    Redeclaring math delimiter \rbrace on input line 184.
\symcmmigroup=\mathgroup7
LaTeX Font Info:    Overwriting symbol font `cmmigroup' in version `bold'
(Font)                  OML/cmm/m/it --> OML/cmm/b/it on input line 200.
LaTeX Font Info:    Redeclaring math accent \vec on input line 201.
LaTeX Font Info:    Redeclaring math symbol \triangleleft on input line
202.
LaTeX Font Info:    Redeclaring math symbol \triangleright on input line
203.
LaTeX Font Info:    Redeclaring math symbol \star on input line 204.
LaTeX Font Info:    Redeclaring math symbol \lhook on input line 205.
LaTeX Font Info:    Redeclaring math symbol \rhook on input line 206.
LaTeX Font Info:    Redeclaring math symbol \flat on input line 207.
LaTeX Font Info:    Redeclaring math symbol \natural on input line 208.
LaTeX Font Info:    Redeclaring math symbol \sharp on input line 209.
LaTeX Font Info:    Redeclaring math symbol \smile on input line 210.
LaTeX Font Info:    Redeclaring math symbol \frown on input line 211.
LaTeX Font Info:    Redeclaring math accent \grave on input line 245.
LaTeX Font Info:    Redeclaring math accent \acute on input line 246.
LaTeX Font Info:    Redeclaring math accent \tilde on input line 247.
LaTeX Font Info:    Redeclaring math accent \ddot on input line 248.
LaTeX Font Info:    Redeclaring math accent \check on input line 249.
LaTeX Font Info:    Redeclaring math accent \breve on input line 250.
LaTeX Font Info:    Redeclaring math accent \bar on input line 251.
LaTeX Font Info:    Redeclaring math accent \dot on input line 252.
LaTeX Font Info:    Redeclaring math accent \hat on input line 254.
) (c:/texlive/2024/texmf-dist/tex/latex/merriweather/merriweather.sty
Package: merriweather 2022/09/20 (Bob Tennent) Supports
Merriweather(Sans) font
s for all LaTeX engines.
(c:/texlive/2024/texmf-dist/tex/generic/iftex/ifxetex.sty
Package: ifxetex 2019/10/25 v0.7 ifxetex legacy package. Use iftex
instead.
) (c:/texlive/2024/texmf-dist/tex/generic/iftex/ifluatex.sty
Package: ifluatex 2019/10/25 v1.5 ifluatex legacy package. Use iftex
instead.
) (c:/texlive/2024/texmf-dist/tex/latex/base/textcomp.sty
Package: textcomp 2024/04/24 v2.1b Standard LaTeX package
) (c:/texlive/2024/texmf-dist/tex/latex/xkeyval/xkeyval.sty
Package: xkeyval 2022/06/16 v2.9 package option processing (HA)
(c:/texlive/2024/texmf-dist/tex/generic/xkeyval/xkeyval.tex
(c:/texlive/2024/te
xmf-dist/tex/generic/xkeyval/xkvutils.tex
\XKV@toks=\toks22
\XKV@tempa@toks=\toks23
)
\XKV@depth=\count271
```

```
File: xkeyval.tex 2014/12/03 v2.7a key=value parser (HA)
)) (c:/texlive/2024/texmf-dist/tex/latex/base/fontenc.sty
Package: fontenc 2021/04/29 v2.0v Standard LaTeX package
) (c:/texlive/2024/texmf-dist/tex/latex/fontaxes/fontaxes.sty
Package: fontaxes 2020/07/21 v1.0e Font selection axes
LaTeX Info: Redefining \upshape on input line 29.
LaTeX Info: Redefining \itshape on input line 31.
LaTeX Info: Redefining \slshape on input line 33.
LaTeX Info: Redefining \swshape on input line 35.
LaTeX Info: Redefining \scshape on input line 37.
LaTeX Info: Redefining \sscshape on input line 39.
LaTeX Info: Redefining \ulcshape on input line 41.
LaTeX Info: Redefining \textsw on input line 47.
LaTeX Info: Redefining \textssc on input line 48.
LaTeX Info: Redefining \textulc on input line 49.
)) (c:/texlive/2024/texmf-dist/tex/latex/mathastext/mathastext.sty
Package: mathastext 2024/07/27 v1.4b Use the text font in math mode (JFB)

Package mathastext Info: Starting the math mode configuration.
\mst@exists@muskip=\muskip17
\mst@forall@muskip=\muskip18
\mst@prime@muskip=\muskip19
\mst@do@nonletters=\toks24
\mst@undo@nonletters=\toks25
\mst@do@easynonletters=\toks26
\mst@undo@easynonletters=\toks27
\symmtoperatorfont=\mathgroup8
\symmtletterfont=\mathgroup9
(   mathastext:   ) ! and ?
(   mathastext:   ) punctuation: , . : ; and \colon
LaTeX Info: Redefining \relbar on input line 1201.
LaTeX Info: Redefining \rightarrowfill on input line 1202.
LaTeX Info: Redefining \leftarrowfill on input line 1205.
(   mathastext:   ) + and =
LaTeX Info: Redefining \Relbar on input line 1298.
(   mathastext:   ) adding = ; and + to \nfss@catcodes
(   mathastext:   ) parentheses ( ) [ ] and slash /
(   mathastext:   ) alldelims: < > \backslash \setminus | \vert \mid \{
\}
LaTeX Font Info:    Redeclaring math symbol \setminus on input line 1364.
LaTeX Info: Redefining \models on input line 1383.
(   mathastext:   ) \# \mathdollar \% \&
(   mathastext:   ) \imath and \jmath
LaTeX Font Info:    Overwriting math alphabet `\Mathnormalbold' in
version `nor
mal'
(Font)                  T1/Merriwthr-OsF/b/it --> T1/Merriwthr-OsF/b/it
on inpu
t line 2863.
LaTeX Font Info:    Overwriting math alphabet `\Mathnormalbold' in
version `bol
d'
(Font)                  T1/Merriwthr-OsF/b/it --> T1/Merriwthr-OsF/b/it
on inpu
```

```
t line 2863.
LaTeX Font Info:    Overwriting symbol font `mtletterfont' in version
`normal'
(Font)                  T1/Merriwthr-OsF/m/it --> T1/Merriwthr-OsF/m/it
on inpu
t line 2863.
LaTeX Font Info:    Overwriting symbol font `mtletterfont' in version
`bold'
(Font)                  T1/Merriwthr-OsF/m/it --> T1/Merriwthr-OsF/b/it
on inpu
t line 2863.
LaTeX Font Info:    Overwriting symbol font `mtoperatorfont' in version
`normal
'
(Font)                  T1/Merriwthr-OsF/m/n --> T1/Merriwthr-OsF/m/n on
input
line 2863.
LaTeX Font Info:    Overwriting symbol font `mtoperatorfont' in version
`bold'
(Font)                  T1/Merriwthr-OsF/m/n --> T1/Merriwthr-OsF/b/n on
input
line 2863.
LaTeX Font Info:    Overwriting math alphabet `\Mathbf' in version
`normal'
(Font)                  T1/Merriwthr-OsF/b/n --> T1/Merriwthr-OsF/b/n on
input
line 2863.
LaTeX Font Info:    Overwriting math alphabet `\Mathbf' in version `bold'
(Font)                  T1/Merriwthr-OsF/b/n --> T1/Merriwthr-OsF/b/n on
input
line 2863.
LaTeX Font Info:    Overwriting math alphabet `\Mathit' in version
`normal'
(Font)                  T1/Merriwthr-OsF/m/it --> T1/Merriwthr-OsF/m/it
on inpu
t line 2863.
LaTeX Font Info:    Overwriting math alphabet `\Mathit' in version `bold'
(Font)                  T1/Merriwthr-OsF/m/it --> T1/Merriwthr-OsF/b/it
on inpu
t line 2863.
LaTeX Font Info:    Overwriting math alphabet `\Mathsf' in version
`normal'
(Font)                  T1/MerriwthrSans-OsF/m/n --> T1/MerriwthrSans-
OsF/m/n o
n input line 2863.
LaTeX Font Info:    Overwriting math alphabet `\Mathsf' in version `bold'
(Font)                  T1/MerriwthrSans-OsF/m/n --> T1/MerriwthrSans-
OsF/b/n o
n input line 2863.
LaTeX Font Info:    Overwriting math alphabet `\Mathtt' in version
`normal'
(Font)                  T1/lmtt/m/n --> T1/lmtt/m/n on input line 2863.
LaTeX Font Info:    Overwriting math alphabet `\Mathtt' in version `bold'
(Font)                  T1/lmtt/m/n --> T1/lmtt/b/n on input line 2863.
```

```
(   mathastext:   ) Latin letters in the `normal', resp. `bold',
(   mathastext:   ) math versions are now set up to use the fonts
(   mathastext:   ) T1/Merriwthr-OsF/m/it, resp. T1/Merriwthr-OsF/b/it.
(   mathastext:   ) Other characters (digits, ...) and \log-like names
will be
(   mathastext:   ) typeset with the n shape.
(   mathastext:   ) \hbar
(   mathastext:   ) minus as endash
(   mathastext:   ) The italic option is in effect.
(   mathastext:   ) \HUGE has been (re)-defined.
(   mathastext:   ) mathastext has declared larger sizes for subscripts.
(   mathastext:   ) To keep LaTeX defaults, use option
`defaultmathsizes'.

Package mathastext Info: Loading is complete.  You can now use
\Mathastext to
(mathastext)             modify the normal and bold math versions.  Use
it
(mathastext)             with optional argument or use \MTDeclareVersion
to
(mathastext)             declare additional math versions.
) (c:/texlive/2024/texmf-dist/tex/latex/relsize/relsize.sty
Package: relsize 2013/03/29 ver 4.1
) (c:/texlive/2024/texmf-dist/tex/latex/ragged2e/ragged2e.sty
Package: ragged2e 2023/06/22 v3.6 ragged2e Package
\CenteringLeftskip=\skip52
\RaggedLeftLeftskip=\skip53
\RaggedRightLeftskip=\skip54
\CenteringRightskip=\skip55
\RaggedLeftRightskip=\skip56
\RaggedRightRightskip=\skip57
\CenteringParfillskip=\skip58
\RaggedLeftParfillskip=\skip59
\RaggedRightParfillskip=\skip60
\JustifyingParfillskip=\skip61
\CenteringParindent=\skip62
\RaggedLeftParindent=\skip63
\RaggedRightParindent=\skip64
\JustifyingParindent=\skip65
) (c:/texlive/2024/texmf-dist/tex/latex/xcolor/xcolor.sty
Package: xcolor 2023/11/15 v3.01 LaTeX color extensions (UK)
(c:/texlive/2024/texmf-dist/tex/latex/graphics-cfg/color.cfg
File: color.cfg 2016/01/02 v1.6 sample color configuration
)
Package xcolor Info: Driver file: pdftex.def on input line 274.
(c:/texlive/2024/texmf-dist/tex/latex/graphics-def/pdftex.def
File: pdftex.def 2024/04/13 v1.2c Graphics/color driver for pdftex
) (c:/texlive/2024/texmf-dist/tex/latex/graphics/mathcolor.ltx)
Package xcolor Info: Model `cmy' substituted by `cmy0' on input line
1350.
Package xcolor Info: Model `hsb' substituted by `rgb' on input line 1354.
Package xcolor Info: Model `RGB' extended on input line 1366.
Package xcolor Info: Model `HTML' substituted by `rgb' on input line
1368.
```

```
Package xcolor Info: Model `Hsb' substituted by `hsb' on input line 1369.
Package xcolor Info: Model `tHsb' substituted by `hsb' on input line
1370.
Package xcolor Info: Model `HSB' substituted by `hsb' on input line 1371.
Package xcolor Info: Model `Gray' substituted by `gray' on input line
1372.
Package xcolor Info: Model `wave' substituted by `hsb' on input line
1373.
) (c:/texlive/2024/texmf-dist/tex/latex/colortbl/colortbl.sty
Package: colortbl 2024/07/06 v1.0i Color table columns (DPC)
(c:/texlive/2024/texmf-dist/tex/latex/tools/array.sty
Package: array 2024/06/14 v2.6d Tabular extension package (FMi)
\col@sep=\dimen143
\ar@mcellbox=\box53
\extrarowheight=\dimen144
\NC@list=\toks28
\extratabsurround=\skip66
\backup@length=\skip67
\ar@cellbox=\box54
)
\everycr=\toks29
\minrowclearance=\skip68
\rownum=\count272
) (c:/texlive/2024/texmf-dist/tex/latex/graphics/graphicx.sty
Package: graphicx 2021/09/16 v1.2d Enhanced LaTeX Graphics (DPC,SPQR)
(c:/texlive/2024/texmf-dist/tex/latex/graphics/graphics.sty
Package: graphics 2024/05/23 v1.4g Standard LaTeX Graphics (DPC,SPQR)
(c:/texlive/2024/texmf-dist/tex/latex/graphics/trig.sty
Package: trig 2023/12/02 v1.11 sin cos tan (DPC)
) (c:/texlive/2024/texmf-dist/tex/latex/graphics-cfg/graphics.cfg
File: graphics.cfg 2016/06/04 v1.11 sample graphics configuration
)
Package graphics Info: Driver file: pdftex.def on input line 106.
)
\Gin@req@height=\dimen145
\Gin@req@width=\dimen146
) (c:/texlive/2024/texmf-dist/tex/latex/xpatch/xpatch.sty
(c:/texlive/2024/texm
f-dist/tex/latex/l3kernel/expl3.sty
Package: expl3 2024-05-27 L3 programming layer (loader)
(c:/texlive/2024/texmf-dist/tex/latex/l3backend/l3backend-pdftex.def
File: l3backend-pdftex.def 2024-05-08 L3 backend support: PDF output
(pdfTeX)
\l__color_backend_stack_int=\count273
\l__pdf_internal_box=\box55
))
Package: xpatch 2020/03/25 v0.3a Extending etoolbox patching commands
(c:/texlive/2024/texmf-dist/tex/latex/l3packages/xparse/xparse.sty
Package: xparse 2024-05-08 L3 Experimental document command parser
)) (c:/texlive/2024/texmf-dist/tex/latex/environ/environ.sty
Package: environ 2014/05/04 v0.3 A new way to define environments
(c:/texlive/2024/texmf-dist/tex/latex/trimspaces/trimspaces.sty
Package: trimspaces 2009/09/17 v1.1 Trim spaces around a token list
)
```

```
\@envbody=\toks30
) (c:/texlive/2024/texmf-dist/tex/latex/lastpage/lastpage.sty
Package: lastpage 2024/07/07 v2.1c lastpage: 2.09 or 2e? (HMM)
(c:/texlive/2024/texmf-dist/tex/latex/lastpage/lastpage2e.sty
Package: lastpage2e 2024/07/07 v2.1c Decide which 2e lastpage version to
use (H
MM)
(c:/texlive/2024/texmf-dist/tex/latex/lastpage/lastpagemodern.sty
Package: lastpagemodern 2024-07-07 v2.1c Refers to last page's name (HMM;
JPG)
\c@lastpagecount=\count274
)
)) (c:/texlive/2024/texmf-dist/tex/latex/graphics/rotating.sty
Package: rotating 2016/08/11 v2.16d rotated objects in LaTeX
(c:/texlive/2024/texmf-dist/tex/latex/base/ifthen.sty
Package: ifthen 2024/03/16 v1.1e Standard LaTeX ifthen package (DPC)
)
\c@r@tfl@t=\count275
\rotFPtop=\skip69
\rotFPbot=\skip70
\rot@float@box=\box56
\rot@mess@toks=\toks31
) (c:/texlive/2024/texmf-dist/tex/latex/graphics/lscape.sty
Package: lscape 2020/05/28 v3.02 Landscape Pages (DPC)
) (c:/texlive/2024/texmf-dist/tex/latex/tools/afterpage.sty
Package: afterpage 2023/07/04 v1.08 After-Page Package (DPC)
\AP@output=\toks32
\AP@partial=\box57
\AP@footins=\box58
) (c:/texlive/2024/texmf-dist/tex/latex/textpos/textpos.sty
Package: textpos 2022/07/23 v1.10.1
Package textpos Info: choosing support for LaTeX3 on input line 60.
\TP@textbox=\box59
\TP@holdbox=\box60
\TPHorizModule=\dimen147
\TPVertModule=\dimen148
\TP@margin=\dimen149
\TP@absmargin=\dimen150
Grid set 16 x 16 = 37.34424pt x 52.81541pt
\TPboxrulesize=\dimen151
\TP@ox=\dimen152
\TP@oy=\dimen153
\TP@tbargs=\toks33
TextBlockOrigin set to 0pt x 0pt
) (c:/texlive/2024/texmf-dist/tex/latex/url/url.sty
\Urlmuskip=\muskip20
Package: url 2013/09/16  ver 3.4  Verb mode for urls, etc.
) (c:/texlive/2024/texmf-dist/tex/latex/newfloat/newfloat.sty
Package: newfloat 2023/10/01 v1.2 Defining new floating environments (AR)
Package newfloat Info: `rotating' package detected.
) (c:/texlive/2024/texmf-dist/tex/latex/mdframed/mdframed.sty
Package: mdframed 2013/07/01 1.9b: mdframed
(c:/texlive/2024/texmf-dist/tex/latex/kvoptions/kvoptions.sty
```

```
Package: kvoptions 2022-06-15 v3.15 Key value format for package options
(HO)
(c:/texlive/2024/texmf-dist/tex/generic/ltxcmds/ltxcmds.sty
Package: ltxcmds 2023-12-04 v1.26 LaTeX kernel commands for general use
(HO)
) (c:/texlive/2024/texmf-dist/tex/latex/kvsetkeys/kvsetkeys.sty
Package: kvsetkeys 2022-10-05 v1.19 Key value parser (HO)
)) (c:/texlive/2024/texmf-dist/tex/latex/zref/zref-abspage.sty
Package: zref-abspage 2023-09-14 v2.35 Module abspage for zref (HO)
(c:/texlive/2024/texmf-dist/tex/latex/zref/zref-base.sty
Package: zref-base 2023-09-14 v2.35 Module base for zref (HO)
(c:/texlive/2024/texmf-dist/tex/generic/infwarerr/infwarerr.sty
Package: infwarerr 2019/12/03 v1.5 Providing info/warning/error messages
(HO)
) (c:/texlive/2024/texmf-dist/tex/generic/kvdefinekeys/kvdefinekeys.sty
Package: kvdefinekeys 2019-12-19 v1.6 Define keys (HO)
) (c:/texlive/2024/texmf-dist/tex/generic/pdftexcmds/pdftexcmds.sty
Package: pdftexcmds 2020-06-27 v0.33 Utility functions of pdfTeX for
LuaTeX (HO
)
Package pdftexcmds Info: \pdf@primitive is available.
Package pdftexcmds Info: \pdf@ifprimitive is available.
Package pdftexcmds Info: \pdfdraftmode found.
) (c:/texlive/2024/texmf-dist/tex/generic/etexcmds/etexcmds.sty
Package: etexcmds 2019/12/15 v1.7 Avoid name clashes with e-TeX commands
(HO)
) (c:/texlive/2024/texmf-dist/tex/latex/auxhook/auxhook.sty
Package: auxhook 2019-12-17 v1.6 Hooks for auxiliary files (HO)
)
Package zref Info: New property list: main on input line 767.
Package zref Info: New property: default on input line 768.
Package zref Info: New property: page on input line 769.
)
\c@abspage=\count276
Package zref Info: New property: abspage on input line 67.
) (c:/texlive/2024/texmf-dist/tex/latex/needspace/needspace.sty
Package: needspace 2010/09/12 v1.3d reserve vertical space
)
\mdf@templength=\skip71
\c@mdf@globalstyle@cnt=\count277
\mdf@skipabove@length=\skip72
\mdf@skipbelow@length=\skip73
\mdf@leftmargin@length=\skip74
\mdf@rightmargin@length=\skip75
\mdf@innerleftmargin@length=\skip76
\mdf@innerrightmargin@length=\skip77
\mdf@innertopmargin@length=\skip78
\mdf@innerbottommargin@length=\skip79
\mdf@splittopskip@length=\skip80
\mdf@splitbottomskip@length=\skip81
\mdf@outermargin@length=\skip82
\mdf@innermargin@length=\skip83
\mdf@linewidth@length=\skip84
\mdf@innerlinewidth@length=\skip85
```

```
\mdf@middlelinewidth@length=\skip86
\mdf@outerlinewidth@length=\skip87
\mdf@roundcorner@length=\skip88
\mdf@footenotedistance@length=\skip89
\mdf@userdefinedwidth@length=\skip90
\mdf@needspace@length=\skip91
\mdf@frametitleaboveskip@length=\skip92
\mdf@frametitlebelowskip@length=\skip93
\mdf@frametitlerulewidth@length=\skip94
\mdf@frametitleleftmargin@length=\skip95
\mdf@frametitlerightmargin@length=\skip96
\mdf@shadowsize@length=\skip97
\mdf@extratopheight@length=\skip98
\mdf@subtitleabovelinewidth@length=\skip99
\mdf@subtitlebelowlinewidth@length=\skip100
\mdf@subtitleaboveskip@length=\skip101
\mdf@subtitlebelowskip@length=\skip102
\mdf@subtitleinneraboveskip@length=\skip103
\mdf@subtitleinnerbelowskip@length=\skip104
\mdf@subsubtitleabovelinewidth@length=\skip105
\mdf@subsubtitlebelowlinewidth@length=\skip106
\mdf@subsubtitleaboveskip@length=\skip107
\mdf@subsubtitlebelowskip@length=\skip108
\mdf@subsubtitleinneraboveskip@length=\skip109
\mdf@subsubtitleinnerbelowskip@length=\skip110
(c:/texlive/2024/texmf-dist/tex/latex/mdframed/md-frame-0.mdf
File: md-frame-0.mdf 2013/07/01\ 1.9b: md-frame-0
)
\mdf@frametitlebox=\box61
\mdf@footnotebox=\box62
\mdf@splitbox@one=\box63
\mdf@splitbox@two=\box64
\mdf@splitbox@save=\box65
\mdfsplitboxwidth=\skip111
\mdfsplitboxtotalwidth=\skip112
\mdfsplitboxheight=\skip113
\mdfsplitboxdepth=\skip114
\mdfsplitboxtotalheight=\skip115
\mdfframetitleboxwidth=\skip116
\mdfframetitleboxtotalwidth=\skip117
\mdfframetitleboxheight=\skip118
\mdfframetitleboxdepth=\skip119
\mdfframetitleboxtotalheight=\skip120
\mdffootnoteboxwidth=\skip121
\mdffootnoteboxtotalwidth=\skip122
\mdffootnoteboxheight=\skip123
\mdffootnoteboxdepth=\skip124
\mdffootnoteboxtotalheight=\skip125
\mdftotallinewidth=\skip126
\mdfboundingboxwidth=\skip127
\mdfboundingboxtotalwidth=\skip128
\mdfboundingboxheight=\skip129
\mdfboundingboxdepth=\skip130
\mdfboundingboxtotalheight=\skip131
```

```
\mdf@freevspace@length=\skip132
\mdf@horizontalwidthofbox@length=\skip133
\mdf@verticalmarginwhole@length=\skip134
\mdf@horizontalspaceofbox=\skip135
\mdfsubtitleheight=\skip136
\mdfsubsubtitleheight=\skip137
\c@mdfcountframes=\count278

****** mdframed patching \endmdf@trivlist

****** -- success******

\mdf@envdepth=\count279
\c@mdf@env@i=\count280
\c@mdf@env@ii=\count281
\c@mdf@zref@counter=\count282
Package zref Info: New property: mdf@pagevalue on input line 895.
) (c:/texlive/2024/texmf-dist/tex/latex/titlesec/titlesec.sty
Package: titlesec 2023/10/27 v2.16 Sectioning titles
\ttl@box=\box66
\beforetitleunit=\skip138
\aftertitleunit=\skip139
\ttl@plus=\dimen154
\ttl@minus=\dimen155
\ttl@toksa=\toks34
\titlewidth=\dimen156
\titlewidthlast=\dimen157
\titlewidthfirst=\dimen158
) (c:/texlive/2024/texmf-dist/tex/latex/koma-script/scrextend.sty
Package: scrextend 2023/07/07 v3.41 KOMA-Script package (extend other
classes w
ith features of KOMA-Script classes)
(c:/texlive/2024/texmf-dist/tex/latex/koma-script/scrkbase.sty
Package: scrkbase 2023/07/07 v3.41 KOMA-Script package (KOMA-Script-
dependent b
asics and keyval usage)
(c:/texlive/2024/texmf-dist/tex/latex/koma-script/scrbase.sty
Package: scrbase 2023/07/07 v3.41 KOMA-Script package (KOMA-Script-
independent
basics and keyval usage)
(c:/texlive/2024/texmf-dist/tex/latex/koma-script/scrlfile.sty
Package: scrlfile 2023/07/07 v3.41 KOMA-Script package (file load hooks)
(c:/texlive/2024/texmf-dist/tex/latex/koma-script/scrlfile-hook.sty
Package: scrlfile-hook 2023/07/07 v3.41 KOMA-Script package (using LaTeX
hooks)

(c:/texlive/2024/texmf-dist/tex/latex/koma-script/scrlogo.sty
Package: scrlogo 2023/07/07 v3.41 KOMA-Script package (logo)
)))
Applying: [2021/05/01] Usage of raw or classic option list on input line
252.
Already applied: [0000/00/00] Usage of raw or classic option list on
input line
 368.
```

```
))
Package scrextend Info: unexpected definition of `\@makefnmark'.
(scrextend)             Trying to patch it on input line 1762.
Package scrextend Info: patch seems to be successfull on input line 1762.
)

LaTeX Font Warning: Font shape `T1/cmr/m/n' in size <7.5> not available
(Font)              size <7> substituted on input line 69.

(c:/texlive/2024/texmf-dist/tex/latex/tools/calc.sty
Package: calc 2023/07/08 v4.3 Infix arithmetic (KKT,FJ)
\calc@Acount=\count283
\calc@Bcount=\count284
\calc@Adimen=\dimen159
\calc@Bdimen=\dimen160
\calc@Askip=\skip140
\calc@Bskip=\skip141
LaTeX Info: Redefining \setlength on input line 80.
LaTeX Info: Redefining \addtolength on input line 81.
\calc@Ccount=\count285
\calc@Cskip=\skip142
) (c:/texlive/2024/texmf-dist/tex/latex/geometry/geometry.sty
Package: geometry 2020/01/02 v5.9 Page Geometry
(c:/texlive/2024/texmf-dist/tex/generic/iftex/ifvtex.sty
Package: ifvtex 2019/10/25 v1.7 ifvtex legacy package. Use iftex instead.
)
\Gm@cnth=\count286
\Gm@cntv=\count287
\c@Gm@tempcnt=\count288
\Gm@bindingoffset=\dimen161
\Gm@wd@mp=\dimen162
\Gm@odd@mp=\dimen163
\Gm@even@mp=\dimen164
\Gm@layoutwidth=\dimen165
\Gm@layoutheight=\dimen166
\Gm@layouthoffset=\dimen167
\Gm@layoutvoffset=\dimen168
\Gm@dimlist=\toks35
) (c:/texlive/2024/texmf-dist/tex/latex/preprint/authblk.sty
Package: authblk 2001/02/27 1.3 (PWD)
\affilsep=\skip143
\@affilsep=\skip144
\c@Maxaffil=\count289
\c@authors=\count290
\c@affil=\count291
) (c:/texlive/2024/texmf-dist/tex/latex/footmisc/footmisc.sty
Package: footmisc 2023/07/05 v6.0f a miscellany of footnote facilities
\FN@temptoken=\toks36
\footnotemargin=\dimen169
\@outputbox@depth=\dimen170
Package footmisc Info: Declaring symbol style bringhurst on input line
696.
Package footmisc Info: Declaring symbol style chicago on input line 704.
Package footmisc Info: Declaring symbol style wiley on input line 713.
```

```
Package footmisc Info: Declaring symbol style lamport-robust on input
line 724.

Package footmisc Info: Declaring symbol style lamport* on input line 744.
Package footmisc Info: Declaring symbol style lamport*-robust on input
line 765
.
) (c:/texlive/2024/texmf-dist/tex/latex/fancyhdr/fancyhdr.sty
Package: fancyhdr 2024/07/23 v4.3.1 Extensive control of page headers and
foote
rs
\f@nch@headwidth=\skip145
\f@nch@O@elh=\skip146
\f@nch@O@erh=\skip147
\f@nch@O@olh=\skip148
\f@nch@O@orh=\skip149
\f@nch@O@elf=\skip150
\f@nch@O@erf=\skip151
\f@nch@O@olf=\skip152
\f@nch@O@orf=\skip153
) (c:/texlive/2024/texmf-dist/tex/generic/alphalph/alphalph.sty
Package: alphalph 2019/12/09 v2.6 Convert numbers to letters (HO)
(c:/texlive/2024/texmf-dist/tex/generic/intcalc/intcalc.sty
Package: intcalc 2019/12/15 v1.3 Expandable calculations with integers
(HO)
))
\c@authorfn=\count292
(c:/texlive/2024/texmf-dist/tex/latex/abstract/abstract.sty
Package: abstract 2009/06/08 v1.2a configurable abstracts
\abstitleskip=\skip154
\absleftindent=\skip155
\absrightindent=\skip156
\absparindent=\skip157
\absparsep=\skip158
)
Package newfloat Info: New float `keypoints' with options
`placement=t!,name=kp
t' on input line 291.
\c@keypoints=\count293
\newfloat@ftype=\count294
Package newfloat Info: float type `keypoints'=8 on input line 291.
(c:/texlive/2024/texmf-dist/tex/latex/enumitem/enumitem.sty
Package: enumitem 2019/06/20 v3.9 Customized lists
\labelindent=\skip159
\enit@outerparindent=\dimen171
\enit@toks=\toks37
\enit@inbox=\box67
\enit@count@id=\count295
\enitdp@description=\count296
) (c:/texlive/2024/texmf-dist/tex/latex/quoting/quoting.sty
Package: quoting 2014/01/28 v0.1c Consolidated environment for displayed
text
\quo@toppartop=\skip160
) (c:/texlive/2024/texmf-dist/tex/latex/sttools/stfloats.sty
```

```
Package: stfloats 2017/03/27 v3.3 Improve float mechanism and
baselineskip sett
ings
\@dblbotnum=\count297
\c@dblbotnumber=\count298
) (c:/texlive/2024/texmf-dist/tex/latex/booktabs/booktabs.sty
Package: booktabs 2020/01/12 v1.61803398 Publication quality tables
\heavyrulewidth=\dimen172
\lightrulewidth=\dimen173
\cmidrulewidth=\dimen174
\belowrulesep=\dimen175
\belowbottomsep=\dimen176
\aboverulesep=\dimen177
\abovetopsep=\dimen178
\cmidrulesep=\dimen179
\cmidrulekern=\dimen180
\defaultaddspace=\dimen181
\@cmidla=\count299
\@cmidlb=\count300
\@aboverulesep=\dimen182
\@belowrulesep=\dimen183
\@thisruleclass=\count301
\@lastruleclass=\count302
\@thisrulewidth=\dimen184
) (c:/texlive/2024/texmf-dist/tex/latex/tools/tabularx.sty
Package: tabularx 2023/12/11 v2.12a `tabularx' package (DPC)
\TX@col@width=\dimen185
\TX@old@table=\dimen186
\TX@old@col=\dimen187
\TX@target=\dimen188
\TX@delta=\dimen189
\TX@cols=\count303
\TX@ftn=\toks38
)
\enitdp@tablenotes=\count304
(c:/texlive/2024/texmf-dist/tex/latex/caption/caption.sty
Package: caption 2023/08/05 v3.6o Customizing captions (AR)
(c:/texlive/2024/texmf-dist/tex/latex/caption/caption3.sty
Package: caption3 2023/07/31 v2.4d caption3 kernel (AR)
\caption@tempdima=\dimen190
\captionmargin=\dimen191
\caption@leftmargin=\dimen192
\caption@rightmargin=\dimen193
\caption@width=\dimen194
\caption@indent=\dimen195
\caption@parindent=\dimen196
\caption@hangindent=\dimen197
Package caption Info: Standard document class detected.
)
\c@caption@flags=\count305
\c@continuedfloat=\count306
Package caption Info: rotating package is loaded.
Package caption Info: scrextend package is loaded.
\caption@addmargin@hsize=\dimen198
```

```
\caption@addmargin@linewidth=\dimen199
) (c:/texlive/2024/texmf-dist/tex/latex/natbib/natbib.sty
Package: natbib 2010/09/13 8.31b (PWD, AO)
\bibhang=\skip161
\bibsep=\skip162
LaTeX Info: Redefining \cite on input line 694.
\c@NAT@ctr=\count307
)) (c:/texlive/2024/texmf-dist/tex/latex/siunitx/siunitx.sty
Package: siunitx 2024-06-24 v3.3.19 A comprehensive (SI) units package
\l__siunitx_number_uncert_offset_int=\count308
\l__siunitx_number_exponent_fixed_int=\count309
\l__siunitx_number_min_decimal_int=\count310
\l__siunitx_number_min_integer_int=\count311
\l__siunitx_number_round_precision_int=\count312
\l__siunitx_number_lower_threshold_int=\count313
\l__siunitx_number_upper_threshold_int=\count314
\l__siunitx_number_group_first_int=\count315
\l__siunitx_number_group_size_int=\count316
\l__siunitx_number_group_minimum_int=\count317
\l__siunitx_angle_tmp_dim=\dimen256
\l__siunitx_angle_marker_box=\box68
\l__siunitx_angle_unit_box=\box69
\l__siunitx_compound_count_int=\count318
(c:/texlive/2024/texmf-dist/tex/latex/translations/translations.sty
Package: translations 2022/02/05 v1.12 internationalization of LaTeX2e
packages
 (CN)
) (c:/texlive/2024/texmf-dist/tex/latex/amsmath/amstext.sty
Package: amstext 2021/08/26 v2.01 AMS text
(c:/texlive/2024/texmf-dist/tex/latex/amsmath/amsgen.sty
File: amsgen.sty 1999/11/30 v2.0 generic functions
\@emptytoks=\toks39
\ex@=\dimen257
))
\l__siunitx_table_tmp_box=\box70
\l__siunitx_table_tmp_dim=\dimen258
\l__siunitx_table_column_width_dim=\dimen259
\l__siunitx_table_integer_box=\box71
\l__siunitx_table_decimal_box=\box72
\l__siunitx_table_uncert_box=\box73
\l__siunitx_table_before_box=\box74
\l__siunitx_table_after_box=\box75
\l__siunitx_table_before_dim=\dimen260
\l__siunitx_table_carry_dim=\dimen261
\l__siunitx_unit_tmp_int=\count319
\l__siunitx_unit_position_int=\count320
\l__siunitx_unit_total_int=\count321
) (c:/texlive/2024/texmf-dist/tex/latex/orcidlink/orcidlink.sty
Package: orcidlink 2024/06/26 v1.1.0 Support ORCID's three different ID
formats
.
(c:/texlive/2024/texmf-dist/tex/latex/hyperref/hyperref.sty
Package: hyperref 2024-07-10 v7.01j Hypertext links for LaTeX
(c:/texlive/2024/texmf-dist/tex/generic/pdfescape/pdfescape.sty
```

```
Package: pdfescape 2019/12/09 v1.15 Implements pdfTeX's escape features
(HO)
) (c:/texlive/2024/texmf-dist/tex/latex/hycolor/hycolor.sty
Package: hycolor 2020-01-27 v1.10 Color options for hyperref/bookmark
(HO)
) (c:/texlive/2024/texmf-dist/tex/latex/hyperref/nameref.sty
Package: nameref 2023-11-26 v2.56 Cross-referencing by name of section
(c:/texlive/2024/texmf-dist/tex/latex/refcount/refcount.sty
Package: refcount 2019/12/15 v3.6 Data extraction from label references
(HO)
) (c:/texlive/2024/texmf-
dist/tex/generic/gettitlestring/gettitlestring.sty
Package: gettitlestring 2019/12/15 v1.6 Cleanup title references (HO)
)
\c@section@level=\count322
) (c:/texlive/2024/texmf-dist/tex/generic/stringenc/stringenc.sty
Package: stringenc 2019/11/29 v1.12 Convert strings between diff.
encodings (HO
)
)
\@linkdim=\dimen262
\Hy@linkcounter=\count323
\Hy@pagecounter=\count324
(c:/texlive/2024/texmf-dist/tex/latex/hyperref/pd1enc.def
File: pd1enc.def 2024-07-10 v7.01j Hyperref: PDFDocEncoding definition
(HO)
Now handling font encoding PD1 ...
... no UTF-8 mapping file for font encoding PD1
)
\Hy@SavedSpaceFactor=\count325
(c:/texlive/2024/texmf-dist/tex/latex/hyperref/puenc.def
File: puenc.def 2024-07-10 v7.01j Hyperref: PDF Unicode definition (HO)
Now handling font encoding PU ...
... no UTF-8 mapping file for font encoding PU
)
Package hyperref Info: Hyper figures OFF on input line 4157.
Package hyperref Info: Link nesting OFF on input line 4162.
Package hyperref Info: Hyper index ON on input line 4165.
Package hyperref Info: Plain pages OFF on input line 4172.
Package hyperref Info: Backreferencing OFF on input line 4177.
Package hyperref Info: Implicit mode ON; LaTeX internals redefined.
Package hyperref Info: Bookmarks ON on input line 4424.
\c@Hy@tempcnt=\count326
LaTeX Info: Redefining \url on input line 4763.
\XeTeXLinkMargin=\dimen263
(c:/texlive/2024/texmf-dist/tex/generic/bitset/bitset.sty
Package: bitset 2019/12/09 v1.3 Handle bit-vector datatype (HO)
(c:/texlive/2024/texmf-dist/tex/generic/bigintcalc/bigintcalc.sty
Package: bigintcalc 2019/12/15 v1.5 Expandable calculations on big
integers (HO
)
))
\Fld@menulength=\count327
\Field@Width=\dimen264
```

```
\Fld@charsize=\dimen265
Package hyperref Info: Hyper figures OFF on input line 6042.
Package hyperref Info: Link nesting OFF on input line 6047.
Package hyperref Info: Hyper index ON on input line 6050.
Package hyperref Info: backreferencing OFF on input line 6057.
Package hyperref Info: Link coloring OFF on input line 6062.
Package hyperref Info: Link coloring with OCG OFF on input line 6067.
Package hyperref Info: PDF/A mode OFF on input line 6072.
(c:/texlive/2024/texmf-dist/tex/latex/base/atbegshi-ltx.sty
Package: atbegshi-ltx 2021/01/10 v1.0c Emulation of the original atbegshi
package with kernel methods
)
\Hy@abspage=\count328
\c@Item=\count329
\c@Hfootnote=\count330
)
Package hyperref Info: Driver (autodetected): hpdftex.
(c:/texlive/2024/texmf-dist/tex/latex/hyperref/hpdftex.def
File: hpdftex.def 2024-07-10 v7.01j Hyperref driver for pdfTeX
(c:/texlive/2024/texmf-dist/tex/latex/base/atveryend-ltx.sty
Package: atveryend-ltx 2020/08/19 v1.0a Emulation of the original
atveryend pac
kage
with kernel methods
)
\HyAnn@Count=\count331
\Fld@listcount=\count332
\c@bookmark@seq@number=\count333
(c:/texlive/2024/texmf-dist/tex/latex/rerunfilecheck/rerunfilecheck.sty
Package: rerunfilecheck 2022-07-10 v1.10 Rerun checks for auxiliary files
(HO)
(c:/texlive/2024/texmf-dist/tex/generic/uniquecounter/uniquecounter.sty
Package: uniquecounter 2019/12/15 v1.4 Provide unlimited unique counter
(HO)
)
Package uniquecounter Info: New unique counter `rerunfilecheck' on input
line 2
85.
)
\Hy@SectionHShift=\skip163
) (c:/texlive/2024/texmf-dist/tex/latex/pgf/frontendlayer/tikz.sty
(c:/texlive/
2024/texmf-dist/tex/latex/pgf/basiclayer/pgf.sty (c:/texlive/2024/texmf-
dist/te
x/latex/pgf/utilities/pgfrcs.sty (c:/texlive/2024/texmf-
dist/tex/generic/pgf/ut
ilities/pgfutil-common.tex
\pgfutil@everybye=\toks40
\pgfutil@tempdima=\dimen266
\pgfutil@tempdimb=\dimen267
) (c:/texlive/2024/texmf-dist/tex/generic/pgf/utilities/pgfutil-latex.def
\pgfutil@abb=\box76
) (c:/texlive/2024/texmf-dist/tex/generic/pgf/utilities/pgfrcs.code.tex
(c:/tex
```

```
live/2024/texmf-dist/tex/generic/pgf/pgf.revision.tex)
Package: pgfrcs 2023-01-15 v3.1.10 (3.1.10)
))
Package: pgf 2023-01-15 v3.1.10 (3.1.10)
(c:/texlive/2024/texmf-dist/tex/latex/pgf/basiclayer/pgfcore.sty
(c:/texlive/20
24/texmf-dist/tex/latex/pgf/systemlayer/pgfsys.sty
(c:/texlive/2024/texmf-dist/
tex/generic/pgf/systemlayer/pgfsys.code.tex
Package: pgfsys 2023-01-15 v3.1.10 (3.1.10)
(c:/texlive/2024/texmf-dist/tex/generic/pgf/utilities/pgfkeys.code.tex
\pgfkeys@pathtoks=\toks41
\pgfkeys@temptoks=\toks42

(c:/texlive/2024/texmf-
dist/tex/generic/pgf/utilities/pgfkeyslibraryfiltered.co
de.tex
\pgfkeys@tmptoks=\toks43
))
\pgf@x=\dimen268
\pgf@y=\dimen269
\pgf@xa=\dimen270
\pgf@ya=\dimen271
\pgf@xb=\dimen272
\pgf@yb=\dimen273
\pgf@xc=\dimen274
\pgf@yc=\dimen275
\pgf@xd=\dimen276
\pgf@yd=\dimen277
\w@pgf@writea=\write3
\r@pgf@reada=\read2
\c@pgf@counta=\count334
\c@pgf@countb=\count335
\c@pgf@countc=\count336
\c@pgf@countd=\count337
\t@pgf@toka=\toks44
\t@pgf@tokb=\toks45
\t@pgf@tokc=\toks46
\pgf@sys@id@count=\count338
(c:/texlive/2024/texmf-dist/tex/generic/pgf/systemlayer/pgf.cfg
File: pgf.cfg 2023-01-15 v3.1.10 (3.1.10)
)
Driver file for pgf: pgfsys-pdftex.def
(c:/texlive/2024/texmf-dist/tex/generic/pgf/systemlayer/pgfsys-pdftex.def
File: pgfsys-pdftex.def 2023-01-15 v3.1.10 (3.1.10)
(c:/texlive/2024/texmf-dist/tex/generic/pgf/systemlayer/pgfsys-common-
pdf.def
File: pgfsys-common-pdf.def 2023-01-15 v3.1.10 (3.1.10)
)))
(c:/texlive/2024/texmf-
dist/tex/generic/pgf/systemlayer/pgfsyssoftpath.code.tex
File: pgfsyssoftpath.code.tex 2023-01-15 v3.1.10 (3.1.10)
\pgfsyssoftpath@smallbuffer@items=\count339
\pgfsyssoftpath@bigbuffer@items=\count340
```

```
)
(c:/texlive/2024/texmf-
dist/tex/generic/pgf/systemlayer/pgfsysprotocol.code.tex
File: pgfsysprotocol.code.tex 2023-01-15 v3.1.10 (3.1.10)
)) (c:/texlive/2024/texmf-
dist/tex/generic/pgf/basiclayer/pgfcore.code.tex
Package: pgfcore 2023-01-15 v3.1.10 (3.1.10)
(c:/texlive/2024/texmf-dist/tex/generic/pgf/math/pgfmath.code.tex
(c:/texlive/2
024/texmf-dist/tex/generic/pgf/math/pgfmathutil.code.tex)
(c:/texlive/2024/texm
f-dist/tex/generic/pgf/math/pgfmathparser.code.tex
\pgfmath@dimen=\dimen278
\pgfmath@count=\count341
\pgfmath@box=\box77
\pgfmath@toks=\toks47
\pgfmath@stack@operand=\toks48
\pgfmath@stack@operation=\toks49
) (c:/texlive/2024/texmf-
dist/tex/generic/pgf/math/pgfmathfunctions.code.tex)
(c:/texlive/2024/texmf-
dist/tex/generic/pgf/math/pgfmathfunctions.basic.code.te
x)
(c:/texlive/2024/texmf-
dist/tex/generic/pgf/math/pgfmathfunctions.trigonometric
.code.tex)
(c:/texlive/2024/texmf-
dist/tex/generic/pgf/math/pgfmathfunctions.random.code.t
ex)
(c:/texlive/2024/texmf-
dist/tex/generic/pgf/math/pgfmathfunctions.comparison.co
de.tex)
(c:/texlive/2024/texmf-
dist/tex/generic/pgf/math/pgfmathfunctions.base.code.tex
)
(c:/texlive/2024/texmf-
dist/tex/generic/pgf/math/pgfmathfunctions.round.code.te
x)
(c:/texlive/2024/texmf-
dist/tex/generic/pgf/math/pgfmathfunctions.misc.code.tex
)
(c:/texlive/2024/texmf-
dist/tex/generic/pgf/math/pgfmathfunctions.integerarithm
etics.code.tex) (c:/texlive/2024/texmf-
dist/tex/generic/pgf/math/pgfmathcalc.co
de.tex) (c:/texlive/2024/texmf-
dist/tex/generic/pgf/math/pgfmathfloat.code.tex
\c@pgfmathroundto@lastzeros=\count342
)) (c:/texlive/2024/texmf-dist/tex/generic/pgf/math/pgfint.code.tex)
(c:/texliv
e/2024/texmf-dist/tex/generic/pgf/basiclayer/pgfcorepoints.code.tex
File: pgfcorepoints.code.tex 2023-01-15 v3.1.10 (3.1.10)
\pgf@picminx=\dimen279
\pgf@picmaxx=\dimen280
```

```
\pgf@picminy=\dimen281
\pgf@picmaxy=\dimen282
\pgf@pathminx=\dimen283
\pgf@pathmaxx=\dimen284
\pgf@pathminy=\dimen285
\pgf@pathmaxy=\dimen286
\pgf@xx=\dimen287
\pgf@xy=\dimen288
\pgf@yx=\dimen289
\pgf@yy=\dimen290
\pgf@zx=\dimen291
\pgf@zy=\dimen292
)
(c:/texlive/2024/texmf-
dist/tex/generic/pgf/basiclayer/pgfcorepathconstruct.cod
e.tex
File: pgfcorepathconstruct.code.tex 2023-01-15 v3.1.10 (3.1.10)
\pgf@path@lastx=\dimen293
\pgf@path@lasty=\dimen294
)
(c:/texlive/2024/texmf-
dist/tex/generic/pgf/basiclayer/pgfcorepathusage.code.te
x
File: pgfcorepathusage.code.tex 2023-01-15 v3.1.10 (3.1.10)
\pgf@shorten@end@additional=\dimen295
\pgf@shorten@start@additional=\dimen296
) (c:/texlive/2024/texmf-
dist/tex/generic/pgf/basiclayer/pgfcorescopes.code.tex
File: pgfcorescopes.code.tex 2023-01-15 v3.1.10 (3.1.10)
\pgfpic=\box78
\pgf@hbox=\box79
\pgf@layerbox@main=\box80
\pgf@picture@serial@count=\count343
)
(c:/texlive/2024/texmf-
dist/tex/generic/pgf/basiclayer/pgfcoregraphicstate.code
.tex
File: pgfcoregraphicstate.code.tex 2023-01-15 v3.1.10 (3.1.10)
\pgflinewidth=\dimen297
)
(c:/texlive/2024/texmf-
dist/tex/generic/pgf/basiclayer/pgfcoretransformations.c
ode.tex
File: pgfcoretransformations.code.tex 2023-01-15 v3.1.10 (3.1.10)
\pgf@pt@x=\dimen298
\pgf@pt@y=\dimen299
\pgf@pt@temp=\dimen300
) (c:/texlive/2024/texmf-
dist/tex/generic/pgf/basiclayer/pgfcorequick.code.tex
File: pgfcorequick.code.tex 2023-01-15 v3.1.10 (3.1.10)
) (c:/texlive/2024/texmf-
dist/tex/generic/pgf/basiclayer/pgfcoreobjects.code.te
x
File: pgfcoreobjects.code.tex 2023-01-15 v3.1.10 (3.1.10)
```

```
)
(c:/texlive/2024/texmf-
dist/tex/generic/pgf/basiclayer/pgfcorepathprocessing.co
de.tex
File: pgfcorepathprocessing.code.tex 2023-01-15 v3.1.10 (3.1.10)
) (c:/texlive/2024/texmf-
dist/tex/generic/pgf/basiclayer/pgfcorearrows.code.tex
File: pgfcorearrows.code.tex 2023-01-15 v3.1.10 (3.1.10)
\pgfarrowsep=\dimen301
) (c:/texlive/2024/texmf-
dist/tex/generic/pgf/basiclayer/pgfcoreshade.code.tex
File: pgfcoreshade.code.tex 2023-01-15 v3.1.10 (3.1.10)
\pgf@max=\dimen302
\pgf@sys@shading@range@num=\count344
\pgf@shadingcount=\count345
) (c:/texlive/2024/texmf-
dist/tex/generic/pgf/basiclayer/pgfcoreimage.code.tex
File: pgfcoreimage.code.tex 2023-01-15 v3.1.10 (3.1.10)
)
(c:/texlive/2024/texmf-
dist/tex/generic/pgf/basiclayer/pgfcoreexternal.code.tex
File: pgfcoreexternal.code.tex 2023-01-15 v3.1.10 (3.1.10)
\pgfexternal@startupbox=\box81
) (c:/texlive/2024/texmf-
dist/tex/generic/pgf/basiclayer/pgfcorelayers.code.tex
File: pgfcorelayers.code.tex 2023-01-15 v3.1.10 (3.1.10)
)
(c:/texlive/2024/texmf-
dist/tex/generic/pgf/basiclayer/pgfcoretransparency.code
.tex
File: pgfcoretransparency.code.tex 2023-01-15 v3.1.10 (3.1.10)
)
(c:/texlive/2024/texmf-
dist/tex/generic/pgf/basiclayer/pgfcorepatterns.code.tex
File: pgfcorepatterns.code.tex 2023-01-15 v3.1.10 (3.1.10)
) (c:/texlive/2024/texmf-
dist/tex/generic/pgf/basiclayer/pgfcorerdf.code.tex
File: pgfcorerdf.code.tex 2023-01-15 v3.1.10 (3.1.10)
))) (c:/texlive/2024/texmf-
dist/tex/generic/pgf/modules/pgfmoduleshapes.code.te
x
File: pgfmoduleshapes.code.tex 2023-01-15 v3.1.10 (3.1.10)
\pgfnodeparttextbox=\box82
) (c:/texlive/2024/texmf-
dist/tex/generic/pgf/modules/pgfmoduleplot.code.tex
File: pgfmoduleplot.code.tex 2023-01-15 v3.1.10 (3.1.10)
)
(c:/texlive/2024/texmf-dist/tex/latex/pgf/compatibility/pgfcomp-version-
0-65.st
y
Package: pgfcomp-version-0-65 2023-01-15 v3.1.10 (3.1.10)
\pgf@nodesepstart=\dimen303
\pgf@nodesepend=\dimen304
)
```

```
(c:/texlive/2024/texmf-dist/tex/latex/pgf/compatibility/pgfcomp-version-
1-18.st
y
Package: pgfcomp-version-1-18 2023-01-15 v3.1.10 (3.1.10)
)) (c:/texlive/2024/texmf-dist/tex/latex/pgf/utilities/pgffor.sty
(c:/texlive/2
024/texmf-dist/tex/latex/pgf/utilities/pgfkeys.sty
(c:/texlive/2024/texmf-dist/
tex/generic/pgf/utilities/pgfkeys.code.tex)) (c:/texlive/2024/texmf-
dist/tex/la
tex/pgf/math/pgfmath.sty (c:/texlive/2024/texmf-
dist/tex/generic/pgf/math/pgfma
th.code.tex)) (c:/texlive/2024/texmf-
dist/tex/generic/pgf/utilities/pgffor.code
.tex
Package: pgffor 2023-01-15 v3.1.10 (3.1.10)
\pgffor@iter=\dimen305
\pgffor@skip=\dimen306
\pgffor@stack=\toks50
\pgffor@toks=\toks51
)) (c:/texlive/2024/texmf-
dist/tex/generic/pgf/frontendlayer/tikz/tikz.code.tex
Package: tikz 2023-01-15 v3.1.10 (3.1.10)

(c:/texlive/2024/texmf-
dist/tex/generic/pgf/libraries/pgflibraryplothandlers.co
de.tex
File: pgflibraryplothandlers.code.tex 2023-01-15 v3.1.10 (3.1.10)
\pgf@plot@mark@count=\count346
\pgfplotmarksize=\dimen307
)
\tikz@lastx=\dimen308
\tikz@lasty=\dimen309
\tikz@lastxsaved=\dimen310
\tikz@lastysaved=\dimen311
\tikz@lastmovetox=\dimen312
\tikz@lastmovetoy=\dimen313
\tikzleveldistance=\dimen314
\tikzsiblingdistance=\dimen315
\tikz@figbox=\box83
\tikz@figbox@bg=\box84
\tikz@tempbox=\box85
\tikz@tempbox@bg=\box86
\tikztreelevel=\count347
\tikznumberofchildren=\count348
\tikznumberofcurrentchild=\count349
\tikz@fig@count=\count350
(c:/texlive/2024/texmf-
dist/tex/generic/pgf/modules/pgfmodulematrix.code.tex
File: pgfmodulematrix.code.tex 2023-01-15 v3.1.10 (3.1.10)
\pgfmatrixcurrentrow=\count351
\pgfmatrixcurrentcolumn=\count352
\pgf@matrix@numberofcolumns=\count353
)
```

```
\tikz@expandcount=\count354

(c:/texlive/2024/texmf-
dist/tex/generic/pgf/frontendlayer/tikz/libraries/tikzli
brarytopaths.code.tex
File: tikzlibrarytopaths.code.tex 2023-01-15 v3.1.10 (3.1.10)
)))
(c:/texlive/2024/texmf-
dist/tex/generic/pgf/frontendlayer/tikz/libraries/tikzli
brarysvg.path.code.tex
File: tikzlibrarysvg.path.code.tex 2023-01-15 v3.1.10 (3.1.10)

(c:/texlive/2024/texmf-
dist/tex/generic/pgf/libraries/pgflibrarysvg.path.code.t
ex
File: pgflibrarysvg.path.code.tex 2023-01-15 v3.1.10 (3.1.10)
(c:/texlive/2024/texmf-
dist/tex/generic/pgf/modules/pgfmoduleparser.code.tex
File: pgfmoduleparser.code.tex 2023-01-15 v3.1.10 (3.1.10)
\pgfparserdef@arg@count=\count355
)
\pgf@lib@svg@last@x=\dimen316
\pgf@lib@svg@last@y=\dimen317
\pgf@lib@svg@last@c@x=\dimen318
\pgf@lib@svg@last@c@y=\dimen319
\pgf@lib@svg@count=\count356
\pgf@lib@svg@max@num=\count357
))
\@curXheight=\skip164
)
Package hyperref Info: Option `unicode' set `true' on input line 48.
Package hyperref Info: Option `colorlinks' set `false' on input line 48.

! LaTeX Error: Option clash for package hyperref.

See the LaTeX manual or LaTeX Companion for explanation.
Type  H <return>  for immediate help.
 ...

l.50 \begin{document}

The package hyperref has already been loaded with options:
  []
There has now been an attempt to load it with options
  [colorlinks,allcolors=black,urlcolor=blue]
Adding the global options:
  ,colorlinks,allcolors=black,urlcolor=blue
to your \documentclass declaration may fix this.
Try typing  <return>  to proceed.

Package translations Info: No language package found. I am going to use
`englis
h' as default language. on input line 50.
```

```
LaTeX Font Info:    Trying to load font information for T1+Merriwthr-OsF
on inp
ut line 50.
(c:/texlive/2024/texmf-dist/tex/latex/merriweather/T1Merriwthr-OsF.fd
File: T1Merriwthr-OsF.fd 2020/08/30 (autoinst) Font definitions for
T1/Merriwth
r-OsF.
)
LaTeX Font Info:    Font shape `T1/Merriwthr-OsF/m/n' will be
(Font)              scaled to size 7.5pt on input line 50.
(./main.aux

LaTeX Warning: Label `subsubsec1' multiply defined.


LaTeX Warning: Label `subsubsec1' multiply defined.


LaTeX Warning: Label `subsubsec1' multiply defined.

)
\openout1 = `main.aux'.

LaTeX Font Info:    Checking defaults for OML/cmm/m/it on input line 50.
LaTeX Font Info:    ... okay on input line 50.
LaTeX Font Info:    Checking defaults for OMS/cmsy/m/n on input line 50.
LaTeX Font Info:    ... okay on input line 50.
LaTeX Font Info:    Checking defaults for OT1/cmr/m/n on input line 50.
LaTeX Font Info:    ... okay on input line 50.
LaTeX Font Info:    Checking defaults for T1/cmr/m/n on input line 50.
LaTeX Font Info:    ... okay on input line 50.
LaTeX Font Info:    Checking defaults for TS1/cmr/m/n on input line 50.
LaTeX Font Info:    ... okay on input line 50.
LaTeX Font Info:    Checking defaults for OMX/cmex/m/n on input line 50.
LaTeX Font Info:    ... okay on input line 50.
LaTeX Font Info:    Checking defaults for U/cmr/m/n on input line 50.
LaTeX Font Info:    ... okay on input line 50.
LaTeX Font Info:    Checking defaults for PD1/pdf/m/n on input line 50.
LaTeX Font Info:    ... okay on input line 50.
LaTeX Font Info:    Checking defaults for PU/pdf/m/n on input line 50.
LaTeX Font Info:    ... okay on input line 50.
LaTeX Info: Redefining \microtypecontext on input line 50.
Package microtype Info: Applying patch `item' on input line 50.
Package microtype Info: Applying patch `toc' on input line 50.
Package microtype Info: Applying patch `eqnum' on input line 50.
Package microtype Info: Applying patch `footnote' on input line 50.
Package microtype Info: Applying patch `verbatim' on input line 50.
Package microtype Info: Generating PDF output.
Package microtype Info: Character protrusion enabled (level 2).
Package microtype Info: Using default protrusion set `alltext'.
Package microtype Info: Automatic font expansion enabled (level 2),
(microtype)             stretch: 20, shrink: 20, step: 1, non-selected.
Package microtype Info: Using default expansion set `alltext-nott'.
LaTeX Info: Redefining \showhyphens on input line 50.
```

```
Package microtype Info: No adjustment of tracking.
Package microtype Info: No adjustment of interword spacing.
Package microtype Info: No adjustment of character kerning.
Package microtype Info: Loading generic protrusion settings for font
family
(microtype)             `Merriwthr-OsF' (encoding: T1).
(microtype)             For optimal results, create family-specific
settings.
(microtype)             See the microtype manual for details.
LaTeX Font Info:    Redeclaring symbol font `operators' on input line 50.
LaTeX Font Info:    Encoding `OT1' has changed to `T1' for symbol font
(Font)              `operators' in the math version `normal' on input
line 50.
LaTeX Font Info:    Overwriting symbol font `operators' in version
`normal'
(Font)                  OT1/cmr/m/n --> T1/Merriwthr-OsF/m/up on input
line 50.

LaTeX Font Info:    Encoding `OT1' has changed to `T1' for symbol font
(Font)              `operators' in the math version `bold' on input line
50.
LaTeX Font Info:    Overwriting symbol font `operators' in version `bold'
(Font)                  OT1/cmr/bx/n --> T1/Merriwthr-OsF/m/up on input
line 50
.
LaTeX Font Info:    Overwriting symbol font `operators' in version `bold'
(Font)                  T1/Merriwthr-OsF/m/up --> T1/Merriwthr-OsF/b/up
on inpu
t line 50.
LaTeX Font Info:    Redeclaring math alphabet \mathbf on input line 50.
LaTeX Font Info:    Overwriting math alphabet `\mathbf' in version
`normal'
(Font)                  OT1/cmr/bx/n --> T1/Merriwthr-OsF/b/up on input
line 50
.
LaTeX Font Info:    Overwriting math alphabet `\mathbf' in version `bold'
(Font)                  OT1/cmr/bx/n --> T1/Merriwthr-OsF/b/up on input
line 50
.
LaTeX Font Info:    Redeclaring math alphabet \mathsf on input line 50.
LaTeX Font Info:    Overwriting math alphabet `\mathsf' in version
`normal'
(Font)                  OT1/cmss/m/n --> T1/MerriwthrSans-OsF/m/up on
input lin
e 50.
LaTeX Font Info:    Overwriting math alphabet `\mathsf' in version `bold'
(Font)                  OT1/cmss/bx/n --> T1/MerriwthrSans-OsF/m/up on
input li
ne 50.
LaTeX Font Info:    Redeclaring math alphabet \mathit on input line 50.
LaTeX Font Info:    Overwriting math alphabet `\mathit' in version
`normal'
(Font)                  OT1/cmr/m/it --> T1/Merriwthr-OsF/m/it on input
line 50
```

```
.
LaTeX Font Info:    Overwriting math alphabet `\mathit' in version `bold'
(Font)                  OT1/cmr/bx/it --> T1/Merriwthr-OsF/m/it on input
line 5
0.
LaTeX Font Info:    Redeclaring math alphabet \mathtt on input line 50.
LaTeX Font Info:    Overwriting math alphabet `\mathtt' in version
`normal'
(Font)                  OT1/cmtt/m/n --> T1/lmtt/m/up on input line 50.
LaTeX Font Info:    Overwriting math alphabet `\mathtt' in version `bold'
(Font)                  OT1/cmtt/m/n --> T1/lmtt/m/up on input line 50.
LaTeX Font Info:    Overwriting math alphabet `\mathsf' in version `bold'
(Font)                  T1/MerriwthrSans-OsF/m/up --> T1/MerriwthrSans-
OsF/b/up
 on input line 50.
LaTeX Font Info:    Overwriting math alphabet `\mathit' in version `bold'
(Font)                  T1/Merriwthr-OsF/m/it --> T1/Merriwthr-OsF/b/it
on inpu
t line 50.
\c@mv@tabular=\count358
\c@mv@boldtabular=\count359
(c:/texlive/2024/texmf-dist/tex/context/base/mkii/supp-pdf.mkii
[Loading MPS to PDF converter (version 2006.09.02).]
\scratchcounter=\count360
\scratchdimen=\dimen320
\scratchbox=\box87
\nofMPsegments=\count361
\nofMParguments=\count362
\everyMPshowfont=\toks52
\MPscratchCnt=\count363
\MPscratchDim=\dimen321
\MPnumerator=\count364
\makeMPintoPDFobject=\count365
\everyMPtoPDFconversion=\toks53
) (c:/texlive/2024/texmf-dist/tex/latex/epstopdf-pkg/epstopdf-base.sty
Package: epstopdf-base 2020-01-24 v2.11 Base part for package epstopdf
Package epstopdf-base Info: Redefining graphics rule for `.eps' on input
line 4
85.
(c:/texlive/2024/texmf-dist/tex/latex/latexconfig/epstopdf-sys.cfg
File: epstopdf-sys.cfg 2010/07/13 v1.3 Configuration of (r)epstopdf for
TeX Liv
e
))
*geometry* driver: auto-detecting
*geometry* detected driver: pdftex
*geometry* verbose mode - [ preamble ] result:
* driver: pdftex
* paper: a4paper
* layout: <same size as paper>
* layoutoffset:(h,v)=(0.0pt,0.0pt)
* modes: includefoot twoside
* h-part:(L,W,R)=(54.64pt, 488.22787pt, 54.64pt)
* v-part:(T,H,B)=(66.0pt, 745.04684pt, 34.0pt)
```

```
* \paperwidth=597.50787pt
* \paperheight=845.04684pt
* \textwidth=488.22787pt
* \textheight=715.04684pt
* \oddsidemargin=-17.62999pt
* \evensidemargin=-17.62999pt
* \topmargin=-47.76999pt
* \headheight=17.5pt
* \headsep=24.0pt
* \topskip=10.0pt
* \footskip=30.0pt
* \marginparwidth=48.0pt
* \marginparsep=10.0pt
* \columnsep=18.0pt
* \skip\footins=22.0pt plus 2.0pt
* \hoffset=0.0pt
* \voffset=0.0pt
* \mag=1000
* \@twocolumntrue
* \@twosidetrue
* \@mparswitchtrue
* \@reversemarginfalse
* (1in=72.27pt=25.4mm, 1cm=28.453pt)

Package caption Info: Begin \AtBeginDocument code.
Package caption Info: hyperref package is loaded.
Package caption Info: End \AtBeginDocument code.

(c:/texlive/2024/texmf-dist/tex/latex/translations/translations-basic-
dictionar
y-english.trsl
File: translations-basic-dictionary-english.trsl (english translation
file `tra
nslations-basic-dictionary')
)
Package translations Info: loading dictionary `translations-basic-
dictionary' f
or `english'. on input line 50.
Package hyperref Info: Link coloring OFF on input line 50.
(./main.out) (./main.out)
\@outlinefile=\write4
\openout4 = `main.out'.

\@gscitedetails=\box88
\@gscitedetailsheight=\skip165
\@gsheadbox=\box89
\@gsheadboxheight=\skip166
LaTeX Font Info:    Font shape `T1/Merriwthr-OsF/b/n' will be
(Font)              scaled to size 6.5pt on input line 50.
LaTeX Font Info:    Calculating math sizes for size <7.5> on input line
50.

LaTeX Font Warning: Font shape `T1/Merriwthr-OsF/m/up' undefined
```

```
(Font)              using `T1/Merriwthr-OsF/m/n' instead on input line
50.

LaTeX Font Info:    Font shape `T1/Merriwthr-OsF/m/up' will be
(Font)              scaled to size 6.24973pt on input line 50.
LaTeX Font Info:    Font shape `T1/Merriwthr-OsF/m/up' will be
(Font)              scaled to size 5.24997pt on input line 50.
LaTeX Font Info:    Trying to load font information for U+eur on input
line 50.

(c:/texlive/2024/texmf-dist/tex/latex/amsfonts/ueur.fd
File: ueur.fd 2013/01/14 v3.01 Euler Roman
) (c:/texlive/2024/texmf-dist/tex/latex/microtype/mt-eur.cfg
File: mt-eur.cfg 2006/07/31 v1.1 microtype config. file: AMS Euler Roman
(RS)
)

LaTeX Font Warning: Font shape `OMS/cmsy/m/n' in size <7.5> not available
(Font)              size <7> substituted on input line 50.

LaTeX Font Info:    External font `cmex10' loaded for size
(Font)              <7.5> on input line 50.
LaTeX Font Info:    External font `cmex10' loaded for size
(Font)              <6.24973> on input line 50.
LaTeX Font Info:    External font `cmex10' loaded for size
(Font)              <5.24997> on input line 50.
LaTeX Font Info:    Trying to load font information for U+euf on input
line 50.

(c:/texlive/2024/texmf-dist/tex/latex/amsfonts/ueuf.fd
File: ueuf.fd 2013/01/14 v3.01 Euler Fraktur
) (c:/texlive/2024/texmf-dist/tex/latex/microtype/mt-euf.cfg
File: mt-euf.cfg 2006/07/03 v1.1 microtype config. file: AMS Euler
Fraktur (RS)

)
LaTeX Font Info:    Trying to load font information for U+eus on input
line 50.

(c:/texlive/2024/texmf-dist/tex/latex/amsfonts/ueus.fd
File: ueus.fd 2013/01/14 v3.01 Euler Script
) (c:/texlive/2024/texmf-dist/tex/latex/microtype/mt-eus.cfg
File: mt-eus.cfg 2006/07/28 v1.2 microtype config. file: AMS Euler Script
(RS)
)
LaTeX Font Info:    Trying to load font information for U+euex on input
line 50
.
(c:/texlive/2024/texmf-dist/tex/latex/amsfonts/ueuex.fd
File: ueuex.fd 2013/01/14 v3.01 Euler extra symbols
)

LaTeX Font Warning: Font shape `OML/cmm/m/it' in size <7.5> not available
(Font)              size <7> substituted on input line 50.
```

```
LaTeX Font Info:    Font shape `T1/Merriwthr-OsF/m/n' will be
(Font)              scaled to size 6.24973pt on input line 50.
LaTeX Font Info:    Font shape `T1/Merriwthr-OsF/m/n' will be
(Font)              scaled to size 5.24997pt on input line 50.
LaTeX Font Info:    Font shape `T1/Merriwthr-OsF/m/it' will be
(Font)              scaled to size 7.5pt on input line 50.
LaTeX Font Info:    Font shape `T1/Merriwthr-OsF/m/it' will be
(Font)              scaled to size 6.24973pt on input line 50.
LaTeX Font Info:    Font shape `T1/Merriwthr-OsF/m/it' will be
(Font)              scaled to size 5.24997pt on input line 50.
LaTeX Font Info:    Font shape `T1/Merriwthr-OsF/m/n' will be
(Font)              scaled to size 8.0pt on input line 50.
LaTeX Font Info:    Font shape `T1/Merriwthr-OsF/m/it' will be
(Font)              scaled to size 8.0pt on input line 50.
LaTeX Font Info:    Font shape `T1/Merriwthr-OsF/b/it' will be
(Font)              scaled to size 8.0pt on input line 50.
TextBlockOrigin set to 4pc+6.64pt x 4pc+6pt
<gigascience-logo.pdf, id=124, 99.37125pt x 33.12375pt>
File: gigascience-logo.pdf Graphic file (type pdf)
<use gigascience-logo.pdf>
Package pdftex.def Info: gigascience-logo.pdf  used on input line 66.
(pdftex.def)             Requested size: 126.00902pt x 42.0pt.

Overfull \hbox (54.64pt too wide) in paragraph at lines 66--66
[][]
 []

LaTeX Font Info:    Font shape `T1/Merriwthr-OsF/m/n' will be
(Font)              scaled to size 14.0pt on input line 66.
LaTeX Font Info:    Font shape `T1/Merriwthr-OsF/m/n' will be
(Font)              scaled to size 8.99997pt on input line 66.
LaTeX Font Info:    Calculating math sizes for size <14> on input line
66.
LaTeX Font Info:    Font shape `T1/Merriwthr-OsF/m/up' will be
(Font)              scaled to size 14.0pt on input line 66.
LaTeX Font Info:    Font shape `T1/Merriwthr-OsF/m/up' will be
(Font)              scaled to size 11.66617pt on input line 66.
LaTeX Font Info:    Font shape `T1/Merriwthr-OsF/m/up' will be
(Font)              scaled to size 9.79996pt on input line 66.
LaTeX Font Info:    External font `cmex10' loaded for size
(Font)              <14> on input line 66.
LaTeX Font Info:    External font `cmex10' loaded for size
(Font)              <11.66617> on input line 66.
LaTeX Font Info:    External font `cmex10' loaded for size
(Font)              <9.79996> on input line 66.
LaTeX Font Info:    Font shape `T1/Merriwthr-OsF/m/n' will be
(Font)              scaled to size 11.66617pt on input line 66.
LaTeX Font Info:    Font shape `T1/Merriwthr-OsF/m/n' will be
(Font)              scaled to size 9.79996pt on input line 66.
LaTeX Font Info:    Font shape `T1/Merriwthr-OsF/m/it' will be
(Font)              scaled to size 14.0pt on input line 66.
LaTeX Font Info:    Font shape `T1/Merriwthr-OsF/m/it' will be
(Font)              scaled to size 11.66617pt on input line 66.
```

```
LaTeX Font Info:    Font shape `T1/Merriwthr-OsF/m/it' will be
(Font)              scaled to size 9.79996pt on input line 66.
LaTeX Font Info:    Font shape `T1/Merriwthr-OsF/b/n' will be
(Font)              scaled to size 18.0pt on input line 66.
LaTeX Font Info:    Font shape `T1/Merriwthr-OsF/m/n' will be
(Font)              scaled to size 13.0pt on input line 66.
LaTeX Font Info:    Calculating math sizes for size <13> on input line
66.
LaTeX Font Info:    Font shape `T1/Merriwthr-OsF/m/up' will be
(Font)              scaled to size 13.0pt on input line 66.
LaTeX Font Info:    Font shape `T1/Merriwthr-OsF/m/up' will be
(Font)              scaled to size 10.83287pt on input line 66.
LaTeX Font Info:    Font shape `T1/Merriwthr-OsF/m/up' will be
(Font)              scaled to size 9.09996pt on input line 66.

LaTeX Font Warning: Font shape `OMS/cmsy/m/n' in size <13> not available
(Font)              size <12> substituted on input line 66.

LaTeX Font Info:    External font `cmex10' loaded for size
(Font)              <13> on input line 66.
LaTeX Font Info:    External font `cmex10' loaded for size
(Font)              <10.83287> on input line 66.
LaTeX Font Info:    External font `cmex10' loaded for size
(Font)              <9.09996> on input line 66.

LaTeX Font Warning: Font shape `OML/cmm/m/it' in size <13> not available
(Font)              size <12> substituted on input line 66.

LaTeX Font Info:    Font shape `T1/Merriwthr-OsF/m/n' will be
(Font)              scaled to size 10.83287pt on input line 66.
LaTeX Font Info:    Font shape `T1/Merriwthr-OsF/m/n' will be
(Font)              scaled to size 9.09996pt on input line 66.
LaTeX Font Info:    Font shape `T1/Merriwthr-OsF/m/it' will be
(Font)              scaled to size 13.0pt on input line 66.
LaTeX Font Info:    Font shape `T1/Merriwthr-OsF/m/it' will be
(Font)              scaled to size 10.83287pt on input line 66.
LaTeX Font Info:    Font shape `T1/Merriwthr-OsF/m/it' will be
(Font)              scaled to size 9.09996pt on input line 66.
LaTeX Font Info:    Trying to load font information for TS1+Merriwthr-OsF
on in
put line 66.
(c:/texlive/2024/texmf-dist/tex/latex/merriweather/TS1Merriwthr-OsF.fd
File: TS1Merriwthr-OsF.fd 2020/08/30 (autoinst) Font definitions for
TS1/Merriw
thr-OsF.
)
LaTeX Font Info:    Font shape `TS1/Merriwthr-OsF/m/n' will be
(Font)              scaled to size 10.83287pt on input line 66.
Package microtype Info: Loading generic protrusion settings for font
family
(microtype)             `Merriwthr-OsF' (encoding: TS1).
(microtype)             For optimal results, create family-specific
settings.
(microtype)             See the microtype manual for details.
```

```
LaTeX Font Info:    Font shape `T1/Merriwthr-OsF/m/n' will be
(Font)              scaled to size 9.0pt on input line 66.
LaTeX Font Info:    Font shape `T1/Merriwthr-OsF/m/up' will be
(Font)              scaled to size 9.0pt on input line 66.
LaTeX Font Info:    Font shape `T1/Merriwthr-OsF/m/up' will be
(Font)              scaled to size 7.0pt on input line 66.
LaTeX Font Info:    Font shape `T1/Merriwthr-OsF/m/up' will be
(Font)              scaled to size 5.0pt on input line 66.
LaTeX Font Info:    External font `cmex10' loaded for size
(Font)              <9> on input line 66.
LaTeX Font Info:    External font `cmex10' loaded for size
(Font)              <7> on input line 66.
LaTeX Font Info:    External font `cmex10' loaded for size
(Font)              <5> on input line 66.
LaTeX Font Info:    Font shape `T1/Merriwthr-OsF/m/n' will be
(Font)              scaled to size 7.0pt on input line 66.
LaTeX Font Info:    Font shape `T1/Merriwthr-OsF/m/n' will be
(Font)              scaled to size 5.0pt on input line 66.
LaTeX Font Info:    Font shape `T1/Merriwthr-OsF/m/it' will be
(Font)              scaled to size 9.0pt on input line 66.
LaTeX Font Info:    Font shape `T1/Merriwthr-OsF/m/it' will be
(Font)              scaled to size 7.0pt on input line 66.
LaTeX Font Info:    Font shape `T1/Merriwthr-OsF/m/it' will be
(Font)              scaled to size 5.0pt on input line 66.
LaTeX Font Info:    Font shape `T1/Merriwthr-OsF/m/n' will be
(Font)              scaled to size 6.5pt on input line 66.
LaTeX Font Info:    Calculating math sizes for size <6.5> on input line
66.
LaTeX Font Info:    Font shape `T1/Merriwthr-OsF/m/up' will be
(Font)              scaled to size 6.5pt on input line 66.
LaTeX Font Info:    Font shape `T1/Merriwthr-OsF/m/up' will be
(Font)              scaled to size 5.41643pt on input line 66.
LaTeX Font Info:    Font shape `T1/Merriwthr-OsF/m/up' will be
(Font)              scaled to size 4.54997pt on input line 66.

LaTeX Font Warning: Font shape `OMS/cmsy/m/n' in size <6.5> not available
(Font)              size <6> substituted on input line 66.


LaTeX Font Warning: Font shape `OMS/cmsy/m/n' in size <5.41643> not
available
(Font)              size <5> substituted on input line 66.


LaTeX Font Warning: Font shape `OMS/cmsy/m/n' in size <4.54997> not
available
(Font)              size <5> substituted on input line 66.

LaTeX Font Info:    External font `cmex10' loaded for size
(Font)              <6.5> on input line 66.
LaTeX Font Info:    External font `cmex10' loaded for size
(Font)              <5.41643> on input line 66.
LaTeX Font Info:    External font `cmex10' loaded for size
(Font)              <4.54997> on input line 66.
```

```
LaTeX Font Warning: Font shape `OML/cmm/m/it' in size <6.5> not available
(Font)              size <6> substituted on input line 66.


LaTeX Font Warning: Font shape `OML/cmm/m/it' in size <5.41643> not
available
(Font)              size <5> substituted on input line 66.


LaTeX Font Warning: Font shape `OML/cmm/m/it' in size <4.54997> not
available
(Font)              size <5> substituted on input line 66.

LaTeX Font Info:    Font shape `T1/Merriwthr-OsF/m/n' will be
(Font)              scaled to size 5.41643pt on input line 66.
LaTeX Font Info:    Font shape `T1/Merriwthr-OsF/m/n' will be
(Font)              scaled to size 4.54997pt on input line 66.
LaTeX Font Info:    Font shape `T1/Merriwthr-OsF/m/it' will be
(Font)              scaled to size 6.5pt on input line 66.
LaTeX Font Info:    Font shape `T1/Merriwthr-OsF/m/it' will be
(Font)              scaled to size 5.41643pt on input line 66.
LaTeX Font Info:    Font shape `T1/Merriwthr-OsF/m/it' will be
(Font)              scaled to size 4.54997pt on input line 66.
LaTeX Font Info:    Font shape `TS1/Merriwthr-OsF/m/n' will be
(Font)              scaled to size 5.41643pt on input line 66.

Overfull \hbox (54.64pt too wide) in paragraph at lines 66--66
[][][]
 []

LaTeX Font Info:    Font shape `T1/Merriwthr-OsF/b/n' will be
(Font)              scaled to size 10.0pt on input line 66.
LaTeX Font Info:    Font shape `T1/Merriwthr-OsF/b/n' will be
(Font)              scaled to size 8.0pt on input line 66.

Overfull \hbox (54.64pt too wide) in paragraph at lines 66--66
[][][]
 []

LaTeX Font Info:    Font shape `T1/Merriwthr-OsF/b/n' will be
(Font)              scaled to size 7.5pt on input line 73.

Package natbib Warning: Citation `ref1' on page 1 undefined on input line
73.


Package natbib Warning: Citation `ref2' on page 1 undefined on input line
73.


Package natbib Warning: Citation `ref3' on page 1 undefined on input line
73.
```

```
Package natbib Warning: Citation `ref4' on page 1 undefined on input line
73.


Underfull \vbox (badness 10000) has occurred while \output is active []




Underfull \vbox (badness 10000) has occurred while \output is active []



LaTeX Font Info:    Font shape `T1/Merriwthr-OsF/m/n' will be
(Font)              scaled to size 7.8pt on input line 77.
LaTeX Font Info:    Font shape `T1/Merriwthr-OsF/b/n' will be
(Font)              scaled to size 7.8pt on input line 77.
[1{c:/texlive/2024/texmf-
var/fonts/map/pdftex/updmap/pdftex.map}{c:/texlive/202
4/texmf-
dist/fonts/enc/dvips/merriweather/merriwthr_posqbl.enc}{c:/texlive/2024
/texmf-dist/fonts/enc/dvips/merriweather/merriwthr_owzwzj.enc}


 <./gigascience-logo.pdf>]

Package natbib Warning: Citation `ref5' on page 2 undefined on input line
79.


Package natbib Warning: Citation `ref6' on page 2 undefined on input line
79.


Underfull \hbox (badness 1616) in paragraph at lines 79--80
\T1/Merriwthr-OsF/m/n/7.5 (+20) mi-croR-NAs (miR-NAs) have been ex-ten-
sively s
tud-ied and are
 []


Package natbib Warning: Citation `ref11' on page 2 undefined on input
line 81.


Package natbib Warning: Citation `ref12' on page 2 undefined on input
line 81.


Package natbib Warning: Citation `ref7' on page 2 undefined on input line
83.
```

```
Package natbib Warning: Citation `ref8' on page 2 undefined on input line
83.


Package natbib Warning: Citation `ref9' on page 2 undefined on input line
85.


Package natbib Warning: Citation `ref10' on page 2 undefined on input
line 85.


Underfull \hbox (badness 1424) in paragraph at lines 87--88
[]\T1/Merriwthr-OsF/m/n/7.5 (+20) Despite their crit-i-cal roles in gene
reg-u-
la-tion and dis-ease
 []


Underfull \hbox (badness 1478) in paragraph at lines 87--88
\T1/Merriwthr-OsF/m/n/7.5 (+20) mech-a-nisms, ex-per-i-men-tal iden-ti-
fi-ca-ti
on of non-coding RNA
 []


Package natbib Warning: Citation `IMCMDA' on page 2 undefined on input
line 89.



Package natbib Warning: Citation `LncDisAP' on page 2 undefined on input
line 8
9.


Package natbib Warning: Citation `IPiDA-GBNN' on page 2 undefined on
input line
 89.


Package natbib Warning: Citation `GCNCDA' on page 2 undefined on input
line 89.



Package natbib Warning: Citation `CRBPSA' on page 2 undefined on input
line 89.



Package natbib Warning: Citation `StackCirRNAPred' on page 2 undefined on
input
 line 89.
```

Package natbib Warning: Citation `ref13' on page 2 undefined on input
line 95.

Package natbib Warning: Citation `ref14' on page 2 undefined on input
line 95.

Package natbib Warning: Citation `ref15' on page 2 undefined on input
line 95.

Package natbib Warning: Citation `ref16' on page 2 undefined on input
line 95.

Package natbib Warning: Citation `IMCMDA' on page 2 undefined on input
line 97.

Package natbib Warning: Citation `LncDisAP' on page 2 undefined on input
line 9
7.

Package natbib Warning: Citation `IPiDA-GBNN' on page 2 undefined on
input line
 97.

Package natbib Warning: Citation `GCNCDA' on page 2 undefined on input
line 97.

Underfull \hbox (badness 1199) in paragraph at lines 99--100
\T1/Merriwthr-OsF/m/n/7.5 (+20) var-i-ous ncRNA types. For in-stance,
miR-NAs p
ri-mar-ily func-
 []

Package natbib Warning: Citation `ref17' on page 2 undefined on input
line 106.

Underfull \hbox (badness 7099) in paragraph at lines 105--107

```
[]\T1/Merriwthr-OsF/m/n/7.5 (+20) In this pa-per, we pro-pose
\T1/Merriwthr-OsF
/b/n/7.5 (+20) Pan-GIA \T1/Merriwthr-OsF/m/n/7.5 (+20) (Pan-ncRNA Graph-
 []



LaTeX Font Info:    Font shape `T1/Merriwthr-OsF/m/it' will be
(Font)              scaled to size 7.8pt on input line 107.
[2]
LaTeX Font Info:    Font shape `TS1/Merriwthr-OsF/m/n' will be
(Font)              scaled to size 7.5pt on input line 110.

Package natbib Warning: Citation `ref18' on page 3 undefined on input
line 124.



Package natbib Warning: Citation `ref19' on page 3 undefined on input
line 124.



Package natbib Warning: Citation `ref20' on page 3 undefined on input
line 125.



Package natbib Warning: Citation `ref21' on page 3 undefined on input
line 125.



Package natbib Warning: Citation `ref22' on page 3 undefined on input
line 125.



Package natbib Warning: Citation `ref23' on page 3 undefined on input
line 125.



Package natbib Warning: Citation `ref15' on page 3 undefined on input
line 126.



Package natbib Warning: Citation `ref24' on page 3 undefined on input
line 126.
```

```
Package natbib Warning: Citation `piuco_pirnadb:_2021' on page 3
undefined on i
nput line 126.


Package natbib Warning: Citation `ref25' on page 3 undefined on input
line 127.



Underfull \hbox (badness 2189) in paragraph at lines 127--128
[]\T1/Merriwthr-OsF/b/n/7.5 (+20) Disease: \T1/Merriwthr-OsF/m/n/7.5
(+20) This
 study uti-lizes Dis-ease On-tol-ogy Iden-ti-fiers
 []



pdfTeX warning: pdflatex.exe (file ./architecture.pdf): PDF inclusion:
found PD
F version <1.7>, but at most version <1.5> allowed
<architecture.pdf, id=146, 512.14456pt x 707.84372pt>
File: architecture.pdf Graphic file (type pdf)
<use architecture.pdf>
Package pdftex.def Info: architecture.pdf  used on input line 134.
(pdftex.def)             Requested size: 488.22787pt x 674.8023pt.
LaTeX Font Info:    Font shape `T1/Merriwthr-OsF/m/n' will be
(Font)              scaled to size 6.0pt on input line 135.
LaTeX Font Info:    Font shape `T1/Merriwthr-OsF/b/n' will be
(Font)              scaled to size 6.0pt on input line 135.

! Package enumitem Error: (A) undefined.

See the enumitem package documentation for explanation.
Type  H <return>  for immediate help.
 ...

l.142 \begin{enumerate}[(A)]

Try typing  <return>  to proceed.
If that doesn't work, type  X <return>  to quit.

LaTeX Font Info:    Font shape `T1/Merriwthr-OsF/b/n' will be
(Font)              scaled to size 8.5pt on input line 148.

Package natbib Warning: Citation `ji_dnabert:_2021' on page 3 undefined
on inpu
t line 149.



LaTeX Font Info:    Font shape `T1/Merriwthr-OsF/b/sl' in size <7.5> not
availa
ble
```

```
(Font)              Font shape `T1/Merriwthr-OsF/b/it' tried instead on
input l
ine 150.
LaTeX Font Info:    Font shape `T1/Merriwthr-OsF/b/it' will be
(Font)              scaled to size 7.5pt on input line 150.

Package natbib Warning: Citation `ji_dnabert:_2021' on page 3 undefined
on inpu
t line 151.


Package natbib Warning: Citation `sanabria_dna_2024' on page 3 undefined
on inp
ut line 151.


Package natbib Warning: Citation `suzuki_genomic_2025' on page 3
undefined on i
nput line 151.

LaTeX Font Info:    Font shape `T1/Merriwthr-OsF/m/up' will be
(Font)              scaled to size 7.5pt on input line 154.
LaTeX Font Info:    Font shape `T1/Merriwthr-OsF/b/n' will be
(Font)              scaled to size 6.24973pt on input line 170.
LaTeX Font Info:    Font shape `T1/Merriwthr-OsF/b/n' will be
(Font)              scaled to size 5.24997pt on input line 170.


[3] [4 <./architecture.pdf>]
Underfull \hbox (badness 10000) in paragraph at lines 200--202
\T1/Merriwthr-OsF/m/up/7.5 (+20) where $\T1/Merriwthr-OsF/m/it/7.5 (+20)
A \OMS
/cmsy/m/n/7 2 \T1/Merriwthr-OsF/m/up/7.5 (+20) {0, 1}[]$ rep-re-sents the
known

 []

! Undefined control sequence.
l.209     S^{\text{RNA}} \in \mathbb
                                    {R}^{|\mathcal{V}_{\text{RNA}}|
\times |...
The control sequence at the end of the top line
of your error message was never \def'ed. If you have
misspelled it (e.g., `\hobx'), type `I' and the correct
spelling (e.g., `I\hbox'). Otherwise just continue,
and I'll forget about whatever was undefined.



! Undefined control sequence.
l.219     S^{\text{dis}} \in \mathbb
                                    {R}^{|\mathcal{V}_{\text{dis}}|
\times |...
The control sequence at the end of the top line
```

```
of your error message was never \def'ed. If you have
misspelled it (e.g., `\hobx'), type `I' and the correct
spelling (e.g., `I\hbox'). Otherwise just continue,
and I'll forget about whatever was undefined.

! Undefined control sequence.
l.225 ...mbedding vector $\mathbf{h}_i \in \mathbb
                                                  {R}^d$ using a
pretrained ...
The control sequence at the end of the top line
of your error message was never \def'ed. If you have
misspelled it (e.g., `\hobx'), type `I' and the correct
spelling (e.g., `I\hbox'). Otherwise just continue,
and I'll forget about whatever was undefined.


! LaTeX Error: Environment bmatrix undefined.

See the LaTeX manual or LaTeX Companion for explanation.
Type  H <return>  for immediate help.
 ...

l.235 \begin{bmatrix}

Your command was ignored.
Type  I <command> <return>  to replace it with another command,
or  <return>  to continue without it.

! Misplaced alignment tab character &.
l.236 S^{\text{seq}}_{\text{mi}} &
                                   0 & 0 & 0 \\
I can't figure out why you would want to use a tab mark
here. If you just want an ampersand, the remedy is
simple: Just type `I\&' now. But if some right brace
up above has ended a previous alignment prematurely,
you're probably due for more error messages, and you
might try typing `S' now just to see what is salvageable.

! Misplaced alignment tab character &.
l.236 S^{\text{seq}}_{\text{mi}} & 0 &
                                       0 & 0 \\
I can't figure out why you would want to use a tab mark
here. If you just want an ampersand, the remedy is
simple: Just type `I\&' now. But if some right brace
up above has ended a previous alignment prematurely,
you're probably due for more error messages, and you
might try typing `S' now just to see what is salvageable.

! Misplaced alignment tab character &.
l.236 S^{\text{seq}}_{\text{mi}} & 0 & 0 &
                                           0 \\
I can't figure out why you would want to use a tab mark
here. If you just want an ampersand, the remedy is
simple: Just type `I\&' now. But if some right brace
```

```
up above has ended a previous alignment prematurely,
you're probably due for more error messages, and you
might try typing `S' now just to see what is salvageable.

! Misplaced alignment tab character &.
l.237 0 &
          S^{\text{seq}}_{\text{circ}} & 0 & 0 \\
I can't figure out why you would want to use a tab mark
here. If you just want an ampersand, the remedy is
simple: Just type `I\&' now. But if some right brace
up above has ended a previous alignment prematurely,
you're probably due for more error messages, and you
might try typing `S' now just to see what is salvageable.

! Misplaced alignment tab character &.
l.237 0 & S^{\text{seq}}_{\text{circ}} &
                                          0 & 0 \\
I can't figure out why you would want to use a tab mark
here. If you just want an ampersand, the remedy is
simple: Just type `I\&' now. But if some right brace
up above has ended a previous alignment prematurely,
you're probably due for more error messages, and you
might try typing `S' now just to see what is salvageable.

! Misplaced alignment tab character &.
l.237 0 & S^{\text{seq}}_{\text{circ}} & 0 &
                                              0 \\
I can't figure out why you would want to use a tab mark
here. If you just want an ampersand, the remedy is
simple: Just type `I\&' now. But if some right brace
up above has ended a previous alignment prematurely,
you're probably due for more error messages, and you
might try typing `S' now just to see what is salvageable.

! Misplaced alignment tab character &.
l.238 0 &
          0 & S^{\text{seq}}_{\text{lnc}} & 0 \\
I can't figure out why you would want to use a tab mark
here. If you just want an ampersand, the remedy is
simple: Just type `I\&' now. But if some right brace
up above has ended a previous alignment prematurely,
you're probably due for more error messages, and you
might try typing `S' now just to see what is salvageable.

! Misplaced alignment tab character &.
l.238 0 & 0 &
              S^{\text{seq}}_{\text{lnc}} & 0 \\
I can't figure out why you would want to use a tab mark
here. If you just want an ampersand, the remedy is
simple: Just type `I\&' now. But if some right brace
up above has ended a previous alignment prematurely,
you're probably due for more error messages, and you
might try typing `S' now just to see what is salvageable.
```

```
! Misplaced alignment tab character &.
l.238 0 & 0 & S^{\text{seq}}_{\text{lnc}} &
                                             0 \\
I can't figure out why you would want to use a tab mark
here. If you just want an ampersand, the remedy is
simple: Just type `I\&' now. But if some right brace
up above has ended a previous alignment prematurely,
you're probably due for more error messages, and you
might try typing `S' now just to see what is salvageable.

! Misplaced alignment tab character &.
l.239 0 &
          0 & 0 & S^{\text{seq}}_{\text{pi}}
I can't figure out why you would want to use a tab mark
here. If you just want an ampersand, the remedy is
simple: Just type `I\&' now. But if some right brace
up above has ended a previous alignment prematurely,
you're probably due for more error messages, and you
might try typing `S' now just to see what is salvageable.

! Misplaced alignment tab character &.
l.239 0 & 0 &
              0 & S^{\text{seq}}_{\text{pi}}
I can't figure out why you would want to use a tab mark
here. If you just want an ampersand, the remedy is
simple: Just type `I\&' now. But if some right brace
up above has ended a previous alignment prematurely,
you're probably due for more error messages, and you
might try typing `S' now just to see what is salvageable.

! Misplaced alignment tab character &.
l.239 0 & 0 & 0 &
                  S^{\text{seq}}_{\text{pi}}
I can't figure out why you would want to use a tab mark
here. If you just want an ampersand, the remedy is
simple: Just type `I\&' now. But if some right brace
up above has ended a previous alignment prematurely,
you're probably due for more error messages, and you
might try typing `S' now just to see what is salvageable.


! LaTeX Error: \begin{equation} on input line 233 ended by \end{bmatrix}.

See the LaTeX manual or LaTeX Companion for explanation.
Type  H <return>  for immediate help.
 ...

l.240 \end{bmatrix}
                   ,
Your command was ignored.
Type  I <command> <return>  to replace it with another command,
or  <return>  to continue without it.
```

```
Package natbib Warning: Citation `ref30' on page 5 undefined on input
line 262.


Package natbib Warning: Citation `ref31' on page 5 undefined on input
line 262.


Package natbib Warning: Citation `ref32' on page 5 undefined on input
line 262.


[5]
! Undefined control sequence.
l.291 ...ix \( \mathbf{X}^{\text{rna}} \in \mathbb
                                                  {R}^{N_r \times d_r} \)
an...
The control sequence at the end of the top line
of your error message was never \def'ed. If you have
misspelled it (e.g., `\hobx'), type `I' and the correct
spelling (e.g., `I\hbox'). Otherwise just continue,
and I'll forget about whatever was undefined.

! Undefined control sequence.
l.291 ...ix \( \mathbf{X}^{\text{dis}} \in \mathbb
                                                  {R}^{N_d \times d_d}
\), w...
The control sequence at the end of the top line
of your error message was never \def'ed. If you have
misspelled it (e.g., `\hobx'), type `I' and the correct
spelling (e.g., `I\hbox'). Otherwise just continue,
and I'll forget about whatever was undefined.

! Undefined control sequence.
l.293 ...{X}^{\text{dis}} \mathbf{W}_d \in \mathbb
                                                  {R}^{N_d \times d_r}
The control sequence at the end of the top line
of your error message was never \def'ed. If you have
misspelled it (e.g., `\hobx'), type `I' and the correct
spelling (e.g., `I\hbox'). Otherwise just continue,
and I'll forget about whatever was undefined.


! Undefined control sequence.
l.295 Where \( \mathbf{W}_d \in \mathbb
                                       {R}^{d_d \times d_r} \) is a
learnabl...
The control sequence at the end of the top line
of your error message was never \def'ed. If you have
```

```
misspelled it (e.g., `\hobx'), type `I' and the correct
spelling (e.g., `I\hbox'). Otherwise just continue,
and I'll forget about whatever was undefined.

! Undefined control sequence.
l.301 ...na}}, \mathbf{H}^{\text{dis}} \in \mathbb
                                                  {R}^{N \times d_h} \)
repr...
The control sequence at the end of the top line
of your error message was never \def'ed. If you have
misspelled it (e.g., `\hobx'), type `I' and the correct
spelling (e.g., `I\hbox'). Otherwise just continue,
and I'll forget about whatever was undefined.

! Undefined control sequence.
l.305 ..._d} \mathbf{H}^{\text{dis}}_i \in \mathbb
                                                  {R}^{d_h}
The control sequence at the end of the top line
of your error message was never \def'ed. If you have
misspelled it (e.g., `\hobx'), type `I' and the correct
spelling (e.g., `I\hbox'). Otherwise just continue,
and I'll forget about whatever was undefined.

! Undefined control sequence.
l.310 ...thbf{H}}^{\text{dis}} \right] \in \mathbb
                                                  {R}^{N_r \times 2d_h}
The control sequence at the end of the top line
of your error message was never \def'ed. If you have
misspelled it (e.g., `\hobx'), type `I' and the correct
spelling (e.g., `I\hbox'). Otherwise just continue,
and I'll forget about whatever was undefined.

! Undefined control sequence.
l.320 ...s, \mathcal{E}_K(\mathbf{F})) \in \mathbb
                                                  {R}^{N_r \times K
\times d_e}
The control sequence at the end of the top line
of your error message was never \def'ed. If you have
misspelled it (e.g., `\hobx'), type `I' and the correct
spelling (e.g., `I\hbox'). Otherwise just continue,
and I'll forget about whatever was undefined.

! Undefined control sequence.
l.332 ... \mathbf{K}_t, \mathbf{V}_t)) \in \mathbb
                                                  {R}^{n_t \times K}
The control sequence at the end of the top line
of your error message was never \def'ed. If you have
misspelled it (e.g., `\hobx'), type `I' and the correct
spelling (e.g., `I\hbox'). Otherwise just continue,
and I'll forget about whatever was undefined.

! Undefined control sequence.
l.337 ...\mathbf{E}_{\mathcal{I}_t, k} \in \mathbb
                                                  {R}^{n_t \times d_e}
```

```
The control sequence at the end of the top line
of your error message was never \def'ed. If you have
misspelled it (e.g., `\hobx'), type `I' and the correct
spelling (e.g., `I\hbox'). Otherwise just continue,
and I'll forget about whatever was undefined.

! Undefined control sequence.
l.343 ...1, \dots, \bar{\mathbf{u}}_T] \in \mathbb
                                                  {R}^{T \times d_e},
\quad ...
The control sequence at the end of the top line
of your error message was never \def'ed. If you have
misspelled it (e.g., `\hobx'), type `I' and the correct
spelling (e.g., `I\hbox'). Otherwise just continue,
and I'll forget about whatever was undefined.



[6]
! Undefined control sequence.
l.346 ...f{U}, \mathbf{U}, \mathbf{U}) \in \mathbb
                                                  {R}^{T \times d_e}
The control sequence at the end of the top line
of your error message was never \def'ed. If you have
misspelled it (e.g., `\hobx'), type `I' and the correct
spelling (e.g., `I\hbox'). Otherwise just continue,
and I'll forget about whatever was undefined.

! Undefined control sequence.
l.351 ...f{u}_t \,\|\, \mathbf{U}'_t]) \in \mathbb
                                                  {R}^{n_t \times d_e}
The control sequence at the end of the top line
of your error message was never \def'ed. If you have
misspelled it (e.g., `\hobx'), type `I' and the correct
spelling (e.g., `I\hbox'). Otherwise just continue,
and I'll forget about whatever was undefined.

! Undefined control sequence.
l.357 ...is}} \mathbf{W}_{\text{proj}} \in \mathbb
                                                  {R}^{N_d \times d_e}
The control sequence at the end of the top line
of your error message was never \def'ed. If you have
misspelled it (e.g., `\hobx'), type `I' and the correct
spelling (e.g., `I\hbox'). Otherwise just continue,
and I'll forget about whatever was undefined.

! Undefined control sequence.
l.362 ...mathbf{H}}^{\text{dis}^\top}) \in \mathbb
                                                  {R}^{n_t \times N_d}
The control sequence at the end of the top line
of your error message was never \def'ed. If you have
misspelled it (e.g., `\hobx'), type `I' and the correct
spelling (e.g., `I\hbox'). Otherwise just continue,
and I'll forget about whatever was undefined.
```

```
LaTeX Font Info:    Font shape `T1/Merriwthr-OsF/b/n' will be
(Font)              scaled to size 7.0pt on input line 396.

Package natbib Warning: Citation `NIMGSA' on page 7 undefined on input
line 404
.


Package natbib Warning: Citation `MINIMDA' on page 7 undefined on input
line 40
5.


Package natbib Warning: Citation `gGATLDA' on page 7 undefined on input
line 40
8.


Package natbib Warning: Citation `LDGRNMF' on page 7 undefined on input
line 40
9.


Package natbib Warning: Citation `iPiDi-PUL' on page 7 undefined on input
line
412.


Package natbib Warning: Citation `PUTransGCN' on page 7 undefined on
input line
 413.


Package natbib Warning: Citation `IGNSCDA' on page 7 undefined on input
line 41
6.


Package natbib Warning: Citation `GATCL2CD' on page 7 undefined on input
line 4
17.

<bar.pdf, id=356, 381.89778pt x 331.58308pt>
File: bar.pdf Graphic file (type pdf)
<use bar.pdf>
Package pdftex.def Info: bar.pdf  used on input line 436.
(pdftex.def)             Requested size: 217.0pt x 188.41235pt.


[7 <./bar.pdf>]
<radar.pdf, id=389, 855.13177pt x 626.79268pt>
```

```
File: radar.pdf Graphic file (type pdf)
<use radar.pdf>
Package pdftex.def Info: radar.pdf  used on input line 445.
(pdftex.def)             Requested size: 217.0pt x 159.05054pt.
<AUC.pdf, id=393, 578.16pt x 433.62pt>
File: AUC.pdf Graphic file (type pdf)
<use AUC.pdf>
Package pdftex.def Info: AUC.pdf  used on input line 460.
(pdftex.def)             Requested size: 217.0pt x 162.74605pt.
<AUPR.pdf, id=394, 578.16pt x 433.62pt>
File: AUPR.pdf Graphic file (type pdf)
<use AUPR.pdf>
Package pdftex.def Info: AUPR.pdf  used on input line 466.
(pdftex.def)             Requested size: 217.0pt x 162.74605pt.
<Rank_Index.pdf, id=395, 578.16pt x 433.62pt>
File: Rank_Index.pdf Graphic file (type pdf)
<use Rank_Index.pdf>
Package pdftex.def Info: Rank_Index.pdf  used on input line 472.
(pdftex.def)             Requested size: 217.0pt x 162.74605pt.

Underfull \vbox (badness 1688) has occurred while \output is active []




[8 <./radar.pdf> <./AUC.pdf> <./AUPR.pdf>]

Package natbib Warning: Citation `34233294' on page 9 undefined on input
line 5
17.


Package natbib Warning: Citation `29218238' on page 9 undefined on input
line 5
17.


Package natbib Warning: Citation `35474736' on page 9 undefined on input
line 5
17.


Package natbib Warning: Citation `32185303' on page 9 undefined on input
line 5
17.


Underfull \hbox (badness 10000) in paragraph at lines 517--518
[]\T1/Merriwthr-OsF/m/up/7.5 (+20) In this case study, we fo-cused on
high-conf
idence
 []
```

```
Underfull \hbox (badness 2384) in paragraph at lines 517--518
\T1/Merriwthr-OsF/m/up/7.5 (+20) miRNA^^Udisease as-so-ci-a-tions pre-
dicted by
 our model. Lit-er-
 []


Underfull \hbox (badness 3861) in paragraph at lines 517--518
\T1/Merriwthr-OsF/m/up/7.5 (+20) ex-pres-sion, fur-ther sup-press-ing
AKT/ERK s
ig-nal-ing ac-tiv-ity,
 []


Underfull \hbox (badness 1748) in paragraph at lines 517--518
\T1/Merriwthr-OsF/m/up/7.5 (+20) block-ing the cell cy-cle and sup-press-
ing tu
-mor growth [\T1/Merriwthr-OsF/b/n/7.5 (+20) ? \T1/Merriwthr-OsF/m/up/7.5
(+20)
 ].
 []


Underfull \hbox (badness 1371) in paragraph at lines 517--518
\T1/Merriwthr-OsF/m/up/7.5 (+20) In os-teoarthri-tis (OA), over-ex-pres-
sion of
 miR-378 ag-gra-vates
 []


Underfull \hbox (badness 2932) in paragraph at lines 517--518
\T1/Merriwthr-OsF/m/up/7.5 (+20) and in-hibit-ing chon-dro-genic dif-fer-
en-ti-
a-tion of bone mar-row
 []


Underfull \hbox (badness 7221) in paragraph at lines 517--518
\T1/Merriwthr-OsF/m/up/7.5 (+20) al-le-vi-ates OA pro-gres-sion and pro-
motes j
oint re-gen-er-a-tion,
 []




Package natbib Warning: Citation `32495924' on page 9 undefined on input
line 5
19.


Package natbib Warning: Citation `39289188' on page 9 undefined on input
line 5
```

```
19.


Package natbib Warning: Citation `34236817' on page 9 undefined on input
line 5
19.


Package natbib Warning: Citation `31821324' on page 9 undefined on input
line 5
19.


Package natbib Warning: Citation `32855634' on page 9 undefined on input
line 5
21.


Package natbib Warning: Citation `32330554' on page 9 undefined on input
line 5
21.


Package natbib Warning: Citation `35322746' on page 9 undefined on input
line 5
21.


Package natbib Warning: Citation `28938565' on page 9 undefined on input
line 5
21.


Underfull \vbox (badness 10000) has occurred while \output is active []



[9 <./Rank_Index.pdf>]

Package natbib Warning: Citation `25998508' on page 10 undefined on input
line
523.


Package natbib Warning: Citation `29986767' on page 10 undefined on input
line
523.


Package natbib Warning: Citation `28127595' on page 10 undefined on input
line
523.
```

```
Underfull \hbox (badness 10000) in paragraph at lines 529--530
\T1/Merriwthr-OsF/m/up/7.5 (+20) In this study, we pro-posed
\T1/Merriwthr-OsF/
b/n/7.5 (+20) Pan-GIA\T1/Merriwthr-OsF/m/up/7.5 (+20) , a novel model for
 []


Underfull \hbox (badness 6526) in paragraph at lines 529--530
\T1/Merriwthr-OsF/m/up/7.5 (+20) ncRNA^^Udisease as-so-ci-a-tion pre-dic-
tion t
hat in-te-grates a Hi-
 []


Underfull \hbox (badness 2680) in paragraph at lines 529--530
\T1/Merriwthr-OsF/m/up/7.5 (+20) ar-chi-tec-ture. Com-pre-hen-sive ex-
per-i-men
ts demon-strate that
 []


Underfull \hbox (badness 2119) in paragraph at lines 529--530
\T1/Merriwthr-OsF/m/up/7.5 (+20) RNA types, in-clud-ing miRNA, lncRNA,
cir-cRNA
, and piRNA,
 []

LaTeX Font Info:    Trying to load font information for T1+lmtt on input
line 5
41.
(c:/texlive/2024/texmf-dist/tex/latex/lm/t1lmtt.fd
File: t1lmtt.fd 2015/05/01 v1.6.1 Font defs for Latin Modern
)
Package microtype Info: Loading generic protrusion settings for font
family
(microtype)             `lmtt' (encoding: T1).
(microtype)             For optimal results, create family-specific
settings.
(microtype)             See the microtype manual for details.

Underfull \hbox (badness 10000) in paragraph at lines 541--542
[]\T1/Merriwthr-OsF/m/up/7.5 (+20) Project home-page: []$\T1/lmtt/m/n/7.5
https
 : / / github . com / qiankunzizairen /
 []




Underfull \hbox (badness 10000) in paragraph at lines 549--550
[]\T1/Merriwthr-OsF/m/up/7.5 (+20) DOME-DL: []$\T1/lmtt/m/n/7.5 https : /
/ reg
istry . dome-[]ml . org / review /
 []
```

```
Package natbib Warning: Citation `liu_pangia_2023' on page 10 undefined
on inpu
t line 559.

No file main.bbl.

Package natbib Warning: There were undefined citations.



[10{c:/texlive/2024/texmf-dist/fonts/enc/dvips/lm/lm-ec.enc}]
enddocument/afterlastpage: lastpage setting LastPage.
(./main.aux)
 ***********
LaTeX2e <2024-06-01> patch level 2
L3 programming layer <2020/03/25>
 ***********

LaTeX Font Warning: Size substitutions with differences
(Font)              up to 1.0pt have occurred.


LaTeX Font Warning: Some font shapes were not available, defaults
substituted.


LaTeX Warning: There were multiply-defined labels.

Package rerunfilecheck Info: File `main.out' has not changed.
(rerunfilecheck)             Checksum:
667FDB9A678D454D2F69FEADF155C298;6919.
 )
Here is how much of TeX's memory you used:
 34633 strings out of 473583
 704004 string characters out of 5732343
 1977908 words of memory out of 5000000
 56248 multiletter control sequences out of 15000+600000
 1836371 words of font info for 540 fonts, out of 8000000 for 9000
 1141 hyphenation exceptions out of 8191
 123i,13n,131p,1553b,957s stack positions out of
10000i,1000n,20000p,200000b,200000s
<c:/texlive/2024/texmf-dist/fonts/type1/sorkin/merriweather/Merriwthr-
Bold.pf
b><c:/texlive/2024/texmf-dist/fonts/type1/sorkin/merriweather/Merriwthr-
BoldIta
lic.pfb><c:/texlive/2024/texmf-
dist/fonts/type1/sorkin/merriweather/Merriwthr-I
talic.pfb><c:/texlive/2024/texmf-
dist/fonts/type1/sorkin/merriweather/Merriwthr
-Regular.pfb><c:/texlive/2024/texmf-
dist/fonts/type1/public/amsfonts/cm/cmex10.
```

pfb><c:/texlive/2024/texmf-
dist/fonts/type1/public/amsfonts/cm/cmsy5.pfb><c:/te
xlive/2024/texmf-
dist/fonts/type1/public/amsfonts/cm/cmsy6.pfb><c:/texlive/2024
/texmf-
dist/fonts/type1/public/amsfonts/cm/cmsy7.pfb><c:/texlive/2024/texmf-dis
t/fonts/type1/public/amsfonts/euler/euex8.pfb><c:/texlive/2024/texmf-
dist/fonts
/type1/public/amsfonts/euler/eufm7.pfb><c:/texlive/2024/texmf-
dist/fonts/type1/
public/amsfonts/euler/eurm7.pfb><c:/texlive/2024/texmf-
dist/fonts/type1/public/
lm/lmtt8.pfb>
Output written on main.pdf (10 pages, 878380 bytes).
PDF statistics:
 650 PDF objects out of 1000 (max. 8388607)
 412 compressed objects within 5 object streams
 83 named destinations out of 1000 (max. 500000)
 203544 words of extra memory for PDF output out of 221844 (max.
10000000)

Manuscript Click here to access/download;Manuscript;PanGIA(1).pdf

Placeholder for OUP logo oup.pdf

(GIGA)^n SCIENCE

GigaScience, 2023, 1–12

doi: xx.xxxx/xxxx
Manuscript in Preparation
Paper

PAPER

PanGIA: A universal framework for identifying association between ncRNAs and diseases

Xiaoyuan Liu[1,†], Xiye Lü[1,†], Qiuhao Chen[2], Jiqiu Sun[4], Tianyi Zhao[1,2,*] and Yan Zhu[3,*]

[1]School of Medicine and Health, Harbin Institute of Technology, Xidazhi Street No. 90, Nangang District, Harbin 150000, China and [2]Zhengzhou Research Institute, Harbin Institute of Technology, Xidazhi Street No. 90, Nangang District, Harbin, Heilongjiang 150000, China and [3]College of Veterinary Medicine, Northeast Agricultural University, Harbin, Heilongjiang Province, Postcode 150038, China and [4]Department of Otorhinolaryngology, Harbin Institute of Technology Hospital, Harbin, Heilongjiang Province, Postcode 150038, China

[*]zty2009@hit.edu.cn; zhuyan8285@sina.com
[†]Contributed equally.

Abstract

Background: With the growing recognition of the important roles non-coding RNAs (ncRNAs) play in various biological functions, especially their potential involvement in many human diseases, predicting ncRNA–disease associations has become a key challenge in biomedical research.
Results: Although many computational methods have been proposed to predict ncRNA–disease associations, most of these methods focus on a single type of ncRNA. However, the competitive and cooperative interactions among different types of ncRNAs are closely related to their functional roles in disease associations. To address this limitation, we propose a novel computational framework, **PanGIA** (Pan-ncRNA Graph-Interaction Attention network), designed to simultaneously predict potential associations between multiple types of non-coding RNAs, including miRNA, lncRNA, circRNA, and piRNA, and diseases. Experimental results show that PanGIA outperforms type-specific SOTA methods in both individual and comprehensive predictions. It remains robust even when nodes or ncRNA types are removed, and ablation studies confirm the benefits of cross-type information. PanGIA also outperforms several single-type state-of-the-art methods across multiple metrics.
Conclusions: PanGIA demonstrates significant advantages in predicting disease associations for different types of ncRNAs, including miRNA, lncRNA, circRNA, and piRNA. Case studies further confirm the accuracy of the model's predictions, as all high-confidence associations were supported by literature evidence. This demonstrates the model's strong biological interpretability and promising potential for practical applications. The successful application of PanGIA provides a new paradigm for exploring disease-associated ncRNAs, highlighting their immense potential in the field of biomedical research.

Key words: Heterogeneous Graph Attention Network, Mixture-of-Experts, Cross-task Attention Mechanism, ncRNA–Disease Association

Introduction

Non-coding RNAs (ncRNAs) refer to a class of RNA molecules that do not encode proteins but play crucial roles in various biological processes, such as post-transcriptional regulation, epigenetic modification, and cellular signaling. In recent years, with the advancement of high-throughput sequencing technologies and functional genomics, an increasing number of ncRNAs have been identified

Compiled on: September 28, 2025.
Draft manuscript prepared by the author.

as closely associated with a wide range of complex human diseases [1, 2, 3, 4]. A growing body of experimental evidence has demonstrated that aberrant expression or dysfunction of ncRNAs is involved in the pathogenesis of major diseases, including cancer, neurodegenerative disorders, and cardiovascular diseases. Therefore, uncovering the potential associations between ncRNAs and diseases not only helps to elucidate the molecular mechanisms underlying complex diseases, but also provides theoretical support for early diagnosis, biomarker discovery, and personalized treatment strategies. In particular, ncRNA regulatory mechanisms have emerged as a research hotspot in fields such as oncology, neurological disorders, and cardiovascular disease.

Among the various types of ncRNAs, small RNAs such as microRNAs (miRNAs) have been extensively studied and are well-recognized for their post-transcriptional silencing functions through binding to target mRNAs. They have demonstrated significant potential as biomarkers in a wide range of diseases [5, 6].

Circular RNAs (circRNAs), owing to their covalently closed-loop structures that confer high stability, can function as competitive endogenous RNAs (ceRNAs) for microRNAs (miRNAs) or interact with RNA-binding proteins. Increasing evidence has demonstrated that circRNAs play critical regulatory roles and possess considerable potential for clinical applications across various disease contexts [7, 8].

Long ncRNAs (lncRNAs), which function by interacting with DNA, RNA, or proteins, are involved in processes such as chromatin modification and transcriptional regulation, and have been found to play crucial roles in tumorigenesis, cell proliferation, and immune modulation [9, 10].

PIWI-interacting RNAs (piRNAs), initially thought to function predominantly in germ cells by suppressing transposable elements to maintain genome stability, have more recently been shown to exert regulatory functions in somatic cells as well. These piRNAs are increasingly associated with various cancers and metabolic disorders [11, 12].

Despite their critical roles in gene regulation and disease mechanisms, experimental identification of non-coding RNA (ncRNA)–disease associations remains costly and time-consuming, limiting its scalability for large-scale studies. As a result, computational methods have gained increasing attention for their ability to efficiently and cost-effectively predict ncRNA–disease associations.

For miRNA, representative methods such as IMCMDA [13] leverage an integrated similarity network combined with a bilateral diffusion model to predict potential disease–miRNA associations. Regarding lncRNAs, LncDisAP [14] incorporates multiple similarity features and employs deep representation learning to effectively uncover latent associations. In the case of piRNAs, IPiDA-GBNN [15] enhances predictive accuracy by integrating graph neural networks with multi-feature representations. For circRNAs, existing approaches include GCNCDA [16], which constructs a heterogeneous graph structure and applies graph convolutional networks to learn circRNA–disease relationships. Additionally, CRBPSA [17] exploits sequence- and structure-aware attention mechanisms to identify circRNA–RBP interaction sites, thereby offering new insights into circRNA functionality. More recently, StackCirRNAPred [18] adopts a stacked ensemble learning strategy to achieve accurate classification of long circRNAs and other lncRNAs by integrating features from multiple sources.

Although these methods have achieved promising results within their respective ncRNA categories, they typically focus on a single type of ncRNA, ignoring the complex interplay and competition among different ncRNAs. For instance, miRNAs may interact with lncRNAs or circRNAs through the competing endogenous RNA (ceRNA) mechanism, jointly regulating disease-related pathways. These interactions form a complex regulatory network, yet existing approaches lack comprehensive modeling of both cross-ncRNA relationships and multi-type ncRNA–disease associations.

Therefore, there is a pressing need for novel computational frameworks that can jointly model the interrelations among various ncRNA types and their associations with diseases, enabling the discovery of previously unknown ncRNA–disease links through a more holistic understanding of their regulatory dynamics.

Despite the availability of several specialized repositories, such as miR2Disease, circR2Disease, LncRNADisease, and piRDisease, which systematically curate associations between non-coding RNAs (ncRNAs) and human diseases [19, 20, 21, 22], these databases are inherently constrained by their reliance on experimentally derived evidence. The acquisition of such evidence is both resource-intensive and time-consuming, with its breadth inherently limited by laboratory conditions and prevailing research focuses. Moreover, most current studies are restricted to individual ncRNA classes, thereby neglecting potential crosstalk and cooperative interactions among distinct ncRNA species in disease pathogenesis. Consequently, existing resources remain insufficient to fully capture the complexity of ncRNA-mediated regulatory networks.

In conventional studies, most computational approaches focus on a single type of non-coding RNA (ncRNA), such as miRNA, circRNA, lncRNA, or piRNA, and are typically tailored to the specific features and data types associated with that category. Examples include IMCMDA [13], LncDisAP [14], IPiDA-GBNN [15], and GCNCDA [16], among others. These models are generally built upon sequence information, structural properties, or expression profiles unique to the targeted ncRNA type. However, such type-specific methods exhibit clear limitations in their generalizability, as they are often not applicable to other classes of ncRNAs.

This limitation arises from the substantial differences in structure, biological function, and disease-related mechanisms among various ncRNA types. For instance, miRNAs primarily function through post-transcriptional repression by targeting mRNAs, whereas lncRNAs are involved in gene regulation and chromatin remodeling. CircRNAs are known to act as "sponges" for miRNAs, and piRNAs are mainly implicated in post-transcriptional regulation and transposon silencing. Consequently, models focusing exclusively on one ncRNA type tend to ignore the potential interactions and synergies among different ncRNA categories, thereby restricting their applicability and limiting their potential to uncover cross-type regulatory mechanisms in disease contexts.

Furthermore, single-type RNA-based approaches are inadequate in capturing the complex biological interactions that may exist across different RNA types. For example, miRNAs may indirectly influence disease development by regulating lncRNA expression; circRNAs may impact disease progression through interactions with miRNAs; and piRNAs may engage with other RNA species in various biological processes. Since traditional methods are confined to individual RNA types, they are unable to fully elucidate the potential cross-talk and co-regulatory mechanisms among diverse ncRNA classes.

Therefore, there is an urgent need to develop an efficient and scalable computational prediction model capable of systematically identifying potential associations between different types of ncRNAs and diseases. Such a model would not only compensate for the limitations of experimental data but also expand the knowledge graph of disease regulatory networks.

In this paper, we propose **PanGIA** (Pan-ncRNA Graph-Interaction Attention network), a novel framework for comprehensive ncRNA–disease association prediction. To address the challenge of feature heterogeneity, PanGIA constructs a heterogeneous graph that integrates multi-source data, including sequence information, functional similarity, and interaction networks of ncRNAs. To capture comprehensive and layered associations, it employs a cross-task attention mechanism combined with a Mixture-of-Experts architecture to dynamically learn the multi-level interactions between ncRNAs and diseases. Notably, PanGIA encompasses four representative classes of non-coding RNAs—miRNAs, lncRNAs, circRNAs, and piRNAs—which exhibit distinct characteristics in terms of length, structure, regulatory mechanisms, and func-

tional roles. These classes, owing to their complementary and representative nature in current research, are collectively referred to as pan-ncRNAs [23]. By adopting a pan-ncRNA perspective, PanGIA not only overcomes the limitations of single-type ncRNA studies but also provides a more comprehensive understanding of the multilayered regulatory roles of ncRNAs in disease.

The main contributions of our work are summarized as follows:

- **Pan-ncRNA integration:** PanGIA jointly models four types of ncRNAs (miRNA, lncRNA, circRNA, and piRNA), overcoming the limitations of single-type approaches.
- **Heterogeneous graph fusion:** It constructs a heterogeneous graph to integrate sequence, semantic, and functional data, capturing complex ncRNA–disease relationships.
- **Cross-task attention:** A Mixture-of-Experts module with cross-task attention enhances feature sharing across ncRNA types and improves prediction accuracy.
- **Superior performance:** PanGIA achieves higher AUC, AUPR, and rank metrics than baseline models, demonstrating strong generalization and reliability.

Materials

Our study involves multiple classes of non-coding RNAs and requires the simultaneous acquisition of their sequence information and disease association data. The databases utilized in this study are listed as follows:

- **miRNA**:The associations between miRNAs and diseases were obtained from the HMDD v4.0 database [24], while the sequence information of miRNAs was retrieved from the miRBase database [25].
- **LncRNA/circRNA:** This study includes lncRNA and circRNA associations with diseases, with data obtained from LncRNADisease v3.0 [26]. The sequence information of circRNAs was retrieved from the circBase database [27]. In contrast, lncRNA sequences were collected from two sources: GENCODE [28] and NONCODE [29].
- **piRNA**:The associations between piRNAs and diseases were obtained from the piRDisease v1.0 [21] database, and the sequence information was retrieved from the piRBase [30] and piRNAdb [31] databases.
- **Disease:** This study utilizes Disease Ontology Identifiers (DOIDs) to construct the disease similarity matrix, with corresponding information obtained from the Disease Ontology database [32].

The construction of the ncRNA–disease association network was based on merging data entries from the aforementioned association databases.

Methods

We propose a novel model named **PanGIA** (Pan-ncRNA Graph-Interaction Attention network), which is built upon the Heterogeneous Graph Attention Network (HAN) and a Mixture of Experts (MoE) framework. The model is designed to predict associations between pan-ncRNAs and diseases. The overall workflow of **PanGIA** is illustrated in Figure 1, and consists of three main steps:

i. **Pretraining:** ncRNA Node Embeddings via DNABERT6.
ii. **Data Processing:** Generation of Heterogeneous Networks.
iii. **Model Construction:** Multi-Task Association Prediction via HAN and Mixture-of-Experts with Cross-Task Attention

Pretraining: ncRNA Node Embeddings via DNABERT6

In this study, we leveraged the DNABERT6 [33] model to pretrain ncRNA sequences and obtain high-quality node embeddings. DNABERT represents a recent class of methods that adapt the BERT architecture, originally developed in natural language processing, to genomic sequence modeling. The central idea is to segment DNA sequences into fixed-length k-mers, thereby treating the genomic sequence as a special type of "language." By pre-training on large-scale genomic corpora, DNABERT is able to capture contextual dependencies within sequences and has demonstrated superior performance compared to traditional feature engineering approaches in a variety of downstream tasks, such as promoter prediction and transcription factor binding site identification. Building on this concept, we employed the DNABERT6 variant (with k=6) to model ncRNA sequences, thereby generating embeddings suitable for subsequent graph-based representation learning. The overall workflow is illustrated in Figure 1(A) and can be summarized in the following steps:

Tokenization of ncRNA Sequences

We first segmented the ncRNA sequences into fixed-length k-mers of size six. Prior to tokenization, all uracil (U) bases in the RNA sequences were systematically replaced with thymine (T) to ensure compatibility with DNA-based models such as DNABERT. The fundamental rationale of k-mer tokenization is to conceptualize DNA/RNA sequences as a specialized form of "language," in which each nucleotide fragment of length six serves as an independent lexical unit. Previous studies have demonstrated that setting k=6 provides superior performance across a wide range of genomic modeling tasks, as it effectively preserves local sequence features while simultaneously capturing long-range dependencies [33, 34, 35]. This strategy establishes a solid foundation for subsequent deep representation learning.

Masked Language Model Pretraining

After obtaining the 6-mer token sequences, we employed a Masked Language Model (MLM) pretraining strategy, wherein a subset of tokens was randomly masked and the model was required to recover the original tokens based on their surrounding context. Specifically, certain k-mer tokens in the input sequence were randomly replaced by a mask symbol, and the model was trained to reconstruct the original tokens conditioned on the unmasked context, thereby enabling the learning of contextual dependencies within the sequence. The corresponding objective function can be formally expressed as:

$$\mathcal{L}_{\mathrm{MLM}} = -\sum_{i \in \mathcal{M}} \log P(x_i \mid x_{\setminus \mathcal{M}}; \theta), \tag{1}$$

where $\mathcal{M}$ denotes the set of masked positions, x_i is the masked token, $x_{\setminus \mathcal{M}}$ represents the unmasked context tokens, and θ denotes the model parameters.

From a biological perspective, this mechanism facilitates the identification of potential functional motifs and enhances the ability to capture their regulatory roles under diverse contextual environments.

Embedding Representation

At the input layer, each 6-mer token is mapped into a dense vector representation composed of three components:

- **Token Embedding**: captures the semantic features of the current 6-mer.
- **Positional Embedding**: encodes the positional information of each token within the sequence, thereby enabling the model to preserve the linear order of nucleotides.
- **Segment Embedding**: used to differentiate between distinct segments in concatenated sequences.

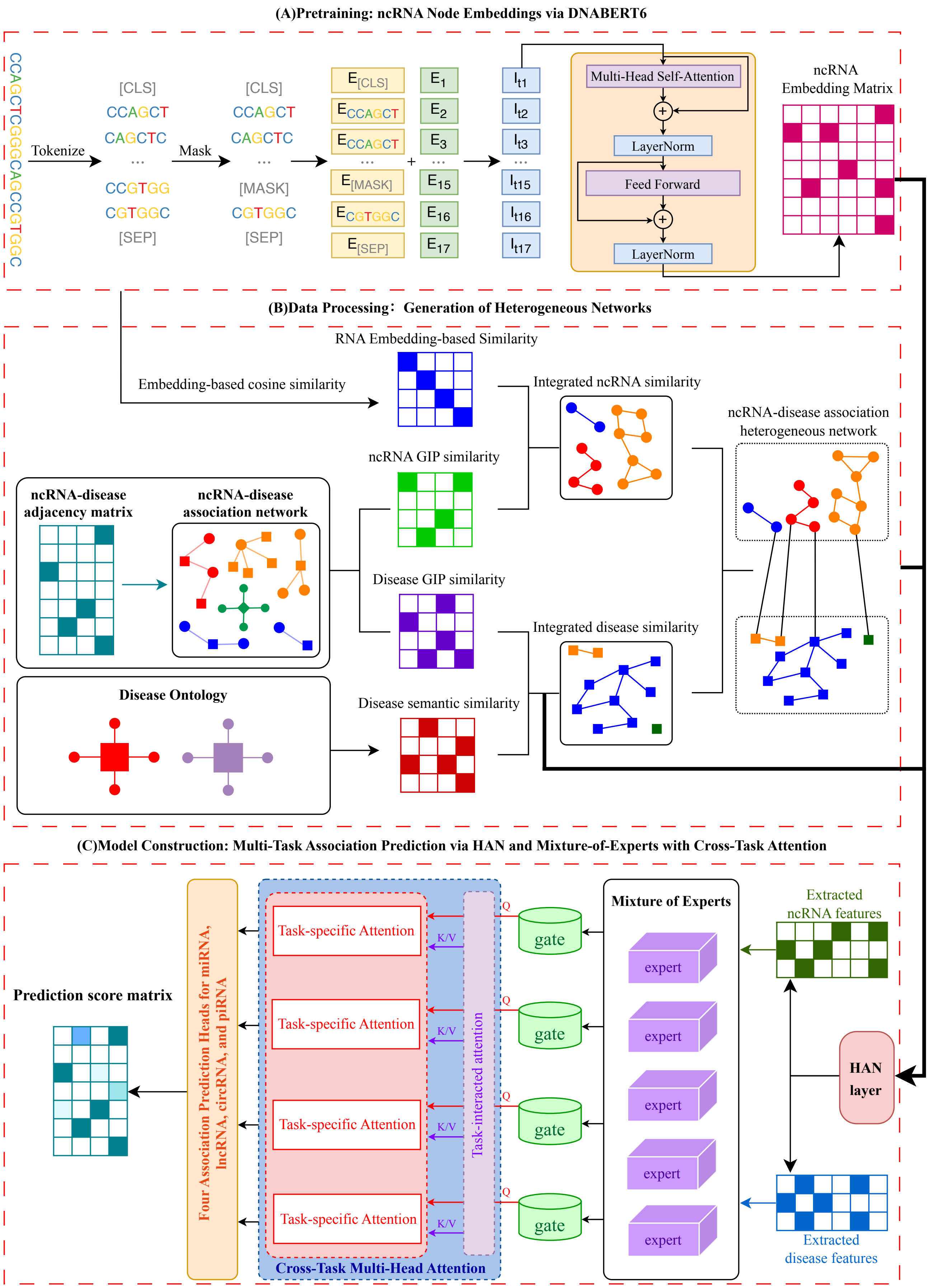

Figure 1. The structure of PanGIA.

Formally, the overall embedding of a token can be expressed as:

$$\mathbf{e}_i = \mathbf{e}_i^{\text{token}} + \mathbf{e}_i^{\text{pos}} + \mathbf{e}_i^{\text{seg}}, \tag{2}$$

where $\mathbf{e}_i^{\text{token}}$, $\mathbf{e}_i^{\text{pos}}$, and $\mathbf{e}_i^{\text{seg}}$ denote the token, positional, and segment embeddings of the i-th token, respectively.

This multi-level embedding strategy enables the model to simultaneously retain local nucleotide features while capturing the global topological structure of the sequence, thereby providing a comprehensive representation for downstream tasks.

Transformer Encoding and ncRNA Embedding Matrix

After obtaining the token-level embeddings, the sequence representations are fed into a stack of Transformer encoders. The core component of the Transformer is the multi-head self-attention mechanism, which enables the model to capture dependencies among sequence fragments in multiple subspaces. For ncRNA sequences, this mechanism is particularly important, as functional elements such as binding sites or seed regions may exhibit interactions spanning long distances along the sequence. By stacking multiple layers of self-attention and feed-forward networks, the model is able to generate increasingly rich contextual representations.

Ultimately, the model integrates the contextual information of each sequence into an embedding matrix that not only captures local nucleotide fragment features but also encodes long-range dependencies and global sequence semantics. Compared with traditional one-hot encoding or manually engineered sequence features, the embeddings generated by DNABERT are more comprehensive and robust. For RNA nodes, we employ DNABERT embeddings of 768 dimensions as the input features. This embedding matrix is further utilized as the node feature representation in graph-based learning, thereby providing a high-quality foundation for predicting ncRNA–disease associations.

Data Processing:Generation of Heterogeneous Networks

Heterogeneous Graph Construction

In this study, we first preprocessed the raw ncRNA sequence data and their known associations with diseases in order to construct a cross-modal heterogeneous network, which serves as the input foundation for the PanGIA model. Formally, the heterogeneous graph is defined as

$$\mathcal{G} = (\mathcal{V}, \mathcal{E}), \tag{3}$$

where the node set is given by

$$\mathcal{V} = \mathcal{V}_{\text{RNA}} \cup \mathcal{V}_{\text{dis}}, \tag{4}$$

with $\mathcal{V}_{\text{RNA}}$ denoting the set of RNA nodes and $\mathcal{V}_{\text{dis}}$ denoting the set of disease nodes.

The edge set $\mathcal{E}$ is composed of multiple types of relationships, including the following:

- **RNA–disease associations:**

$$\mathcal{E}_{\text{RNA-dis}} = \{(r_i, d_j) \mid A_{ij} = 1\}, \tag{5}$$

where $A \in \{0,1\}^{|\mathcal{V}_{\text{RNA}}| \times |\mathcal{V}_{\text{dis}}|}$ represents the known RNA–disease association matrix. If $A_{ij} = 1$, this indicates that RNA node r_i is associated with disease node d_j.

- **RNA–RNA similarity edges:**

$$\mathcal{E}_{\text{RNA-RNA}} = \{(r_i, r_j, w_{ij}^{\text{RNA}}) \mid S_{ij}^{\text{RNA}} > 0\}, \tag{6}$$

where $S^{\text{RNA}} \in R^{|\mathcal{V}_{\text{RNA}}| \times |\mathcal{V}_{\text{RNA}}|}$ denotes the RNA similarity matrix. A weighted edge is established between two RNA nodes if their similarity score is greater than zero, with the edge weight denoted as w_{ij}^{RNA}.

- **Disease–disease similarity edges:**

$$\mathcal{E}_{\text{dis-dis}} = \{(d_i, d_j, w_{ij}^{\text{dis}}) \mid S_{ij}^{\text{dis}} > 0\}, \tag{7}$$

where $S^{\text{dis}} \in R^{|\mathcal{V}_{\text{dis}}| \times |\mathcal{V}_{\text{dis}}|}$ denotes the disease similarity matrix. Similarly, if the similarity score between two diseases is greater than zero, a weighted edge is constructed with weight w_{ij}^{dis}.

Construction of ncRNA Similarity Matrices

To model intra-class similarities among four major categories of non-coding RNAs (ncRNAs)—miRNA, lncRNA, circRNA, and piRNA—we constructed similarity matrices based on embedding representations rather than conventional sequence alignment. Specifically, each ncRNA sequence was encoded into a dense embedding vector $\mathbf{h}_i \in R^d$ using a pretrained model. The similarity between any two sequences x_i and x_j of the same category was then quantified via cosine similarity:

$$\text{sim}(x_i, x_j) = \frac{\mathbf{h}_i \cdot \mathbf{h}_j}{\|\mathbf{h}_i\| \, \|\mathbf{h}_j\|}, \tag{8}$$

where $\mathbf{h}_i$ and $\mathbf{h}_j$ denote the embedding vectors of sequences x_i and x_j, respectively. After normalization, the similarity values were constrained within the interval $[0, 1]$, thereby ensuring consistency across different ncRNA types.

Finally, the similarity matrices for all ncRNAs were organized into a block-diagonal structure:

$$\mathbf{S}_{\text{RNA}}^{\text{seq}} = S_{\text{mi}}^{\text{seq}} 0000 S_{\text{circ}}^{\text{seq}} 0000 S_{\text{lnc}}^{\text{seq}} 0000 S_{\text{pi}}^{\text{seq}}, \tag{9}$$

where $S_{\text{mi}}^{\text{seq}}$, $S_{\text{circ}}^{\text{seq}}$, $S_{\text{lnc}}^{\text{seq}}$, and $S_{\text{pi}}^{\text{seq}}$ correspond to the cosine similarity matrices of miRNA, circRNA, lncRNA, and piRNA, respectively. This block-diagonal representation provides a structured foundation for integrating multi-class ncRNA similarities into downstream graph-based learning.

Based on the constructed ncRNA–disease association network, we next compute the functional similarity of non-coding RNAs using the Gaussian Interaction Profile (GIP) kernel function. The corresponding formula is given as follows:

$$\mathbf{S}_{\text{RNA}}^{\text{GIP}}(r_i, r_j) = \exp\left(-\lambda_{\text{RNA}} \left\|\mathbf{A}(r_i, :) - \mathbf{A}(r_j, :)\right\|^2\right) \tag{10}$$

In this formulation, $\mathbf{A}(r_i, :)$ and $\mathbf{A}(r_j, :)$ represent the vectors corresponding to the i-th and j-th rows of the adjacency matrix $\mathbf{A}$, respectively. The parameter λ_{RNA} denotes the bandwidth coefficient of the kernel function, which is defined as follows:

$$\lambda_{\text{RNA}} = \frac{1}{\frac{1}{N_r} \sum_{k=1}^{N_r} \left\|\mathbf{A}(r_k, :)\right\|^2} \tag{11}$$

N_r denotes the total number of ncRNAs, and $\mathbf{A}(r_k, :)$ represents the vector corresponding to the k-th row of the adjacency matrix $\mathbf{A}$. Subsequently, we integrate the sequence similarity and GIP-based functional similarity to obtain the final ncRNA similarity matrix:

$$\mathbf{S}^{\text{RNA}} = \frac{\mathbf{S}_{\text{RNA}}^{\text{seq}} + \mathbf{S}_{\text{RNA}}^{\text{GIP}}}{2} \tag{12}$$

Construction of the Disease Similarity Matrix

On the disease side, we constructed two types of disease similarity networks based on different approaches: (i) a semantic similar-

ity matrix calculated using Disease Ontology, and (ii) a GIP-based similarity matrix generated from disease interaction profiles. By integrating these two sources of similarity information, we obtained a comprehensive disease similarity network to enhance the accuracy of disease representation [36, 37, 38].

Disease Ontology is a structured ontology that organizes various diseases and their hierarchical relationships. Each disease node in the ontology is assigned a unique identifier and may be associated with descriptive attributes, such as symptoms and causes. The hierarchical structure of the ontology typically resembles a tree, where parent nodes represent broader disease categories and child nodes correspond to more specific diseases. In this study, we employ the Jaccard similarity coefficient to compute the semantic similarity matrix between diseases, defined as follows:

$$\mathbf{S}_{\mathrm{dis}}^{\mathrm{sem}} = \frac{|\mathrm{Ancestors}(d_1) \cap \mathrm{Ancestors}(d_2)|}{|\mathrm{Ancestors}(d_1) \cup \mathrm{Ancestors}(d_2)|} \tag{13}$$

In this formulation, $\mathrm{Ancestors}(d)$ denotes the set of ancestor nodes of the disease node d, and $|A|$ represents the cardinality (i.e., the number of elements) of the set A.

The functional similarity of diseases based on the Gaussian Interaction Profile (GIP) kernel is calculated as follows:

$$\mathbf{S}_{\mathrm{dis}}^{\mathrm{GIP}}(d_i, d_j) = \exp\left(-\lambda_{\mathrm{dis}} \left\|\mathbf{A}(:, d_i) - \mathbf{A}(:, d_j)\right\|^2\right) \tag{14}$$

In this formulation, $\mathbf{A}(:, d_i)$ and $\mathbf{A}(:, d_j)$ represent the vectors corresponding to the i-th and j-th columns of the adjacency matrix $\mathbf{A}$, respectively. The parameter λ_{dis} denotes the bandwidth coefficient of the kernel function, which is defined as follows:

$$\lambda_{\mathrm{dis}} = \frac{1}{\frac{1}{N_{\mathrm{d}}} \sum_{k=1}^{N_{\mathrm{d}}} \left\|\mathbf{A}(:, d_k)\right\|^2} \tag{15}$$

N_d denotes the total number of diseases. Subsequently, we integrate the semantic similarity and GIP-based functional similarity to obtain the final integrated disease similarity matrix:

$$\mathbf{S}^{\mathrm{dis}} = \frac{\mathbf{S}_{\mathrm{dis}}^{\mathrm{sem}} + \mathbf{S}_{\mathrm{dis}}^{\mathrm{GIP}}}{2} \tag{16}$$

We integrate the constructed ncRNA similarity network, the disease similarity network, and the known ncRNA–disease associations to form a unified ncRNA–disease heterogeneous graph, denoted as $\mathcal{G}$.

Model Construction: Multi-Task Association Prediction via HAN and Mixture-of-Experts with Cross-Task Attention

We propose a novel multi-task relational prediction framework that integrates a Heterogeneous Graph Attention Network (HAN) with a Mixture-of-Experts (MoE) mechanism. Through a Cross-Task Attention mechanism, the framework enables collaborative modeling across tasks, enhancing both task generalization and interaction expression capabilities. The overall structure of the model is depicted in Figure 1(C), which consists of the following five main components:

Feature Representation and Heterogeneous Graph Modeling

The model input consists of the ncRNA embedding matrix $\mathbf{X}^{\mathrm{rna}} \in R^{N_r \times d_r}$ and the disease embedding matrix $\mathbf{X}^{\mathrm{dis}} \in R^{N_d \times d_d}$, where N_r and N_d represent the number of ncRNAs and diseases, respectively, and d_r and d_d correspond to their embedding dimensions. Since the original feature dimensions of ncRNAs and diseases may differ, we first map the disease embeddings into the same space as the ncRNA embeddings:

$$\mathbf{X}_{\mathrm{proj}}^{\mathrm{dis}} = \mathbf{X}^{\mathrm{dis}} \mathbf{W}_d \in R^{N_d \times d_r} \tag{17}$$

Where $\mathbf{W}_d \in R^{d_d \times d_r}$ is a learnable linear transformation matrix.

Next, the heterogeneous network $\mathcal{N}_H$, based on ncRNA-disease associations, along with the ncRNA embedding matrix $\mathbf{X}^{\mathrm{rna}}$ and disease embedding matrix $\mathbf{X}^{\mathrm{dis}}$, is fed into the HAN model. A multi-head attention mechanism, guided by meta-paths, is employed to extract higher-order semantic features from the graph structure. The output of the HAN encoder is:

$$\mathbf{H}^{\mathrm{rna}}, \mathbf{H}^{\mathrm{dis}} = \mathrm{HANEncoder}(\mathbf{X}^{\mathrm{rna}}, \mathbf{X}_{\mathrm{proj}}^{\mathrm{dis}}, \mathcal{N}_H) \tag{18}$$

Where $\mathbf{H}^{\mathrm{rna}}, \mathbf{H}^{\mathrm{dis}} \in R^{N \times d_h}$ represent the hidden representations of ncRNAs and diseases extracted by the HAN layer, and d_h denotes the intermediate hidden dimension.

Expert Pool and Global Disease Information Fusion

To integrate global disease semantics, we average the representations of all disease nodes to obtain the global disease feature:

$$\bar{\mathbf{H}}^{\mathrm{dis}} = \frac{1}{N_d} \sum_{i=1}^{N_d} \mathbf{H}_i^{\mathrm{dis}} \in R^{d_h} \tag{19}$$

We concatenate this with each ncRNA representation to form the fused representation:

$$\mathbf{F} = \left[\mathbf{H}^{\mathrm{rna}} \,\|\, \bar{\mathbf{H}}^{\mathrm{dis}}\right] \in R^{N_r \times 2d_h} \tag{20}$$

Subsequently, the fused representation is input into an expert pool consisting of K experts, where each expert is a nonlinear transformation module:

$$\mathcal{E}_k(\mathbf{F}) = \mathrm{ReLU}(\mathbf{F}\mathbf{W}_k + \mathbf{b}_k), \quad k = 1, \ldots, K \tag{21}$$

The outputs of all experts are then stacked:

$$\mathbf{E} = \mathrm{stack}(\mathcal{E}_1(\mathbf{F}), \ldots, \mathcal{E}_K(\mathbf{F})) \in R^{N_r \times K \times d_e} \tag{22}$$

Where d_e denotes the output dimension of each expert.

Multi-Task Gating Mechanism

For each specific task $t \in \{1, \ldots, T\}$, the corresponding ncRNA subset is $\mathcal{I}_t \subset \{1, \ldots, N_r\}$. We learn a gating network for each task to perform attention-based selection of experts in the expert pool:

- First, the task-related input representation $\mathbf{H}_t = \mathbf{H}^{\mathrm{rna}}[\mathcal{I}_t]$ is mapped to a query vector $\mathbf{Q}_t$;
- The expert representations are mapped to keys $\mathbf{K}_t$ and values $\mathbf{V}_t$;
- A multi-head attention mechanism is then used to compute the attention-weighted expert representation:

$$\mathbf{A}_t = \mathrm{softmax}(\mathrm{MultiHeadAttn}(\mathbf{Q}_t, \mathbf{K}_t, \mathbf{V}_t)) \in R^{n_t \times K} \tag{23}$$

The final aggregation of the expert outputs results in the task feature representation:

$$\mathbf{u}_t = \sum_{k=1}^{K} \mathbf{A}_{t,:,k} \cdot \mathbf{E}_{\mathcal{I}_t,k} \in R^{n_t \times d_e} \tag{24}$$

Cross-Task Attention Interaction

To model the potential correlations between tasks, we input the aggregated representations of all tasks (after average pooling) into

a cross-task multi-head attention module:

$$\mathbf{U} = [\bar{\mathbf{u}}_1, \ldots, \bar{\mathbf{u}}_T] \in R^{T \times d_e}, \quad \bar{\mathbf{u}}_t = \frac{1}{n_t} \sum_{i=1}^{n_t} \mathbf{u}_{t,i} \tag{25}$$

$$\mathbf{U}' = \text{MultiHeadAttn}(\mathbf{U}, \mathbf{U}, \mathbf{U}) \in R^{T \times d_e} \tag{26}$$

The cross-task global representation $\mathbf{U}'_t$ is then concatenated with the original task representation $\mathbf{u}_t$:

$$\mathbf{z}_t = \text{ReLU}([\mathbf{u}_t \parallel \mathbf{U}'_t]) \in R^{n_t \times d_e} \tag{27}$$

Relational Prediction and Output Layer

The representations of all diseases are projected to the expert dimension through a linear transformation:

$$\tilde{\mathbf{H}}^{\text{dis}} = \mathbf{H}^{\text{dis}} \mathbf{W}_{\text{proj}} \in R^{N_d \times d_e} \tag{28}$$

Finally, the association score between each ncRNA and all diseases for each task is calculated through the dot product, followed by normalization using the sigmoid function:

$$\hat{\mathbf{Y}}_t = \sigma(\mathbf{z}_t \cdot \tilde{\mathbf{H}}^{\text{dis}^\top}) \in R^{n_t \times N_d} \tag{29}$$

In this framework, the association prediction task is formulated as a binary classification problem. For each task t, the task-specific MLP transforms the RNA representations into $\mathbf{z}_t$, which captures task-refined structural and semantic features. Each disease node representation is projected into the same latent space, yielding $\tilde{\mathbf{H}}^{\text{dis}}$. The association score between an RNA node i and a disease node j is computed as the dot product:

$$s_{t,ij} = \mathbf{z}_{t,i} \cdot \tilde{\mathbf{h}}_j^{\text{dis}}. \tag{30}$$

This score is then passed through a sigmoid function to produce the probability:

$$\hat{y}_{t,ij} = \sigma(s_{t,ij}) \in [0, 1], \tag{31}$$

which indicates the likelihood that RNA i is associated with disease j. Hence, the MLP does not serve as the final classifier but rather as a task-dependent feature extractor, while the prediction itself is achieved through the interaction between RNA and disease embeddings.

Results

benchmark on various ncRNAs

In this study, we evaluated the performance of different models using five-fold cross-validation and employed Rank Index, AUC (Area Under the Curve), and AUPR (Area Under the Precision-Recall Curve) as evaluation metrics.

Under the evaluation of five-fold cross-validation, the performance of various models on the pan-ncRNA–disease association prediction task is summarized in Table 1. Compared with the baseline methods, PanGIA consistently achieved the best performance across multiple evaluation metrics.

To comprehensively evaluate the performance of the PanGIA model in multi-type ncRNA-disease association prediction, this study conducted comparative experiments using various existing mainstream methods across four types of ncRNAs—miRNA, lncRNA, piRNA, and circRNA. The models' performance was assessed using AUC, AUPR, and Rank Index metrics. As shown in the table, PanGIA outperformed all other methods across all RNA types. Furthermore, when all ncRNA types were integrated, the model's performance was further enhanced, achieving the highest AUC, AUPR, and the lowest Rank Index. These results strongly demonstrate PanGIA's exceptional generalization ability and predictive accuracy in multi-type ncRNA-disease association prediction.

Multi-task synchronous prediction can provide key information

To validate the advantages of our proposed framework in leveraging the full-spectrum heterogeneous association network and the neural network architecture design, we not only examined the performance after ablating critical network modules but also progressively reduced the scale of full-spectrum data to evaluate the unique contribution of pan-ncRNA–disease association information.

Stepwise Reduction of Heterogeneity in the Network

To systematically evaluate the impact of reduced training data on model performance, we designed two downsampling strategies to progressively decrease the amount of information available to the PanGIA model: (1) a random uniform subsampling strategy, and (2) an RNA-type-based node selection strategy.

In the random uniform subsampling strategy, we adopted a straightforward uniform sampling method. Specifically, a certain proportion of ncRNA and disease nodes were randomly and uniformly removed from the heterogeneous graph, along with their associated edges. This ensured that both types of nodes (ncRNAs and diseases) were reduced at the same rate, preserving the relative balance between modalities in the network while decreasing the overall graph size. By gradually scaling down the input network, we simulated scenarios with limited data availability to examine how PanGIA performs under constrained information settings.

Based on this strategy, we conducted a systematic performance evaluation of the PanGIA model using 100%, 80%, 67%, and 50% of the original dataset for training. As shown in Figure 2, the corresponding evaluation metrics—AUC, AUPR, and Rank Index—consistently declined with reduced data scale. These results indicate that PanGIA is sensitive to the quantity of training data and that its predictive capability is notably affected under data-sparse conditions.

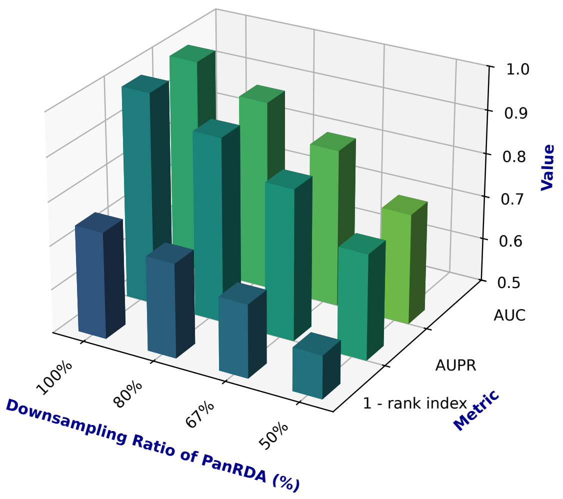

Figure 2. Performance comparison of PanGIA under different subsampling ratios.

In summary, this experiment underscores the critical importance of data completeness in achieving optimal predictive performance with PanGIA, highlighting the key role of full-spectrum

Table 1. Performance comparison of PanGIA and baseline models on ncRNA–disease association prediction tasks.

Model	RNA Category	AUC	Rank Index	AUPR
NIMGSA [39]	miRNA	0.947 ± 0.003	0.318 ± 0.006	0.682 ± 0.002
MINIMDA [40]	miRNA	0.918 ± 0.001	0.324 ± 0.003	0.904 ± 0.004
PanGIA	miRNA	0.926 ± 0.003	0.304 ± 0.003	0.914 ± 0.002
gGATLDA [41]	lncRNA	0.931 ± 0.001	0.283 ± 0.002	0.923 ± 0.005
LDGRNMF [42]	lncRNA	0.892 ± 0.005	0.328 ± 0.003	0.849 ± 0.004
PanGIA	lncRNA	0.933 ± 0.006	0.298 ± 0.001	0.927 ± 0.003
iPiDi-PUL [43]	piRNA	0.569 ± 0.026	0.444 ± 0.021	0.117 ± 0.008
PUTransGCN [44]	piRNA	0.930 ± 0.007	0.103 ± 0.006	0.598 ± 0.032
PanGIA	piRNA	0.934 ± 0.001	0.291 ± 0.007	0.929 ± 0.003
IGNSCDA [45]	circRNA	0.812 ± 0.003	0.331 ± 0.004	0.694 ± 0.006
GATCL2CD [46]	circRNA	0.931 ± 0.004	0.282 ± 0.007	0.879 ± 0.008
PanGIA	circRNA	0.927 ± 0.005	0.306 ± 0.003	0.914 ± 0.009
PanGIA	miRNA, lncRNA, piRNA, circRNA	0.988 ± 0.002	0.256 ± 0.001	0.985 ± 0.004

biological data in robust association prediction tasks.

To further evaluate the overall contribution of different ncRNA types to the predictive performance of the model, we conducted a stepwise ablation study by progressively removing specific categories of ncRNAs from the full pan-ncRNA set. As shown in Figure 3, we assessed model performance under various ncRNA combinations using AUC, AUPR, and Rank Index as evaluation metrics. The results demonstrate that the inclusion of all four ncRNA types (miRNA, lncRNA, circRNA, and piRNA) yields the best overall performance. In contrast, removing any single or multiple ncRNA types leads to a noticeable decline in one or more metrics, highlighting the complementary contributions of each ncRNA class to the overall prediction capability of the PanGIA framework.

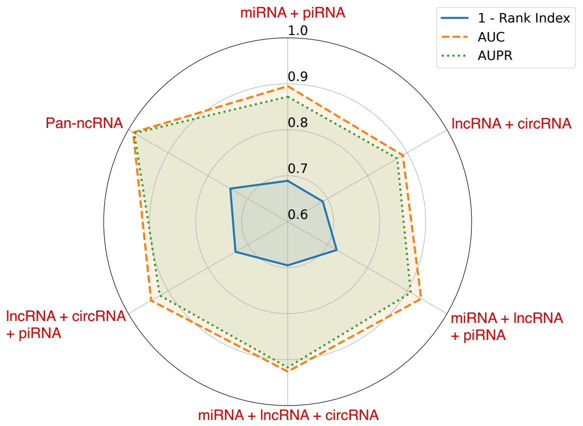

Figure 3. Performance of PanGIA with different ncRNA combinations.

Robust of PanGIA

To validate the effectiveness of key components in the PanGIA framework, we performed a series of ablation experiments by systematically removing core modules, including the heterogeneous graph attention network (HAN), the Mixture-of-Experts (MoE), and the cross-task attention mechanism. Additionally, a single-task learning variant was tested to contrast against the full multi-task framework. As illustrated in Figure 4, Figure 5 and Figure 6, performance metrics including AUC, AUPR, and Rank Index were measured for each ablation variant.

The results demonstrate that removing any of the core compo-

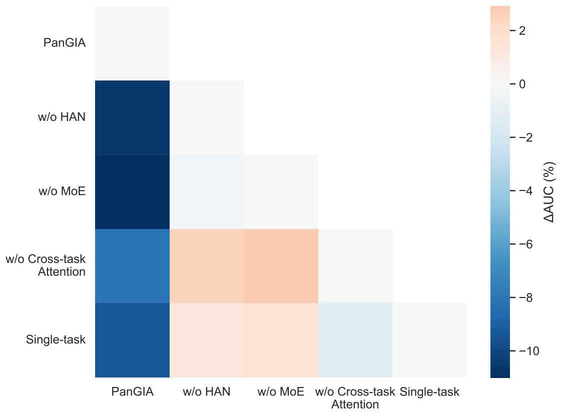

Figure 4. Performance comparison of PanGIA ablation variants on AUC.

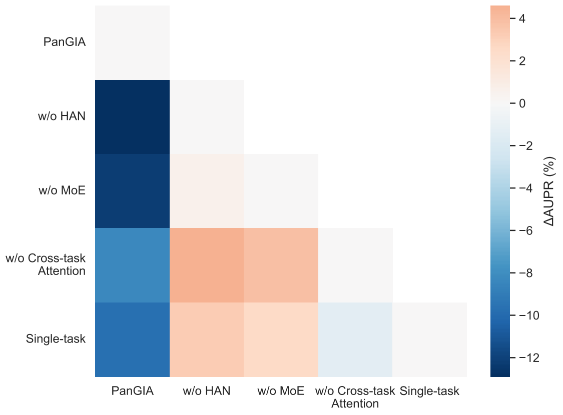

Figure 5. Performance comparison of PanGIA ablation variants on AUPR.

Table 2. Experimentally validated ncRNA–disease associations used in the case study

RNA Type	RNA Symbol	Disease Name	PMID
miRNA	miR-944	Glioblastoma	34233294
miRNA	miR-936	Glioblastoma	29218238
miRNA	miR-378	Osteoarthritis	35474736
miRNA	miR-139	Osteoarthritis	32185303
circRNA	CSPP1	Glioblastoma	32495924
circRNA	SCN3B	Glioblastoma	39289188
circRNA	ROCK1	Coronary Artery Disease	34236817
circRNA	WNK1	Coronary Artery Disease	31821324
lncRNA	LINC00324	Stomach carcinoma	32855634
lncRNA	LINC00691	Stomach carcinoma	32330554
lncRNA	RPSAP52	Stomach carcinoma	35322746
lncRNA	AFAP1-AS1	Cholangiocarcinoma	28938565
piRNA	DQ570326	Parkinson's disease	29986767
piRNA	DQ592957	Parkinson's disease	29986767
piRNA	DQ596377	Alzheimer's disease	28127595
piRNA	DQ597397	Renal Cell Carcinoma	25998508

Source: All associations were experimentally validated and supported by the referenced PubMed IDs (PMIDs).

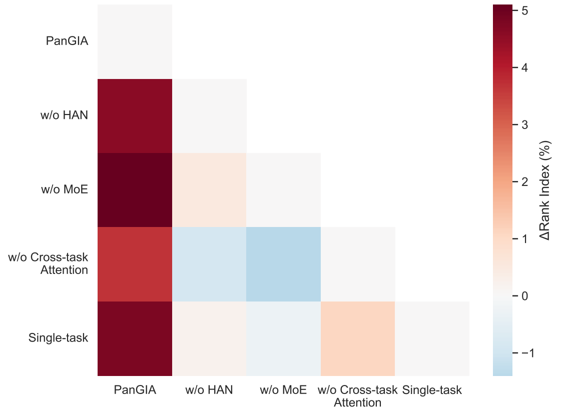

Figure 6. Performance comparison of PanGIA ablation variants on Rank Index.

nents leads to a noticeable performance decline across all metrics. Specifically, the removal of HAN or MoE resulted in significant drops in both AUC and AUPR, indicating the importance of structural and expert-based representation learning. Furthermore, disabling the cross-task attention mechanism impaired the model's ability to integrate information across tasks, reducing prediction accuracy. The single-task baseline also underperformed compared to the full model, highlighting the advantage of PanGIA's multi-task learning design. Overall, these findings confirm that each module contributes uniquely and substantially to the overall predictive power of PanGIA.

PanGIA reveals novel ncRNA-disease associations

In the case study, we selected high-confidence associations between various types of ncRNAs (miRNA, circRNA, lncRNA, and piRNA) and representative diseases as predicted by the PanGIA model. These predicted associations were organized and presented in Table 2, respectively. Through literature review, we confirmed that all the associations listed in the tables have been experimentally validated, with supporting evidence provided by the corresponding references (PMIDs).

In this case study, we focused on high-confidence miRNA–disease associations predicted by our model. Literature searches confirmed that these miRNAs are supported by clear biological mechanisms. For example, miR-944 derived from glioma stem cell exosomes directly downregulates VEGFC expression, further suppressing AKT/ERK signaling activity, thereby significantly reducing glioblastoma growth and angiogenesis [47]. Similarly, miR-936 is markedly downregulated in glioblastoma tissues, with its expression negatively correlated with tumor grade. Re-expression of miR-936 can target the CKS1 gene and inhibit the downstream AKT/ERK pathway, effectively blocking the cell cycle and suppressing tumor growth [48]. In osteoarthritis (OA), overexpression of miR-378 aggravates cartilage degeneration by suppressing autophagy in chondrocytes and inhibiting chondrogenic differentiation of bone marrow mesenchymal stem cells (BMSCs). Its targets, Atg2a and Sox6, are well characterized; conversely, the application of anti-miR-378 alleviates OA progression and promotes joint regeneration, highlighting its therapeutic potential [49]. In addition, miR-139 is significantly upregulated in OA-damaged cartilage and can be activated by IL-1β. By directly targeting MCPIP1, it relieves translational repression of IL-6, leading to elevated IL-6 and degradative enzymes such as MMP-13 and ADAMTS4, thereby promoting chondrocyte apoptosis and matrix degradation [50].

Regarding circRNAs, multiple experimental findings also support the model predictions. CSPP1 is markedly upregulated in glioblastoma tissues, closely associated with abnormal mitosis and proliferation of tumor cells [51]. Similarly, SCN3B has been identified as a key molecule in glioblastoma, with aberrant expression linked to enhanced tumor cell migration and invasion [52]. In cardiovascular disease, ROCK1-related circRNA plays an important role in coronary artery disease by regulating vascular smooth muscle cell contraction and apoptosis, thereby promoting disease progression [53]. Meanwhile, WNK1-derived circRNA is significantly upregulated in coronary artery disease patients, affecting endothelial function and ion channel homeostasis, thus accelerating atherosclerosis development [54]. These results further demonstrate the molecular significance of circRNAs in diverse diseases, validating the reliability and biological value of our model predictions.

For lncRNAs, several experimentally validated findings support the predicted associations. LINC00324 is significantly downregulated in stomach carcinoma, where it interacts with miR-3200-5p to regulate downstream BCAT1 expression, thereby inhibiting tumorigenesis [55]. Similarly, LINC00691 is upregulated in gastric cancer tissues and promotes proliferation and invasion by modulat-

ing the miR-9-5p/FGFR1 axis, suggesting its oncogenic role [56]. Furthermore, RPSAP52 enhances proliferation and inhibits apoptosis in gastric cancer by regulating the miR-665/STAT3 pathway, thus promoting tumor progression [57]. In cholangiocarcinoma, AFAP1-AS1 is markedly upregulated and promotes migration and invasion through transcriptional regulation of EMT-related genes, underscoring its pivotal role in tumor progression [58].

For piRNAs, most predicted associations have been experimentally verified to show differential expression and participation in pathological processes. For instance, DQ597397 is significantly upregulated in renal cell carcinoma cells compared with normal renal cells, suggesting its role in promoting tumor progression [59]. Conversely, DQ570326 and DQ592957 are downregulated in neurons derived from Parkinson's disease patients, potentially contributing to neurodegenerative mechanisms [60]. Moreover, DQ596377 is markedly upregulated in neurons from Alzheimer's disease patients, with expression levels 11.38-fold higher than those in normal brain cells, indicating its involvement in AD-specific neuropathological processes [61]. Collectively, these findings demonstrate that piRNAs play crucial molecular roles in the pathogenesis of multiple major diseases and further substantiate the reliability of our model predictions.

This result indicates that PanGIA performs excellently in the aforementioned case studies, demonstrating its capability to identify high-confidence associations between miRNAs, circRNAs, lncRNAs, and piRNAs with diseases. The unconfirmed associations predicted by PanGIA may serve as candidate targets for subsequent biological experiments and lay a solid foundation for the potential application of related ncRNAs in disease diagnosis and therapy.

Conclusion

In this study, we proposed **PanGIA**, a novel model for ncRNA–disease association prediction that integrates a Hierarchical Attention Network (HAN) with a Mixture-of-Experts architecture. Comprehensive experiments demonstrate that PanGIA achieves consistently superior performance across various RNA types, including miRNA, lncRNA, circRNA, and piRNA, validating the effectiveness of our multi-source feature extraction and multi-task modeling strategy.

PanGIA not only outperforms state-of-the-art methods on multiple evaluation metrics but also maintains robust and stable performance across different ncRNA categories. This indicates strong generalization and robustness of the model in handling heterogeneous RNA data and structures.

Through further case study analyses, we validated that several high-confidence predictions have been experimentally confirmed in the literature, highlighting the significant advantages of this method in terms of biological interpretability and result reliability. In particular, the ncRNA–disease associations predicted by PanGIA show substantial research and application value in fields such as neurological disorders, metabolic diseases, and cancer.

Overall, PanGIA demonstrates strong potential as a unified framework for pan-ncRNA–disease association prediction. It excels in both macro-level performance benchmarks and micro-level case reliability, suggesting excellent cross-task generalizability and interpretability. In future work, incorporating additional omics data and optimizing the network architecture may further enhance its predictive power, contributing to ncRNA functional studies, disease mechanism exploration, and the advancement of precision medicine and biomarker discovery.

Availability of Source Code and Requirements

- Project name: PanGIA
- Project homepage: `https://github.com/qiankunzizairen/PanGIA`
- License: MIT license
- Operating system: Linux
- Programming language: Python
- Package management: pip
- Hardware requirements: Standard workstation with at least 20GB memory; GPU recommended for training with more than 20GB memory.
- SciCrunch RRID: SCR_027069
- bio.tools ID: rna-disease-predictor
- DOME-DL: `https://registry.dome-ml.org/review/yi6cmalcqe`

Competing interests

No competing interest is declared.

Funding

Heilongjiang Province Basic Research Support Program for Outstanding Young Teachers(YQJH2023195)

Data Availability

The supporting data underlying this study are available in the GigaScience Database (GigaDB) [62].

References

1. Esteller M. Non-coding RNAs in human disease. Nature Reviews Genetics 2011 Dec;12(12):861–874. doi: 10.1038/nrg3074.
2. Loganathan T, Doss C GP. Non-coding RNAs in human health and disease: potential function as biomarkers and therapeutic targets. Functional & Integrative Genomics 2023 Mar;23(1):33. doi: 10.1007/s10142-022-00947-4.
3. Harries L. Long non-coding RNAs and human disease. Biochemical Society Transactions 2012 Aug;40(4):902–906. doi: 10.1042/BST20120020.
4. Li C, Ni YQ, Xu H, Xiang QY, Zhao Y, Zhan JK, et al. Roles and mechanisms of exosomal non-coding RNAs in human health and diseases. Signal Transduction and Targeted Therapy 2021 Nov;6(1):383. doi: 10.1038/s41392-021-00779-x.
5. Shi H, Xu J, Zhang G, Xu L, Li C, Wang L, et al. Walking the interactome to identify human miRNA-disease associations through the functional link between miRNA targets and disease genes. BMC Systems Biology 2013 Dec;7(1):101. doi: 10.1186/1752-0509-7-101.
6. Lu M, Zhang Q, Deng M, Miao J, Guo Y, Gao W, et al. An Analysis of Human MicroRNA and Disease Associations. PLoS ONE 2008 Oct;3(10):e3420. doi: 10.1371/journal.pone.0003420.
7. Zang J, Lu D, Xu A. The interaction of circRNAs and RNA binding proteins: An important part of circRNA maintenance and function. Journal of Neuroscience Research 2020 Jan;98(1):87–97. doi: 10.1002/jnr.24356.
8. Okholm TLH, Sathe S, Park SS, Kamstrup AB, Rasmussen AM, Shankar A, et al. Transcriptome-wide profiles of circular RNA and RNA-binding protein interactions reveal effects on circular RNA biogenesis and cancer pathway expression. Genome Medicine 2020 Dec;12(1):112. doi: 10.1186/s13073-020-00812-8.
9. Yan J, Wang R, Tan J. Recent advances in predicting lncRNA–disease associations based on computational methods. Drug Discovery Today 2023 Feb;28(2):103432. doi: 10.1016/j.drudis.2022.103432.
10. Yang X, Gao L, Guo X, Shi X, Wu H, Song F, et al. A Network

Based Method for Analysis of lncRNA-Disease Associations and Prediction of lncRNAs Implicated in Diseases. PLOS ONE 2014 Jan;9(1):e87797. doi: 10.1371/journal.pone.0087797.
11. Ali SD, Tayara H, Chong KT. Identification of piRNA disease associations using deep learning. Computational and Structural Biotechnology Journal 2022 Jan;20:1208–1217. doi: 10.1016/j.csbj.2022.02.026.
12. Rayford KJ, Cooley A, Rumph JT, Arun A, Rachakonda G, Villalta F, et al. piRNAs as Modulators of Disease Pathogenesis. International Journal of Molecular Sciences 2021 Feb;22(5):2373. doi: 10.3390/ijms22052373.
13. Li Z, Zhang Y, Bai Y, Xie X, Zeng L, Li Z, et al. IMC-MDA: Prediction of miRNA-disease association based on induction matrix completion. Mathematical Biosciences and Engineering 2023;20(6):10659–10674. doi: 10.3934/mbe.2023471.
14. Wang Y, Juan L, Peng J, Zang T, Wang Y. LncDisAP: A computation model for LncRNA-disease association prediction based on multiple biological datasets. BMC Bioinformatics 2019 Dec;20:582. doi: 10.1186/s12859-019-3081-1.
15. Qian Y, He Q, Deng L. iPiDA-GBNN:Identification of Piwi-interacting RNA-disease associations based on gradient boosting neural network. In: 2021 IEEE International Conference on Bioinformatics and Biomedicine (BIBM); 2021. p. 1045–1050. doi: 10.1109/BIBM52615.2021.9669592.
16. Wang L, You ZH, Li YM, Zheng K, Huang YA. GC-NCDA: A new method for predicting circRNA-disease associations based on Graph Convolutional Network Algorithm. PLoS computational biology 2020 May;16(5):e1007568. doi: 10.1371/journal.pcbi.1007568.
17. Cao C, Wang C, Dai Q, Zou Q, Wang T. CRBPSA: CircRNA-RBP interaction sites identification using sequence structural attention model. BMC biology 2024 Nov;22(1):260. doi: 10.1186/s12915-024-02055-0.
18. Wang X, Liu Y, Li J, Wang G. StackCirRNAPred: computational classification of long circRNA from other lncRNA based on stacking strategy. BMC bioinformatics 2022 Dec;23(1):563. doi: 10.1186/s12859-022-05118-7.
19. Bao Z, Yang Z, Huang Z, Zhou Y, Cui Q, Dong D. LncRNADisease 2.0: an updated database of long non-coding RNA-associated diseases. Nucleic Acids Research 2019 Jan;47(D1):D1034–D1037. doi: 10.1093/nar/gky905.
20. Fan C, Lei X, Tie J, Zhang Y, Wu FX, Pan Y. CircR2Disease v2.0: An Updated Web Server for Experimentally Validated circRNA–disease Associations and Its Application. Genomics, Proteomics & Bioinformatics 2022 Jun;20(3):435–445. doi: 10.1016/j.gpb.2021.10.002.
21. Muhammad A, Waheed R, Khan NA, Jiang H, Song X. piRDisease v1.0: a manually curated database for piRNA associated diseases. Database: The Journal of Biological Databases and Curation 2019 Jan;2019:baz052. doi: 10.1093/database/baz052.
22. Jiang Q, Wang Y, Hao Y, Juan L, Teng M, Zhang X, et al. miR2Disease: a manually curated database for microRNA deregulation in human disease. Nucleic Acids Research 2009 Jan;37(Database issue):D98–104. doi: 10.1093/nar/gkn714.
23. Hombach S, Kretz M. Non-coding RNAs: Classification, Biology and Functioning. Advances in Experimental Medicine and Biology 2016;937:3–17. doi: 10.1007/978-3-319-42059-2_1.
24. Cui C, Zhong B, Fan R, Cui Q. HMDD v4.0: a database for experimentally supported human microRNA-disease associations. Nucleic Acids Research 2024 Jan;52(D1):D1327–D1332. doi: 10.1093/nar/gkad717.
25. Kozomara A, Birgaoanu M, Griffiths-Jones S. miRBase: from microRNA sequences to function. Nucleic Acids Research 2019 Jan;47(D1):D155–D162. doi: 10.1093/nar/gky1141.
26. Lin X, Lu Y, Zhang C, Cui Q, Tang YD, Ji X, et al. LncRNADisease v3.0: an updated database of long non-coding RNA-associated diseases. Nucleic Acids Research 2024 Jan;52(D1):D1365–D1369. doi: 10.1093/nar/gkad828.
27. Glažar P, Papavasileiou P, Rajewsky N. circBase: a database for circular RNAs. RNA (New York, NY) 2014 Nov;20(11):1666–1670. doi: 10.1261/rna.043687.113.
28. Frankish A, Carbonell-Sala S, Diekhans M, Jungreis I, Loveland JE, Mudge JM, et al. GENCODE: reference annotation for the human and mouse genomes in 2023. Nucleic Acids Research 2023 Jan;51(D1):D942–D949. doi: 10.1093/nar/gkac1071.
29. Zhao Y, Li H, Fang S, Kang Y, Wu W, Hao Y, et al. NONCODE 2016: an informative and valuable data source of long non-coding RNAs. Nucleic Acids Research 2016 Jan;44(D1):D203–208. doi: 10.1093/nar/gkv1252.
30. Wang J, Zhang P, Lu Y, Li Y, Zheng Y, Kan Y, et al. piRBase: a comprehensive database of piRNA sequences. Nucleic Acids Research 2019 Jan;47(D1):D175–D180. doi: 10.1093/nar/gky1043.
31. Piuco R, Galante PAF, piRNAdb: A piwi-interacting RNA database; 2021. doi: 10.1101/2021.09.21.461238.
32. Schriml LM, Munro JB, Schor M, Olley D, McCracken C, Felix V, et al. The Human Disease Ontology 2022 update. Nucleic Acids Research 2022 Jan;50(D1):D1255–D1261. doi: 10.1093/nar/gkab1063.
33. Ji Y, Zhou Z, Liu H, Davuluri RV. DNABERT: pre-trained Bidirectional Encoder Representations from Transformers model for DNA-language in genome. Bioinformatics (Oxford, England) 2021 Aug;37(15):2112–2120. doi: 10.1093/bioinformatics/btab083.
34. Sanabria M, Hirsch J, Joubert PM, Poetsch AR. DNA language model GROVER learns sequence context in the human genome. Nature Machine Intelligence 2024 Jul;6(8):911–923. doi: 10.1038/s42256-024-00872-0.
35. Suzuki S, Horie K, Amagasa T, Fukuda N. Genomic language models with k-mer tokenization strategies for plant genome annotation and regulatory element strength prediction. Plant Molecular Biology 2025 Jul;115(4):100. doi: 10.1007/s11103-025-01604-7.
36. Van Laarhoven T, Nabuurs SB, Marchiori E. Gaussian interaction profile kernels for predicting drug–target interaction. Bioinformatics 2011 Nov;27(21):3036–3043. doi: 10.1093/bioinformatics/btr500.
37. Köhler S. Improved ontology-based similarity calculations using a study-wise annotation model. Database 2018 Jan;2018. doi: 10.1093/database/bay026.
38. Mathur S, Dinakarpandian D. Finding disease similarity based on implicit semantic similarity. Journal of Biomedical Informatics 2012 Apr;45(2):363–371. doi: 10.1016/j.jbi.2011.11.017.
39. Jin C, Shi Z, Lin K, Zhang H. Predicting miRNA-Disease Association Based on Neural Inductive Matrix Completion with Graph Autoencoders and Self-Attention Mechanism. Biomolecules 2022 Jan;12(1):64. doi: 10.3390/biom12010064.
40. Lou Z, Cheng Z, Li H, Teng Z, Liu Y, Tian Z. Predicting miRNA–disease associations via learning multimodal networks and fusing mixed neighborhood information. Briefings in Bioinformatics 2022 Sep;23(5):bbac159. doi: 10.1093/bib/bbac159.
41. Wang L, Zhong C. gGATLDA: lncRNA-disease association prediction based on graph-level graph attention network. BMC Bioinformatics 2022 Jan;23(1):11. doi: 10.1186/s12859-021-04548-z.
42. Wang MN, You ZH, Wang L, Li LP, Zheng K. LDGRNMF: LncRNA-disease associations prediction based on graph regularized non-negative matrix factorization. Neurocomputing 2021 Feb;424:236–245. doi: 10.1016/j.neucom.2020.02.062.
43. Wei H, Xu Y, Liu B. iPiDi-PUL: identifying Piwi-interacting RNA-disease associations based on positive unlabeled learning. Briefings in Bioinformatics 2021 May;22(3):bbaa058. doi: 10.1093/bib/bbaa058.
44. Chen Q, Zhang L, Liu Y, Qin Z, Zhao T. PUTransGCN: identification of piRNA–disease associations based on attention encoding graph convolutional network and positive unlabelled

learning. Briefings in Bioinformatics 2024 Mar;25(3):bbae144. doi: 10.1093/bib/bbae144.
45. Lan W, Dong Y, Chen Q, Liu J, Wang J, Chen YPP, et al. IGNSCDA: Predicting CircRNA-Disease Associations Based on Improved Graph Convolutional Network and Negative Sampling. IEEE/ACM Transactions on Computational Biology and Bioinformatics 2022 Nov;19(6):3530–3538. doi: 10.1109/TCBB.2021.3111607.
46. Peng L, Yang C, Chen Y, Liu W. Predicting CircRNA-Disease Associations via Feature Convolution Learning With Heterogeneous Graph Attention Network. IEEE Journal of Biomedical and Health Informatics 2023 Jun;27(6):3072–3082. doi: 10.1109/JBHI.2023.3260863.
47. Jiang J, Lu J, Wang X, Sun B, Liu X, Ding Y, et al. Glioma stem cell-derived exosomal miR-944 reduces glioma growth and angiogenesis by inhibiting AKT/ERK signaling. Aging 2021 Jul;13(15):19243–19259. doi: 10.18632/aging.203243.
48. Wang D, Zhi T, Xu X, Bao Z, Fan L, Li Z, et al. MicroRNA-936 induces cell cycle arrest and inhibits glioma cell proliferation by targeting CKS1. American Journal of Cancer Research 2017;
49. Feng L, Yang Z, Li Y, Pan Q, Zhang X, Wu X, et al. MicroRNA-378 contributes to osteoarthritis by regulating chondrocyte autophagy and bone marrow mesenchymal stem cell chondrogenesis. Molecular Therapy Nucleic Acids 2022 Jun;28:328–341. doi: 10.1016/j.omtn.2022.03.016.
50. Panagopoulos PK, Lambrou GI. The Involvement of MicroRNAs in Osteoarthritis and Recent Developments: A Narrative Review. Mediterranean Journal of Rheumatology 2018 Jun;29(2):67–79. doi: 10.31138/mjr.29.2.67.
51. Xue YF, Li M, Li W, Lin Q, Yu BX, Zhu QB, et al. Roles of circCSPP1 on the proliferation and metastasis of glioma cancer. European Review for Medical and Pharmacological Sciences 2020 May;24(10):5519–5525. doi: 10.26355/eurrev$_2$02005$_2$1337.
52. Liu H, Weng J, Huang CLH, Jackson AP. Is the voltage-gated sodium channel 3 subunit (SCN3B) a biomarker for glioma? Functional & Integrative Genomics 2024 Sep;24(5):162. doi: 10.1007/s10142-024-01443-7.
53. Dokumacioglu E, Duzcan I, Iskender H, Sahin A. RhoA/ROCK-1 Signaling Pathway and Oxidative Stress in Coronary Artery Disease Patients. Brazilian Journal of Cardiovascular Surgery 2022 May;37(2):212–218. doi: 10.21470/1678-9741-2020-0525.
54. Holvoet P, Klocke B, Vanhaverbeke M, Menten R, Sinnaeve P, Raitoharju E, et al. RNA-sequencing reveals that STRN, ZNF484 and WNK1 add to the value of mitochondrial MT-COI and COX10 as markers of unstable coronary artery disease. PloS One 2019;14(12):e0225621. doi: 10.1371/journal.pone.0225621.
55. Wang S, Cheng Y, Yang P, Qin G. Silencing of Long Noncoding RNA LINC00324 Interacts with MicroRNA-3200-5p to Attenuate the Tumorigenesis of Gastric Cancer via Regulating BCAT1. Gastroenterology Research and Practice 2020;2020:4159298. doi: 10.1155/2020/4159298.
56. Liang W, Xia B, He C, Zhai G, Li M, Zhou J. Overexpression of LINC00691 promotes the proliferation and invasion of gastric cancer cells via the Janus kinase/signal transducer and activator of transcription signalling pathway. The International Journal of Biochemistry & Cell Biology 2020 Jun;123:105751. doi: 10.1016/j.biocel.2020.105751.
57. He C, Liu Y, Li J, Zheng X, Liang J, Cui G, et al. LncRNA RPSAP52 promotes cell proliferation and inhibits cell apoptosis via modulating miR-665/STAT3 in gastric cancer. Bioengineered 2022 Apr;13(4):8699–8711. doi: 10.1080/21655979.2022.2054754.
58. Shi X, Zhang H, Wang M, Xu X, Zhao Y, He R, et al. LncRNA AFAP1-AS1 promotes growth and metastasis of cholangiocarcinoma cells. Oncotarget 2017 Aug;8(35):58394–58404. doi: 10.18632/oncotarget.16880.
59. Li Y, Wu X, Gao H, Jin JM, Li AX, Kim YS, et al. Piwi-Interacting RNAs (piRNAs) Are Dysregulated in Renal Cell Carcinoma and Associated with Tumor Metastasis and Cancer-Specific Survival. Molecular Medicine (Cambridge, Mass) 2015 May;21(1):381–388. doi: 10.2119/molmed.2014.00203.
60. Schulze M, Sommer A, Plötz S, Farrell M, Winner B, Grosch J, et al. Sporadic Parkinson's disease derived neuronal cells show disease-specific mRNA and small RNA signatures with abundant deregulation of piRNAs. Acta Neuropathologica Communications 2018 Jul;6(1):58. doi: 10.1186/s40478-018-0561-x.
61. Roy J, Sarkar A, Parida S, Ghosh Z, Mallick B. Small RNA sequencing revealed dysregulated piRNAs in Alzheimer's disease and their probable role in pathogenesis. Molecular bioSystems 2017 Feb;13(3):565–576. doi: 10.1039/c6mb00699j.
62. Liu X, Lv X, Chen Q, Sun J, Zhao T, Zhu Y, Supporting data for "PanGIA: A universal framework for identifying association between ncRNAs and diseases". GigaScience Database; 2023. doi: 10.5524/102760.

Response to Reviewers

Click here to access/download
Supplementary Material
Review_Response_Letter.pdf
