# Supplementary material for: PanGIA: A universal framework for identifying association between ncRNAs and diseases
Source: Gigascience. 2025 Oct 17;14:giaf123. doi: 10.1093/gigascience/giaf123 (PMC12532321; doi:10.1093/gigascience/giaf123)
Supplement: giaf123_Reviewer_1_Report_Original_Submission — Veronica Buttaro -- 7/9/2025 [file giaf123_reviewer_1_report_original_submission.pdf]

Reviewer Report

Title: PanGIA: A universal framework for identifying association between ncRNAs and diseases

Version: Original Submission **Date:** 7/9/2025

Reviewer name: Veronica Buttaro

Reviewer Comments to Author:

Authors propose a novel computational framework, PanGIA , that simultaneously predict potential associations between multiple types of non-coding RNAsâ€"and diseases.The article presents an innovative work that brings together different types of ncRNA, filling a gap in the state of the art, as highlighted in the article itself.
While the architecture is solid, and it is also demonstrated by experimental results some parts (notably the sequence embedding) rely on established rather than cutting-edge methods. For example, the way in which RNA sequence embedding is generated is not exactly state-of-the-art, so it could be useful to compare state-of-the-art techniques or reuse existing techniques. Furthermore, experimental details are sometimes missing: there is no information on the size of this final embedding, even though the code is present. It is also unclear why similarity is calculated on strings rather than embeddings, given that these would be available and could be simpler. Please explain this choice, if possible, and clarify what is meant by "score" in the formula. Furthermore, the task (association) is not explained very well in terms of how it is performed and what the MLP predicts as output. It would also be useful to explain more explicitly the first step in the Feature Representation and Heterogeneous Graph
Modeling phase, namely the generation of node embeddings. Overall, the work has many strengths, including its methodology, important topic, and presentation of results with numerous ablation studies.

Methods

Are the methods appropriate to the aims of the study, are they well described, and are necessary controls included? Choose an item.

Conclusions

Are the conclusions adequately supported by the data shown? Choose an item.

Reporting Standards

Does the manuscript adhere to the journal's guidelines on minimum standards of reporting? Choose an item.

Choose an item.

Statistics

Are you able to assess all statistics in the manuscript, including the appropriateness of statistical tests used? Choose an item.

Quality of Written English

Please indicate the quality of language in the manuscript: Choose an item.

Declaration of Competing Interests

Please complete a declaration of competing interests, considering the following questions:

- Have you in the past five years received reimbursements, fees, funding, or salary from an organisation that may in any way gain or lose financially from the publication of this manuscript, either now or in the future?
- Do you hold any stocks or shares in an organisation that may in any way gain or lose financially from the publication of this manuscript, either now or in the future?
- Do you hold or are you currently applying for any patents relating to the content of the manuscript?
- Have you received reimbursements, fees, funding, or salary from an organization that holds or has applied for patents relating to the content of the manuscript?
- Do you have any other financial competing interests?
- Do you have any non-financial competing interests in relation to this paper?

If you can answer no to all of the above, write 'I declare that I have no competing interests' below. If your reply is yes to any, please give details below.

I declare that I have no competing interests

I agree to the open peer review policy of the journal. I understand that my name will be included on my report to the authors and, if the manuscript is accepted for publication, my named report including any attachments I upload will be posted on the website along with the authors' responses. I agree for my report to be made available under an Open Access Creative Commons CC-BY license (http://creativecommons.org/licenses/by/4.0/). I understand that any comments which I do not wish to be included in my named report can be included as confidential comments to the editors, which will not be published.

Choose an item.

To further support our reviewers, we have joined with Publons, where you can gain additional credit to further highlight your hard work (see: https://publons.com/journal/530/gigascience). On publication of this paper, your review will be automatically added to Publons, you can then choose whether or not to claim your Publons credit. I understand this statement.

Yes Choose an item.
