# Supplementary material for: PanGIA: A universal framework for identifying association between ncRNAs and diseases
Source: Gigascience. 2025 Oct 17;14:giaf123. doi: 10.1093/gigascience/giaf123 (PMC12532321; doi:10.1093/gigascience/giaf123)
Supplement: giaf123_Reviewer_2_Report_Original_Submission — Wei Lan -- 7/14/2025 [file giaf123_reviewer_2_report_original_submission.pdf]

Reviewer Report

Title: PanGIA: A universal framework for identifying association between ncRNAs and diseases

Version: Original Submission **Date:** 7/14/2025

Reviewer name: Wei Lan

Reviewer Comments to Author:

Liu et al. propose PanGIA, a novel and forward-looking computational framework for predicting associations between multiple types of non-coding RNAs (miRNA, lncRNA, circRNA, and piRNA) and diseases. By integrating a heterogeneous graph attention network (HAN) with a mixture-of-experts (MoE) architecture, PanGIA effectively captures complex cross-modal relationships derived from sequence features, functional annotations, and interaction networks, demonstrating strong capabilities in model design and multi-source data integration. Through comprehensive benchmarking and case study validation, the study shows superior performance in terms of accuracy, robustness, and generalizability compared to existing single-type prediction methods.This work not only presents significant methodological innovation, but also offers a new perspective and tool for the systematic investigation of ncRNA-disease associations. As an important advancement in the field, PanGIA holds great potential for promoting future research in disease mechanism exploration, biomarker discovery, and precision medicine. Below are my specific comments and suggestions on the manuscript:

1.The authors have integrated multiple data sources in the PanGIA framework, including sequence information, functional annotations, and interaction networks. Could the authors elaborate on whether any specific algorithms or tools were employed to ensure consistency and compatibility among these heterogeneous data types? Additionally, is there a more detailed explanation of the data preprocessing steps, such as normalization or dimensionality reduction?

2.The PanGIA model incorporates heterogeneous graph attention mechanisms and expert modules, reflecting careful consideration of the heterogeneity among different ncRNA types. From a modeling perspective, could the authors briefly explain why a heterogeneous graph structure was adopted instead of a more conventional unified graph? Was this choice primarily intended to better preserve the distinct characteristics of each ncRNA type? Additionally, how does this design balance computational efficiency with overall model performance?

3.The paper adopts a hierarchical processing strategy for ncRNA sequences, involving local fragment-level feature extraction followed by global sequence-level aggregation. Could the authors clarify the main challenges this design aims to address? In terms of balancing computational complexity and feature representation completeness, what are the notable advantages of this segmented approach compared to modeling the full sequence directly?

4.In the case studies, PanGIA successfully predicted and retrospectively validated multiple ncRNA-disease associations, demonstrating its potential for biological applications. Have the authors considered how these predictions might be used to guide future experimental designs, such as target gene validation, pathway analysis, or functional studies of ncRNAs? A brief discussion in this regard could help highlight the practical value of the model in biomedical research.

5.PanGIA constructs a full heterogeneous graph across ncRNA types for joint training. In large-scale data settings or online prediction scenarios, have the authors considered strategies such as subgraph sampling or graph pruning to improve inference efficiency? Particularly in real-time or incremental learning environments, how scalable and deployable is PanGIA in practice?

Methods

Are the methods appropriate to the aims of the study, are they well described, and are necessary controls included? Choose an item.

Conclusions

Are the conclusions adequately supported by the data shown? Choose an item.

Reporting Standards

Does the manuscript adhere to the journal's guidelines on minimum standards of reporting? Choose an item.

Choose an item.

Statistics

Are you able to assess all statistics in the manuscript, including the appropriateness of statistical tests used? Choose an item.

Quality of Written English

Please indicate the quality of language in the manuscript: Choose an item.

Declaration of Competing Interests

Please complete a declaration of competing interests, considering the following questions:

- Have you in the past five years received reimbursements, fees, funding, or salary from an organisation that may in any way gain or lose financially from the publication of this manuscript, either now or in the future?
- Do you hold any stocks or shares in an organisation that may in any way gain or lose financially from the publication of this manuscript, either now or in the future?
- Do you hold or are you currently applying for any patents relating to the content of the manuscript?
- Have you received reimbursements, fees, funding, or salary from an organization that holds or has applied for patents relating to the content of the manuscript?
- Do you have any other financial competing interests?

- Do you have any non-financial competing interests in relation to this paper?

If you can answer no to all of the above, write 'I declare that I have no competing interests' below. If your reply is yes to any, please give details below.

I declare that I have no competing interests

I agree to the open peer review policy of the journal. I understand that my name will be included on my report to the authors and, if the manuscript is accepted for publication, my named report including any attachments I upload will be posted on the website along with the authors' responses. I agree for my report to be made available under an Open Access Creative Commons CC-BY license (http://creativecommons.org/licenses/by/4.0/). I understand that any comments which I do not wish to be included in my named report can be included as confidential comments to the editors, which will not be published.

Choose an item.

To further support our reviewers, we have joined with Publons, where you can gain additional credit to further highlight your hard work (see: https://publons.com/journal/530/gigascience). On publication of this paper, your review will be automatically added to Publons, you can then choose whether or not to claim your Publons credit. I understand this statement.

Yes Choose an item.
